# Counterion Enhanced Organocatalysis: A Novel Approach for the Asymmetric Transfer Hydrogenation of Enones

**DOI:** 10.1002/cctc.202000414

**Published:** 2020-06-15

**Authors:** Fabian Scharinger, Ádám Márk Pálvölgyi, Veronika Zeindlhofer, Michael Schnürch, Christian Schröder, Katharina Bica‐Schröder

**Affiliations:** ^1^ Institute of Applied Synthetic Chemistry TU Wien Getreidemarkt 9/163 1060 Wien Austria; ^2^ Department of Computational Biological Chemistry University of Vienna Währinger Str. 17 1090 Wien Austria

**Keywords:** organocatalysis, asymmetric synthesis, transfer hydrogenation, ion aggregation, phosphoric acid, counterion catalysis

## Abstract

We present a novel strategy for organocatalytic transfer hydrogenations relying on an ion‐paired catalyst of natural l‐amino acids as main source of chirality in combination with racemic, atropisomeric phosphoric acids as counteranion. The combination of a chiral cation with a structurally flexible anion resulted in a novel chiral framework for asymmetric transfer hydrogenations with enhanced selectivity through synergistic effects. The optimized catalytic system, in combination with a Hantzsch ester as hydrogen source for biomimetic transfer hydrogenation, enabled high enantioselectivity and excellent yields for a series of α,β‐unsaturated cyclohexenones under mild conditions. Moreover, owing to the use of readily available and chiral pool‐derived building blocks, it could be prepared in a straightforward and significantly cheaper way compared to the current state of the art.

## Graphical abstract

**Snap it with organocat**: We present a novel strategy for organocatalytic transfer hydrogenations relying on an ion‐paired catalyst of natural l‐amino acids as main source of chirality in combination with racemic, atropisomeric phosphoric acids as counteranion.

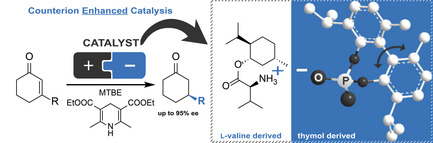

## Introduction

Asymmetric transfer hydrogenation has emerged as a powerful and convenient tool for the reduction of prochiral carbonyl compounds.^1^ The selective reduction of enones is a particularly challenging task due to the inherent question of regio‐ and stereoselectivity.^2^ Among many catalytic protocols that have been described for this particular reaction, the flourishing area of organocatalysis offers attractive solutions, and iminium‐based asymmetric transfer hydrogenations provide an efficient and metal‐free alternative for the selective reduction of enones via 1,4‐addition.^3^, ^4^, ^5^

**Figure 1:**
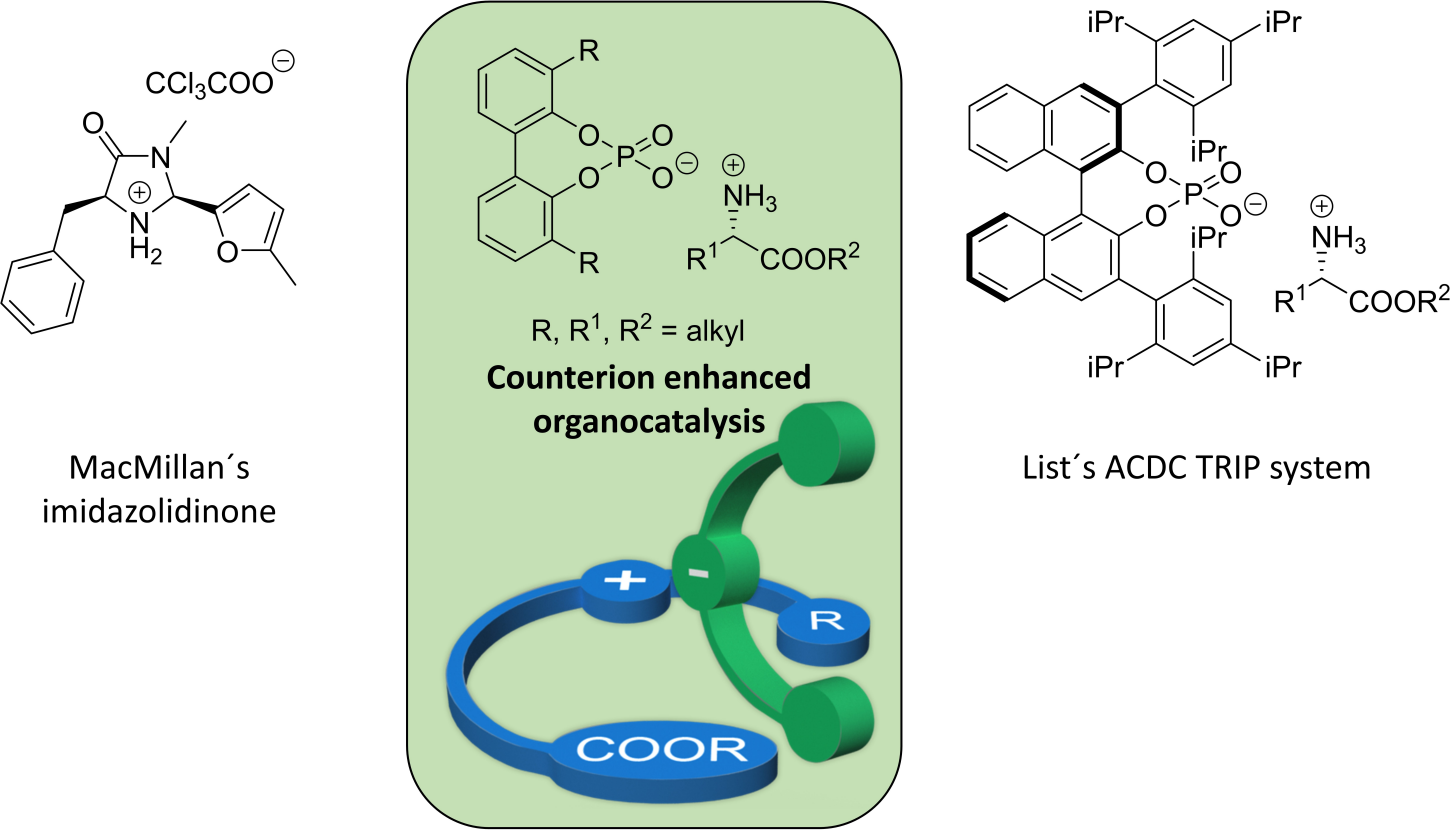
Iminium‐based organocatalytic reduction of enones and different catalytic systems, including MacMillan's imidazolidinone catalyst (left), List's TRIP phosphate (right) as well as the novel counterion enhanced approach reported in here (middle).

Over the past years, two types of catalysts have emerged as particularly well suited for organocatalytic transfer hydrogenations of enals and enones. In 2004, the group of MacMillan reported a series of imidazolidinone derivatives as powerful benchmark catalysts for highly asymmetric conjugate hydrogenations of α,β‐unsaturated aldehydes and ketones (Figure 1, left).^5^, ^6^ In parallel, List and co‐workers described a novel method for the non‐asymmetric transfer hydrogenation of cinnamaldehyde derivatives using secondary ammonium salts as catalysts.^7^ Two years later, a new methodology in asymmetric transfer hydrogenation was reported by Mayer and List as they observed high catalytic activity and enantioselectivity in the reaction of enals and enones catalyzed by ammonium salts composed of an achiral or chiral cation and an enantiopure sterically demanding phosphate anion, TRIP (Figure 1, right).^8^, ^9^ Based on the early success of List's catalytic system, a new general concept emerged in the field of organocatalysis, known as Asymmetric Counteranion‐Directed Catalysis (ACDC), which refers to any catalytic reaction, in which the enantiodiscrimination is induced through the tight ion‐pairing of a cationic intermediate with an enantiomerically pure anion.^10^ The ion‐bound nature of this attractive approach for chiral induction offers considerable flexibility for fine‐tuning of the electronic and steric properties compared to conventional covalent ligand systems.^11^ Since the discovery of ACDC, or in general, of asymmetric ion‐pairing catalysis, several other highly enantioselective reactions proceeding through cationic intermediates have been reported.^12^, ^13^, ^14^, ^15^ While different asymmetric induction modes can be realized in asymmetric ion‐pairing catalysis, the formation of ion pairs between ammonium cations and bulky chiral phosphate anions is particularly prominent.^16^, ^17^, ^18^, ^19^, ^20^, ^21^

While these elegant strategies are certainly among the most important discoveries in the field of organocatalysis for the last decades, they still do have some limitations. MacMillan's imidazolidinones are excellent catalysts for the asymmetric transfer hydrogenation of enals; however, the reactivity for enones is inferior thus requiring higher catalyst loadings.^6^ In contrast, List et al. could successfully reach high reactivity and selectivity for the asymmetric transfer hydrogenation of enals and enones even with low catalyst loading based on the chiral TRIP counteranion. However, this approach requires a rather expensive catalyst, prepared in a five‐step synthesis with rather low overall yields. Moreover, a careful structural optimization is required for the bulky phosphate anion of the catalyst: while the TRIP counteranion provides indeed high selectivity in the ATH of ketones and aldehydes, a significant decrease in the enantiodiscrimination could be observed just with a slight modification of the phosphate unit, resulting in moderate selectivity or even close‐to‐racemic products. Given these limitations; a straightforward, cheaper and chiral‐pool derived catalytic system that combines high catalytic activity with excellent enantioselectivity would be highly desirable.

In here, we propose a novel concept of asymmetric ion‐paired organocatalysis with a fixed element of chirality based on ion aggregation between a chiral amino acid cation and C_2_‐symmetric atropisomeric phosphate anion. This concept of an ion‐paired catalyst framework offers novel opportunities for ligand design with unprecedented flexibility compared to conventional ligand design relying on covalently constructed ligands. Eventually, this unique approach results in an easily accessible, cheap and chiral pool‐derived a catalytic system that is yet able to reduce a set of α,β‐unsaturated ketones with high yields and selectivity.

## Results and Discussion

**Scheme 1:**
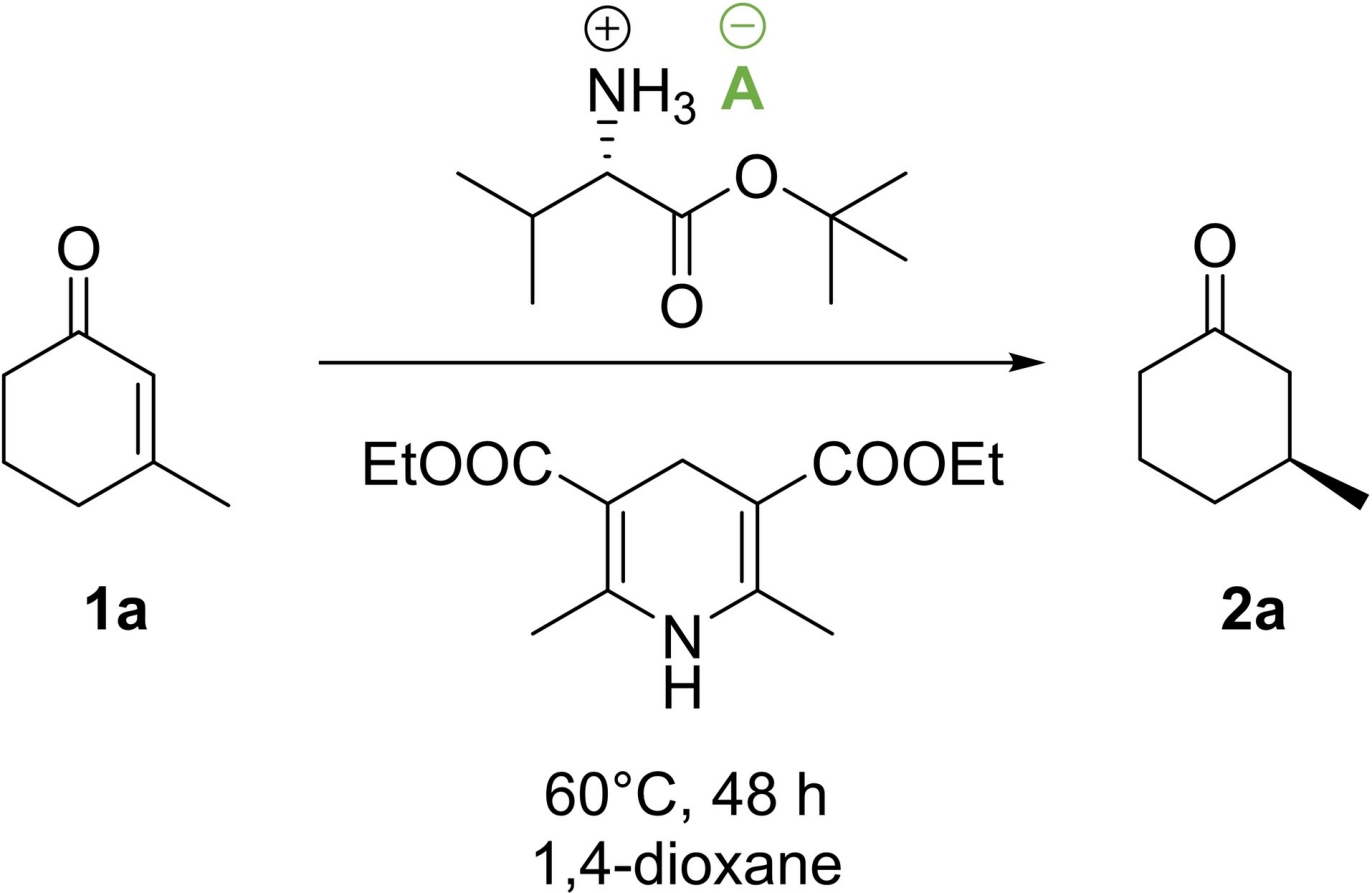
Proof of concept: asymmetric transfer hydrogenation of 3‐methyl‐2‐cyclohexenone (**1 a**) by using different salts of l‐valine *t*‐butyl ester.

To prove the concept of counterion enhanced asymmetric transfer hydrogenations, we initially focused on the reduction of 3‐methyl‐2‐cyclohexenone. The reaction was performed with the common Hantzsch ethyl ester as mild reductant using standard conditions that have been previously established by List et al (Scheme 1).^5^

For a first evaluation of the concept, different salts of (l)‐valine *t*‐butyl ester, including the non‐chiral trifluoroacetate salt, but also salts with tropoisomeric biphenyl phosphate as well as enantiopure or racemic binaphthyl phosphate anions were used as catalysts (Table 1). While no reaction was observed with the biphenolate anion **3** due to the lower acidity of 2,2′‐biphenol (Table 1, entry 1), the reaction proceeded with moderate yield in the presence of biphenyl phosphate **4** (Table 1, entries 4 and 5). Most importantly, an increase of enantioselectivity to 64 %ee – an increase by 10 %ee compared to the trifluoroacetate salt ‐ could be observed, indicating that the tropoisomeric and C2‐symmetric biphenyl phosphate anion plays a role in enantiodiscrimination (Table 1, entries 2 vs. 4). It is worthwhile noticing that this is not the case with different binaphthyl phosphate anions, as the observed enantioselectivity with racemic or enantiopure binaphthyl phosphate was identical to the values obtained for trifluoroacetate (Table 1, entries 2 vs. 6–8). This clearly shows that matched/mismatched effects between the chiral amino acid and the counteranion do not influence the enantioselectivity of the product.

**Table 1:** Proof of concept in the counterion enhanced asymmetric transfer hydrogenation of 3‐methyl‐2‐cyclohexenone (**1 a**) using (l)‐valine *t*‐butyl ester as cation source.

Entry^[a]^	Anion		Conv.^[c]^[%]	Yield^[c]^ [%]	ee^[c]^ [%]	Δee^[d]^
1	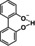	**3**	<1	<1	n.d.	n.d.
2	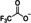		78	49	54	‐
3^[b]^	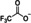		92	88	51	‐
4	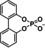	**4**	40	27	64	10
**5** ^[b]^	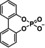	**4**	**75**	**72**	**74**	**23**
6	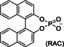	**8**	13	13	54	0
7	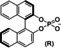	**8**	22	13	54	0
8	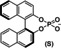	**8**	20	15	54	0

[a] Performed with 0.18 mmol 3‐methyl‐2‐cyclohexenone (**1 a**), 20 mol % catalyst and 0.22 mmol Hantzsch ethyl ester in 0.55 mL 1,4‐dioxane for 48 hours at 60 °C, [b] MTBE used as solvent, [c] Determined by GC analysis on BGB5 column using *n*‐dodecane as internal standard and chiral GC analysis using a BGB175 chiral capillary column, [d] Δee defined as the difference between the reaction with phosphate anion **4** and trifluoracetate anion in 1,4‐dioxane and MTBE, respectively.

Further studies showed that this effect is even more pronounced in MTBE as a solvent, where an enantioselectivity of 74 %ee compared to 51 %ee obtained with the trifluoroacetate anion was found (Table 1, entries 3 vs. 5). In fact, when studying the impact of different solvents in this reaction, we found that the best selectivity is observed in solvents with low dielectric constants such as MTBE (ϵ=4.5) or toluene (ϵ=2.4), whereas lower enantioselectivity was found in more polar solvents such as water (ϵ=80.1), see ESI Table S2, page S24 for details.

This increase in the enantioselectivity can be attributed to the solvent's polarity, resulting in the formation of contact ion pairs in apolar solvents and hence a stronger interaction between cation and anion in the catalyst. In contrast to conventional salts that are defined by a strictly charge‐ordered structure of atomic ions without dipole moment, organic salts, e. g. ionic liquids, possess a complex network with remarkable structural heterogeneity of the composing molecular ions.^22^ On a supramolecular length scale, ion pairs, or more precisely ion aggregates exist for a lifetime of a few picoseconds; however, ion pair destabilization occurs as pairs readily dissociate into individual ions in pure salt melts. This situation changes drastically when organic salts are dissolved in molecular solvents: A number of experimental techniques, including NMR measurements or cyclic voltammetry proved the existence of long‐lived ion pairs, suggesting that the ion pair formation is insignificant in neat salts but dominant in electrolyte solutions. Although a key feature of organic salts in solution, ion aggregation has been scarcely exploited for interionic chirality transfer. An outstanding example in this regard was published by Leitner and co‐workers in their work on asymmetric hydrogenations featuring a proline‐based chiral ionic liquid as solvent or additive in combination with atropisomeric phosphine ligands.^23^ Impressive enantioselectivity was obtained through a complex of [{(*R*)‐binap}Rh{(*S*)‐MeProl}]^+^, accompanied by *N*‐bis‐(trifluoromethane)sulfonimide anion.

**Figure 2:**
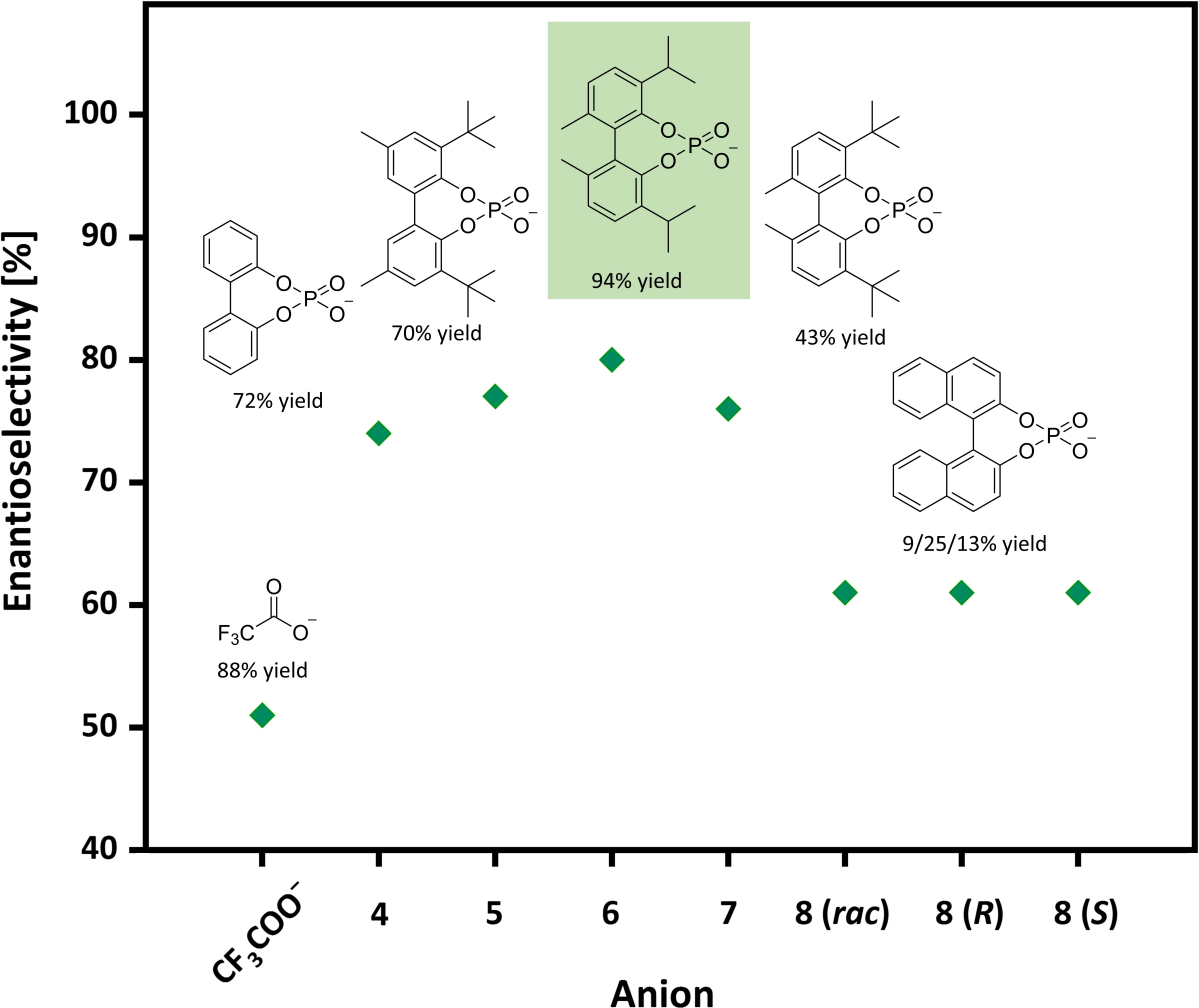
Impact of different phosphate anions vs. TFA anion on the enantioselectivity. All reactions were carried out in MTBE at 60 °C using l‐valine *t*‐butyl ester as cation source.

We reasoned that the catalytic system could be improved by tuning the steric demand and rotation barrier of the atropisomeric phosphate anion. For this purpose, a set of biphenyl phosphoric acids were prepared via microwave‐assisted oxidative coupling of phenols with *t*‐butyl peroxide. This straightforward coupling step, followed by reaction with POCl_3_ and successive hydrolysis gave access to the phosphoric acids (**4**–**7**) with variable substituent pattern in acceptable overall yield, starting from cheap and readily available phenols (see ESI S10–S13 for details). With this set of phosphoric acids in hand, the selectivity in asymmetric transfer hydrogenation could be considerably improved and varied between 74 and 80 %ee. Best results were obtained with the isopropyl substituted phosphoric acid **6** as anion source, providing up to 80 %ee with nearly quantitative yield (Figure 2, also see ESI Table S3, page S25 for details). Furthermore, other acids traditionally used in organocatalysis have been investigated. The ee values were below 60 % for all cases under the same reaction conditions, further highlighting the benefits of the atropisomeric phosphoric acids **4**–**7** (see ESI Table S4, page S26 for details).

**Scheme 2:**
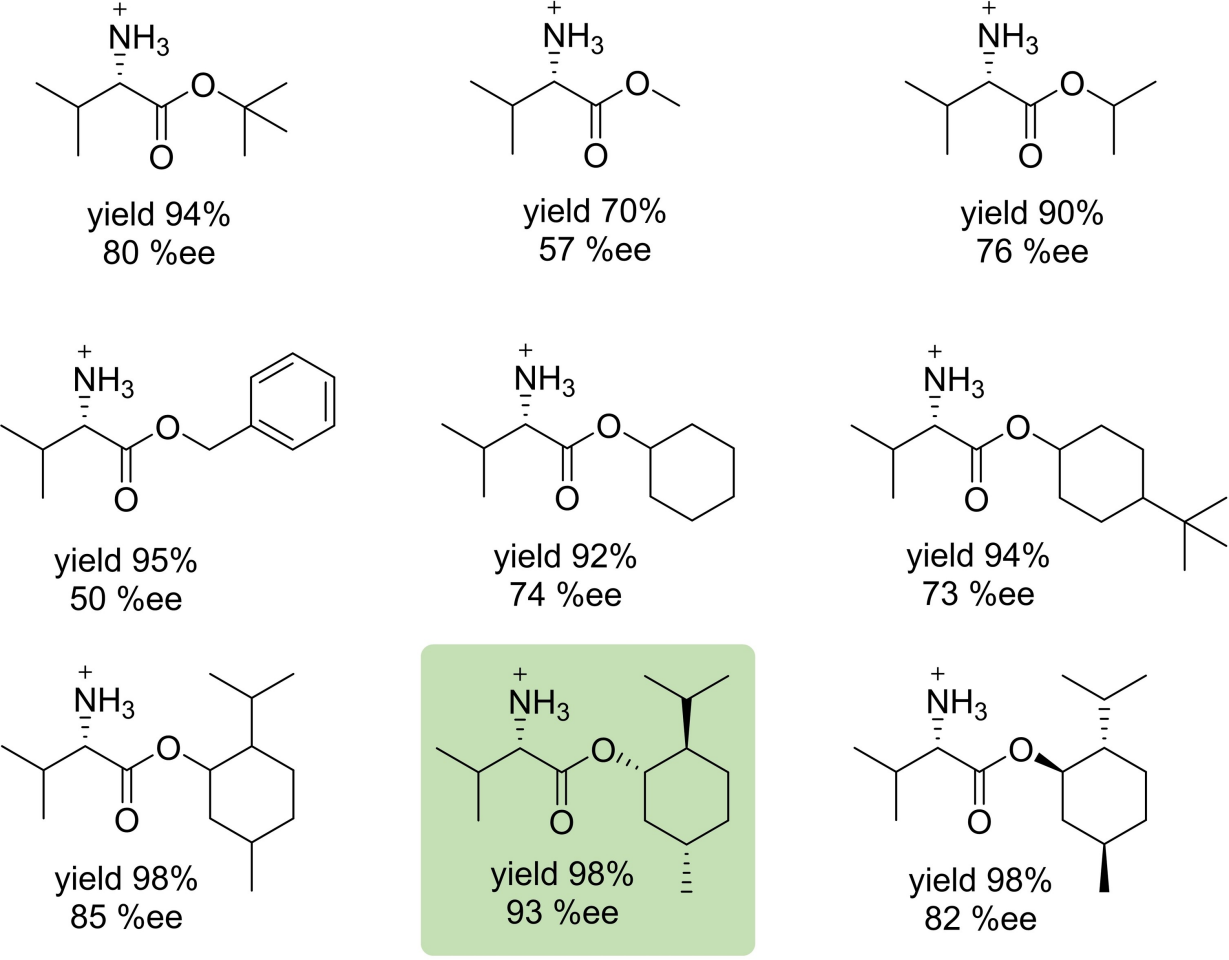
Optimization of the (l)‐valine ester moiety as cations source for. All reactions were carried out in MTBE at 60 °C using phosphate anion **6**.

Successively, we performed an extensive screening of the amino acid as cation source for further optimization of the catalytic system. Little difference in enantioselectivity (between 72–80 %ee) were found between (l)‐valine, (l)‐leucine, (l)‐isoleucine and (l)‐*t*‐leucine *t*‐butyl esters, whereas aromatic species such as (l)‐phenylalanine *t*‐butyl ester where less efficient. (l)‐Proline *t*‐butyl ester with a secondary ammonium cation gave also significantly lower selectivity (see ESI Table S5, page S27 for details). Finally, modifications on the ester moiety of (l)‐valine revealed that sterically demanding aliphatic groups such as *t*‐butyl, 4‐*t*‐butyl cyclohexyl or menthyl are indeed required for reaching high selectivity, as can be easily seen when comparing the results with those obtained for methyl or benzyl esters. Eventually, the best results were obtained when matching (l)‐valine with the (+)‐(1*S*,2*R*,5*S*) menthol, as the resulting ester in combination with phosphoric acid **6** resulted in an excellent yield of 98 % and an enantioselectivity of 93 %ee. This is in contrast to the mismatched ester‐system obtained with (−)‐(1*R*,2*S*,5*R*) menthol, which gave only 82 %ee (Scheme 2).

With the ideal cationic and anionic moiety identified, it was also possible to reduce the catalyst loading to lower amounts: When studying different catalyst loadings at 25 °C, the high selectivity was maintained even at 5 mol % catalyst, although losses in yield had to be taken into account. Eventually, a reaction temperature of 50 °C provided an ideal compromise between yield and selectivity, providing the desired product **2 a** in 98 % yield and 95 %ee (Table 2, entry 9).

**Table 2:** Optimization of conditions and parameters for the asymmetric transfer hydrogenation of 3‐methyl‐2‐cyclohexenone (**1 a**) using (*l*)‐valine (+)‐(1*R*,3*R*,4*S*) menthyl ester and the isopropyl substituted phosphoric acid **6** as anion source.

Entry^[a]^	T [°C]	Catalyst [mol %]	Hantzsch ester	Yield^[b]^/%	ee^[c]^ [%]
1	60	20	ethyl	98	93
2	50	20	ethyl	98	95
3	40	20	ethyl	96	95
4	25	20	ethyl	59	96
5	25	10	ethyl	43	96
6	25	5	ethyl	29	96
7	25	1	ethyl	11	96
8	50	10	ethyl	99	94
**9**	**50**	**5**	**ethyl**	**98**	**95**
10	50	1	ethyl	68	93
11^[d]^	25	20	methyl	14	83
12^[d]^	25	20	ethyl	17	88
13^[d]^	25	20	*i*‐propyl	58	69
14^[d]^	25	20	*t*‐butyl	58	67

[a] Performed with 0.18 mmol 3‐methyl‐2‐cyclohexenone, 0.009‐0.036 mmol catalyst and 0.22 mmol Hantzsch ester in 0.55 mL MTBE at the given temperature for 48 hours, [b] Determined by GC analysis on a BGB5 column using *n*‐dodecane as internal standard, [c] Determined by chiral GC analysis using a BGB175 chiral capillary column, [d] Performed with (l)‐valine *t*‐butyl ester instead of (l)‐valine (+)‐(1*R*,3*R*,4*S*) menthyl ester.

For further studies on scope and limitation of the newly established catalytic system, the substrate pool was widened to investigate the asymmetric transfer hydrogenation of different 3‐substituted cyclohexenones under the previously optimized reaction conditions (Table 3). Enantioselectivity exceeded 90 % frequently, thereby demonstrating the versatility and broad application range of the newly established system.

**Table 3:** Substrate scope for the counterion enhanced transfer hydrogenation of enones.

Entry^[a]^	Substrate		Yield ^[d]^ [%]	ee^[e]^ [%]
1	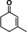	**1 a**	98 (72)^[f]^	95 (*S*)
2	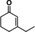	**1 b**	85 (76)^[f]^	92 (*S*)
3	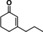	**1 c**	70 (55)^[f]^	91 (*S*)
4	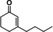	**1 d**	91 (87)	93 (*S*)
5	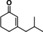	**1 e**	85 (60)^[f]^	86 (*S*)
6	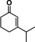	**1 f**	75 (68)^[f]^	88 (*S*)
7^[b]^	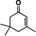	**1 g**	66 (60)	92 (*S*)
8^[c]^	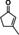	**1 h**	94 (80)^[f]^	89 (*S*)
9	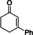	**1 i**	86 (84)	82 (*S*)
10	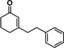	**1 j**	94 (91)	90 (*S*)
11	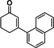	**1 k**	65 (62)	94 (*S*)
12	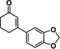	**1 l**	89 (86)	70 (*S*)

[a] Performed with 1.8 mmol ketone, 5 mol % catalyst and 2.2 mmol Hantzsch ethyl ester in 5.5 mL MTBE at 50 °C for 48 hours, [b] 20 mol % catalyst, [c] 10 mol % catalyst, [d] Determined by GC or GC−MS analysis. Isolated yields after flash column chromatography are given in parenthesis, [e] Determined by chiral GC analysis using a BGB175 or BGB173 chiral capillary column, or by chiral HPLC analysis using a Diacel Chiralcel AS−H column. Absolute configurations have been determined by measuring the optical rotation and comparing with literature data, [f] Lower isolated yield because of high volatility of the product.

In general, atropisomerism is a type of axial chirality that may arise in systems where free rotation about a single covalent bond is hindered.^24^ The isomerization pathways of biphenyls with bulky ortho‐substituents were studied in detail by Masson, showing that conformers with isomerization barriers >23 kcal/mol can be separated at room temperature.^25^ Less information is available for biphenols or biphenyl phosphoric acids. For more insight on the behavior of the atropisomeric phosphoric acids, we calculated isomerization barriers for compounds **4**–**8** (Table 4). The pure electrostatic barriers were corrected by frequency contributions to result in the Gibbs free energy barrier (ΔG) at a temperature of 333.15 K. Optimizations, energy evaluations and frequency calculations were performed on the B3LYP‐D3/def2TZVP^26^, ^27^, ^28^ level of theory with the program package ORCA.^29^ Correlation in the uniform electron gas was modeled according to the Vosko‐Wilk‐Nusair VWN5 formalism.^30^ A detailed description of the computational methodology can be found in the ESI (S32–S33).

**Table 4:** Barrier energies for the phosphoric acids on the B3LYP‐D3/def2TZVP level of theory.

Entry	Biphenol/ Phosphoric acid		Isomerization barrier ΔG	Dihedral angle around the aryl‐aryl bound	
			[kcal/mol]	Ground state	Transition state
1	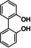		11.5^31^	110°	0°
2	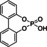	**4**	10.0	43°	0°
3	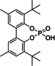	**5**	12.0	49°	0°
4	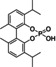	**6**	41.2	56°	21°
5	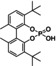	**7**	48.2	61°	29°
6	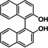		42.7^25^	75°	26°

In general, comparison of the isomerization barriers between biphenols and biphenyl phosphoric acids is not straightforward. In case of biphenol, at least five ground state isomers can be identified, resulting in multiple isomerization pathways.^31^ Sahnoun et al. estimate a value of 11.5 kcal/mol for the lowest possible pathway.^31^

**Figure 3:**
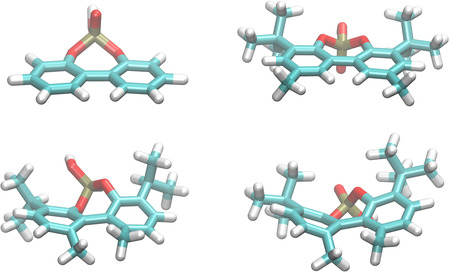
Optimized geometry of the transition states of compounds **4**–**7**.

Due to the additional O−P−O bridge, the phosphoric acids are restricted in the rotation of the aryl‐aryl bond, hence allowing only one pathway. The phosphoric acids **4** and **5** have similar rotational barriers of 10 and 12 kcal/mol, indicating that substituents in 3,3′‐position have only a minor impact on the rotation barrier. As visible from Figure 3 (top), their transition states for the isomerization are both planar. However, barriers for the acids **6** and **7** increase to 41.2 and 48.2 kcal/mol, respectively, which can be attributed to the methyl groups in 6,6′‐position. Apart from the steric hindrance of the rotation, this substitution pattern also forces an out‐of‐plane transition state (Figure 3, bottom).

Moreover, the optimal dihedral angle of 2,2′‐biphenol and biphenyl phosphoric acid differs significantly due to the constraints of the phosphate bridge on the rotation of the two phenyl rings (Table 4). In case of 2,2′‐biphenol two hydroxyl hydrogens are able to form hydrogen bonds to the opposite oxygen.

The rotational barrier itself cannot explain the different enantioselectivity observed in the ATH reaction. For example, anions arising from phosphoric acid **5** and **7** gave similar enantioselectivity, despite a considerable difference in their rotational barriers (Figure 2,). Additionally, the rotational barrier of the binaphthyl phosphate **8** is considerably higher compared to biphenyl phosphate **4**. However, the latter resulted in considerably higher yield and enantioselectivity (see Table 1, entries 4 vs. 6–8), indicating that a complex interplay of the steric demand, anion geometry and rotation barrier is responsible for the different performance in the ATH reaction.

**Figure 4:**
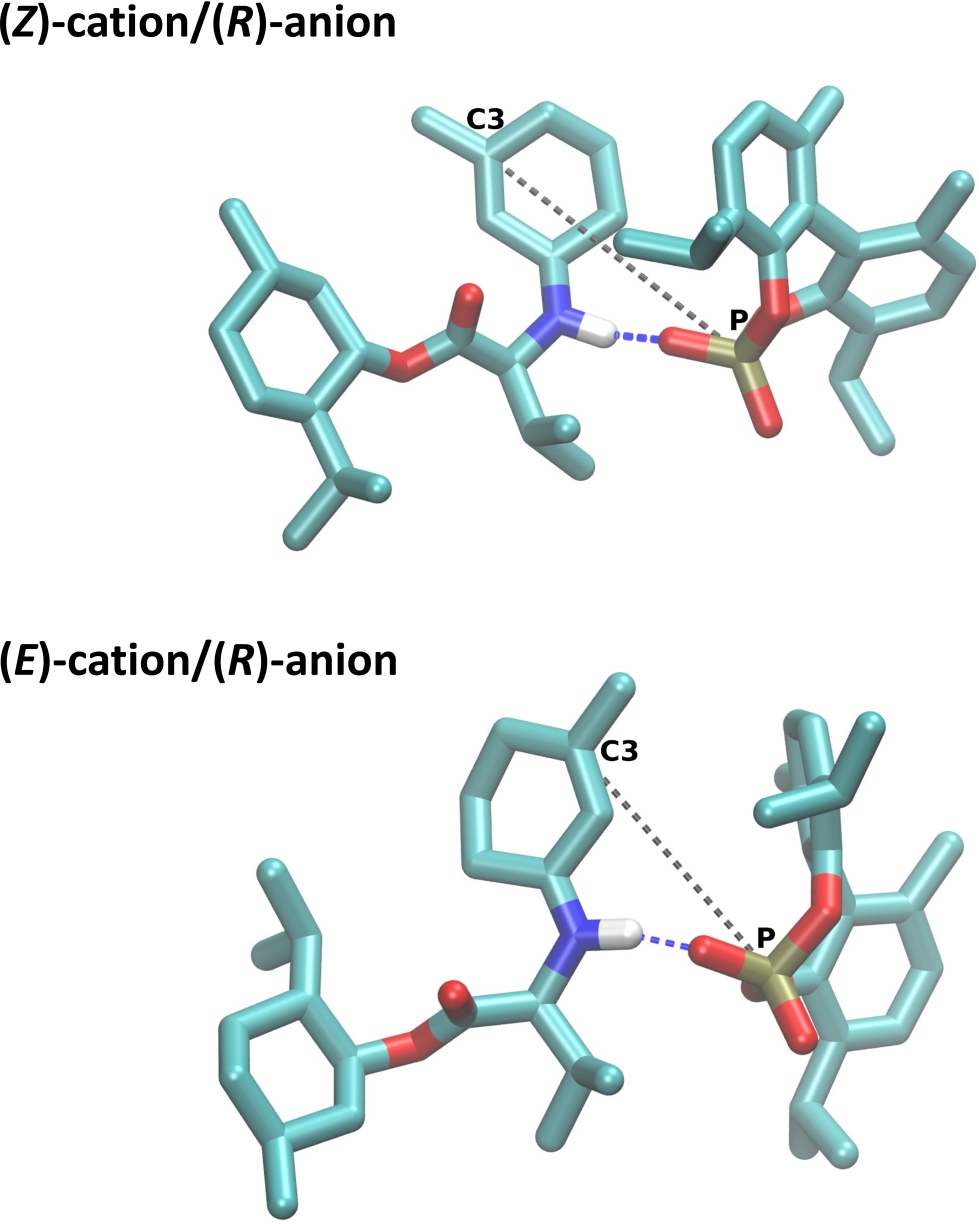
Snapshots of the (*Z*)‐cation/(*R*)‐anion (top) and (*E*)‐cation/(*R*)‐anion (bottom) pairs from the simulations. All solvent molecules as well as all hydrogens except the nitrogen‐bound hydrogen were omitted for clarity.

The most promising catalyst system was investigated further via non‐polarizable molecular dynamics simulations. To model the ion pair prior to the enantioselective reaction step, we further investigated the iminium cation formed (+)‐(1*S*,2*R*,5*S*) menthyl‐l‐valinate and 3‐methyl‐2‐cyclohexenone, in combination with phosphate anion **6**. Simulations were performed for one intermediate ion pair at 60 °C in methyl butyl ether, a solvent with a comparable dielectric constant to MTBE (see ESI S34‐S37 for details). To explore any structural difference in the ion pairs, the (*E*) and (*Z*) form of the cation and both enantiomers of the anion were considered in four different simulations. The ion pairs incorporating the (*R*)‐enantiomer of the anion are shown in Figure 4.

From the average interionic distance calculated respective to center of mass, it can be seen that the ions form stable pairs throughout the simulation. In all systems, the hydrogen bond between cation and anion (see Figure 4 and Table 5) is present for more than 85 % of the simulation time. This hydrogen bond promotes a certain orientation of the ion pair, with the phosphate group of the anion oriented towards the nitrogen of the cation. This also affects the average distance between the prochiral C3‐atom of the cation and the P‐atom of the anion. In the unfavored (*Z*)‐form, the methyl group bound to the prochiral C3 points on the opposite side of the nitrogen‐bound hydrogen, resulting in an interionic distance of approx. 6.5 Å away. In contrast, the C3‐P distance is shorter by about 1 Å in the favored (*E*)‐form, providing a closer ion pair and thus might be ideal for chiral induction.

**Table 5:** Average number of hydrogen bonds and interionic distances obtained from molecular dynamics simulations.

Ion pair	Average number of hydrogen bonds	Average interionic distance (center of mass) [Å]	Average distance C3‐P [Å]
Z‐cat/R‐an	0.90±0.04	7.2±0.8	6.7±0.5
Z‐cat/S‐an	0.85±0.07	6.9±0.9	6.6±0.6
E‐cat/R‐an	0.90±0.05	7.1±0.9	5.5±0.4
E‐cat/S‐an	0.87±0.03	6.8±0.9	5.2±0.4

## Conclusion

In here, we reported a novel concept of counterion catalysis for organocatalytic asymmetric transfer hydrogenations. The ion‐paired catalysts, based on cheap amino acid‐derived cations and flexible phosphate anions could be readily synthetized from natural compounds, providing a significantly cheaper alternative to the current start‐of‐art ACDC methodology. After careful parameter optimization, a series of different enones could be reduced with high yields and enantioselectivity under mild conditions even with low catalyst loadings.

From the simulation data, we can conclude that interionic interactions are strong enough to form a stable ion pair in apolar solvents such as MTBE, and that the hydrogen bond both favors a certain arrangement of the ion pair, with the C3 atom relatively close to the anion. The exact interplay between the ions, and the mechanism of a possible chirality transfer remains unclear, and it will be further evaluated in future studies addressing the influence of aggregate formation rather than a single ion pair.

Current investigations focus on the exploration of the reaction scope for counterion enhanced organocatalysis with chiral amino acid derived cations and atropisomeric phosphate anions. Overall, we expect that the concept of counterion enhanced catalysis relying on chiral cations and atropisomeric anions will find broad utility in organocatalysis, but also in transition metal catalyzed process.

## Experimental Section

### Representative procedure for the asymmetric transfer hydrogenation

A glass vial equipped with a magnetic stir bar was charged with ketone (1.8 mmol, 1.0 equiv.) in MTBE (5.5 mL, 0.33 M), followed by the addition of the catalyst (55.4 mg, 0.09 mmol, 5 mol %) and Hantzsch ester (552 mg, 2.2 mmol, 1.2 equiv.). The reaction mixture was stirred at 50 °C for 48 h. After cooling to room temperature, diethyl ether (5 mL) and 4 M HCl (10 mL) was added and the mixture was stirred until the phases were transparent (30 min). The phases were separated and the organic phase was washed with 4 M HCl (3×20 mL). The organic phase was dried over Na_2_SO_4_ and concentrated under reduced pressure. Purification by column chromatography (15 % Et_2_O: PE, vanillin staining agent or UV visualization) gave the 3‐substituted cyclohexanones.

## Conflict of interest

The authors declare no conflict of interest.

## Supporting information

As a service to our authors and readers, this journal provides supporting information supplied by the authors. Such materials are peer reviewed and may be re‐organized for online delivery, but are not copy‐edited or typeset. Technical support issues arising from supporting information (other than missing files) should be addressed to the authors.

Supplementary
