# Supplementary material for: Counterion Enhanced Organocatalysis: A Novel Approach for the Asymmetric Transfer Hydrogenation of Enones
Source: ChemCatChem. 2020 Jun 15;12(14):3776–82. doi: 10.1002/cctc.202000414 (PMC7507862; doi:10.1002/cctc.202000414)
Supplement: Supplementary file 1 — Supplementary [file CCTC-12-3776-s001.pdf]

ChemCatChem

Supporting Information

Counterion Enhanced Organocatalysis: A Novel Approach for the Asymmetric Transfer Hydrogenation of Enones

Fabian Scharinger[+], Ádám Márk Pálvölgyi[+], Veronika Zeindlhofer, Michael Schnürch, Christian Schröder, and Katharina Bica-Schröder*

1. General remarks ... 3

2. Synthesis of L-amino acid derivatives ... 4

3. General synthesis of the phosphoric acids ... 10

4. Synthesis of the Hantzsch ester ... 14

5. Synthesis of ATH substrates ... 15

6. General procedure and optimization of the ATH reactions ... 18

7. Computational studies ... 32

8. NMR specta of L-amino acid derivatives ... 38

9. NMR specta of phosphoric acids and its intermediates ... 45

10. NMR specta of ATH substrates ... 54

11. NMR specta of ATH products ... 59

12. Chiral GC and HPLC traces ... 65

13. References ... 81

1. General remarks

All purchased chemicals from commercial suppliers were used without further purification. Dry solvents were pre-distilled and desiccated on aluminium oxide columns (PURESOLV, Innovative Technology).

Column chromatography was performed on standard manual glass columns using Merck (40-60 µm) silica gel with pre-distilled solvents (PE : petrolether, EtOAc : ethyl acetate, Et_2O : diethyl ether). For TLC analysis, precoated aluminium-backed plates were purchased from Merck (silica gel 60 F_{254}). UV active compounds were detected at 254 nm. Non-UV active compounds have been detected using vanillin staining solution (5% vanillin in EtOH + H_2SO_4).

^{1}H, ^{13}C, and ^{31}P NMR spectra were recorded on a Bruker Advance UltraShield 200 MHz or 400 MHz spectrometer and chemical shifts are reported in ppm using TMS (tetramethylsilane) as internal standard. Coupling constants (*J*) are given in Hz. For NMR purpose, the following abbreviations are used: s (singlet), d (doublet), t (triplet), q (quartet), m (multiplet), brs (broad singlet), dd (doublet of doublets), ddd (doublets of doublet of doublets), td (triplet of doublets), dt (doublet of triplets).

GC analysis have been performed on a Thermo Scientific Focus on BGB5 column by using FID detector. Chiral GC measurements were performed on chiral BGB columns (BGB173 or BGB175) by using FID detector.

Chiral HPLC measurements were carried out on a DIONEX UPLC equipped with a photodiode array (PDA) plus detector (190–360 nm), using a Diacel Chiralcel AS-H column (250×4.60 mm, 5 µm).

Optical rotation was measured on an Anton Paar MCP500 polarimeter at the specific conditions and the results have been compared to literature values. Concentrations are given in g / 100 ml.

HR-MS analysis was performed using HTC PAL system auto sampler, an Agilent 1100/1200 HPLC and Agilent 6230 AJS ESI-TOF mass spectrometer.

Microwave reactions were performed on a Biotage Initiator Classic in 20 ml pressure tight glass vials.

Melting points above room temperature were measured on an automated melting point system OPTI MELT of Stanford Research Systems and are uncorrected.

Infrared spectra were recorded on a Perkin-Elmer Spectrum 65 FT IR spectrometer equipped with a specac MK II Golden Gate Single Reflection ATR unit.

2. Synthesis of L-amino acid derivatives

2.1 General procedure for the synthesis of L-amino acid *tert*-butyl esters

Compound have been prepared by the following literature procedure: [1]

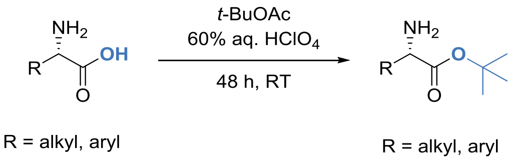

To a solution of the corresponding L-amino acid (1.0 equiv.) in *tert*-butyl acetate (18.0 equiv.), $HClO_4$ (60 % aqueous solution, 1.5 equiv.) was added dropwise at 0°C. The reaction mixture was stirred 18 h at room temperature. Distilled H_2O was added, and the reaction mixture was extracted with distilled H_2O (2×) and 1 N HCl (3×). The combined aqueous phases were then adjusted to pH 10 with a saturated Na_2CO_3 solution. The resulting solution was then extracted with CH_2Cl_2. The combined organic phases were dried over anhydrous Na_2SO_4 and concentrated under reduced pressure to give the L-amino acid *tert*-butyl esters, which were find to be pure without further purifications.

tert-Butyl L-valinate [1]

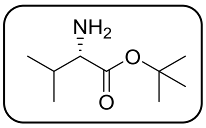

Light yellow oil (67% yield). **^{1}H NMR (400 MHz, $CDCl_3$) δ** 3.17 (d, *J* = 4.8 Hz, 1H, C*H*-NH_2), 2.03 – 1.97 (m, 1H, C*H*-$(CH_3)_2$), 1.57 (s, 2H, N*H*$_2$), 1.46 (s, 9H, 3 × C*H*$_3$-C), 0.97 (d, *J* = 6.9 Hz, 3H, C*H*$_3$-CH), 0.89 (d, *J* = 6.9 Hz, 3H, C*H*$_3$-CH).

tert-Butyl L-leucinate [1]

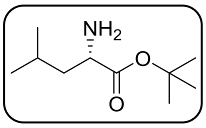

Light yellow oil (69% yield). **^{1}H NMR (400 MHz, $CDCl_3$) δ** 3.26 (t, *J* = 8.0 Hz, 1H, C*H*-NH_2), 1.74 – 1.67 (m, 1H, C*H*-$(CH_3)_2$), 1.48 – 1.37 (m, 12H, C*H*$_{2a}$-CH-NH_2, N*H*$_2$, 3 × C*H*$_3$-C), 1.33 – 1.27 (m, 1H, C*H*$_{2b}$-CH-NH_2), 0.86 (t, *J* = 9.0 Hz, 6H, 2 × C*H*$_3$-CH).

tert-Butyl L-isoleucinate [2]

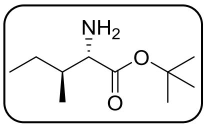

Light yellow oil (36% yield). **^{1}H NMR (400 MHz, $CDCl_3$) δ** 3.22 (d, *J* = 4.8 Hz, 1H, C*H*-NH_2), 1.73 – 1.67 (m, 1H, C*H*-CH_3), 1.48 (s, 2H, N*H*$_2$), 1.46 (s, 9H, 3 × C*H*$_3$-C), 1.12 – 1.23 (m, 2H, C*H*$_2$-CH_3), 1.12 – 0.70 (m, 6H, 2 x C*H*$_3$).

<u>*tert*-Butyl-(*S*)-2-amino-2-phenylacetate</u> [2]

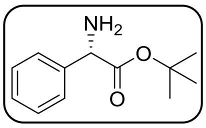

Light yellow solid (61% yield). **^{1}H NMR (400 MHz, CDCl$_3$) δ** 7.30 – 7.16 (m, 5H, *H*-arom), 4.39 (s, 1H, C*H*-NH$_2$), 1.99 (s, 2H, N*H*$_2$), 1.30 (s, 9H, 3 × C*H*$_3$-C).

<u>*tert*-Butyl L-phenylalaninate</u> [2]

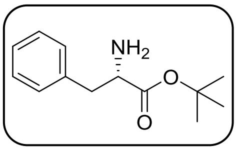

Light yellow oil (65% yield). **^{1}H NMR (400 MHz, CDCl$_3$) δ** 7.23 – 7.14 (m, 5H, *H*-arom), 3.54 (t, *J* = 7.8 Hz, 1H, C*H*-NH$_2$), 2.97 (dd, *J* = 16.0 Hz, 8.0 Hz, 1H, CH_{2a}-CH-NH$_2$), 2.77 (dd, *J* = 16.0 Hz, 8.0 Hz, 1H, CH_{2b}-CH-NH$_2$), 1.40 (s, 2H, N*H*$_2$), 1.35 (s, 9H, 3 × C*H*$_3$-C).

<u>*tert-Butyl (S)-2-amino-3,3-dimethylbutanoate*</u> [1]

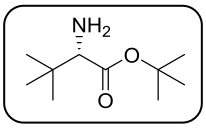

Light yellow oil (72% yield). **^{1}H NMR (400 MHz, CDCl$_3$) δ** 2.95 (s, 1H, C*H*-NH$_2$), 1.41 (s, 11H, N*H*$_2$, 3 × C*H*$_3$-C), 0.90 (s, 9H, 3 × C*H*$_3$-C).

2.2 General procedure for the synthesis of L-amino acid esters via DCC/DMAP coupling

L-Valine mentyl esters (all three diastereomers), L-valine cyclohexyl ester and L-valine 4-(*tert*-butyl)cyclohexyl ester have been prepared by the following two step general procedure: [3]

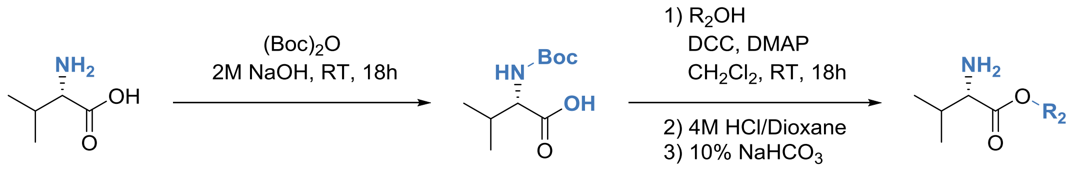

A solution of L-valine (5.0 g, 43 mmol, 1.0 equiv.) in 2 N NaOH (43 mL) was cooled to 0°C and Boc_2O (11.3 g, 51.6 mmol, 1.2 equiv.) was added slowly *via* syringe. The ice bath was removed and the reaction mixture stirred for 18 h at room temperature. The mixture was acidified (pH = 2) by using a 4 N HCl and extracted with EtOAc (3 × 50 mL). The combined organic phases were dried over anhydrous Na_2SO_4 and concentrated to give (*tert*-butoxycarbonyl)-L-valine as a colorless oil (8.3 g, 90%).

To a solution of (*tert*-butoxycarbonyl)- L-valine (1.0 equiv.) in dry CH_2Cl_2, DCC (1.1 equiv.) was added at room temperature. The suspension was cooled to 0°C followed by the addition of the corresponding alcohol (1.0 equiv.) and DMAP (0.1 equiv.). The reaction mixture was stirred for 18 h at room temperature followed by the addition of EtOAc. After 10 min of stirring, the dicyclohexyl urea was filtered off, rinsed with EtOAc and the filtrate was successively washed with 1 N HCl solution, saturated $NaHCO_3$ solution, dried over anhydrous Na_2SO_4 and concentrated under reduced pressure. 4 N HCl in dioxane was added and the resulting mixture was stirred for 3 h at room temperature. The solvent was removed, Et_2O was added and stirred for 10 min. The amino acid HCl salt was filtered off, washed with Et_2O (3×) and dried in vacuo. The salt was suspended in CH_2Cl_2, saturated $NaHCO_3$ was added and stirred for 2 h at room temperature. The organic phase was separated, washed with saturated $NaHCO_3$ (3×) and concentrated under reduced pressure to yield the corresponding esters.

<u>*Menthyl valinates (all diastereomers)*</u>

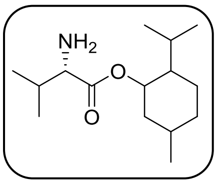

Colorless oil (DL: 58% yield, D: 55% yield, L: 58% yield). **^{1}H NMR (400 MHz, $CDCl_3$) δ** 4.73 (td, *J* = 10.9, 4.4 Hz, 1H, C*H*-O), 3.29 (d, *J* = 4.8 Hz, 1H, C*H*-NH_2), 2.15 – 2.01 (m, 1H, C*H*-$(CH_3)_2$), 2.00 – 1.01 (m, 10H, N*H_2*, *H*-menthol), 0.99 (d, *J* = 6.9 Hz, 3H, C*H_3*-CH), 0.90 (m, 9H, 2 × C*H_3*-CH, C*H_3*-CH-menthol), 0.76 (d, *J* = 7.0 Hz, 3H, C*H_3*-menthol); **^{13}C NMR (100 MHz, $CDCl_3$) δ** 175.30, 74.88, 60.40, 47.08, 41.01, 34.37, 31.90, 31.53, 26.11, 23.09, 22.15, 21.01, 19.79, 16.65, 15.89.

4-(tert-Butyl)cyclohexyl-L-valinate

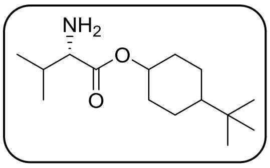

Colorless oil (18% yield). **^{1}H NMR (400 MHz, $CDCl_3$) δ** 4.73 – 4.66 (m, 1H, C*H*-O), 3.30 (d, *J* = 4.8 Hz, 1H, C*H*-NH_2), 2.25 (s, 2H, N*H*$_2$), 2.12 – 1.94 (m, 3H, C*H*-$(CH_3)_2$, C*H*$_2$-CH-O), 1.85 – 1.75 (m, 2H, C*H*$_2$-CH-O), 1.41 – 1.22 (m, 2H, C*H*$_2$-CH-C-tbu), 1.18 – 1.00 (m, 2H, C*H*$_2$-CH-C-tbu), 0.99 (d, *J* = 6.9 Hz, 3H, C*H*$_3$-CH), 0.92 (d, *J* = 6.9 Hz, 3H, C*H*$_3$-CH), 0.85 (s, 9H, 3 × C*H*$_3$-C).

Cyclohexyl L-valinate [4]

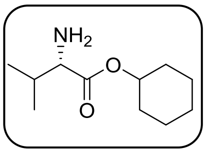

Colorless oil (49% yield). **^{1}H NMR (400 MHz, $CDCl_3$) δ** 4.93 – 4.64 (m, 1H, C*H*-O), 3.24 (d, *J* = 4.9 Hz, 1H, C*H*-NH_2), 2.05 – 1.97 (m, 1H, C*H*-$(CH_3)_2$), 1.81 (bs, 2H, N*H*$_2$), 1.74 – 1.00 (m, 10H, C*H*-cyclohexyl), 0.96 (d, *J* = 6.9 Hz, 3H, C*H*$_3$-CH), 0.88 (d, *J* = 6.9 Hz, 3H, C*H*$_3$-CH).

2.3 Synthesis of other L-amino acid esters

Methyl L-valinate [5]

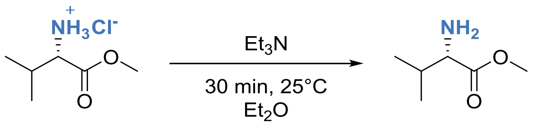

To a slurry of L-valine methyl-ester hydrochloride (1.27 g, 7.6 mmol, 1.0 equiv.) in dry MeOH (1 mL), Et_3N (1.6 mL, 12 mmol, 1.6 equiv.) was added. After 10 min of stirring, dry Et_2O (30 mL) was added, the solution was cooled to 0°C and it was stirred for another 30 min. The triethylamine hydrochloride salt was filtered off and the filtrate was concentrated to yield the product as a colorless oil (800 mg, 81%). **^{1}H NMR (400 MHz, $CDCl_3$) δ** 3.70 (s, 3H, C*H*$_3$-O), 3.28 (d, *J* = 5.1 Hz, 1H, C*H*-NH_2), 2.11 – 1.93 (m, 1H, C*H*-$(CH_3)_2$), 1.45 (s, 2H, N*H*$_2$), 0.95 (d, *J* = 6.9 Hz, 3H, C*H*$_3$-CH), 0.88 (d, *J* = 6.9 Hz, 3H, C*H*$_3$-CH).

Isopropyl L-valinate [6]

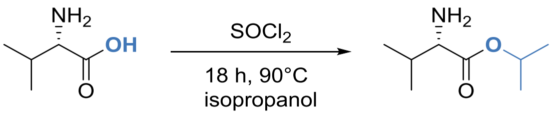

To a solution of L-valine (1.0 g, 8.5 mmol, 1.0 equiv.) in isopropanol (26 mL, 341 mmol, 40 equiv.) was added thionyl chloride (3.1 mL, 43 mmol, 5.0 equiv.) at 0°C. The reaction mixture was then refluxed for 18 h. After cooling to room temperature, the solvent was evaporated, Et_2O (50 mL) was added and the amino acid hydrochloride salt was filtered off. CH_2Cl_2 (50 mL) was added, followed by the addition of sat. $NaHCO_3$ (50 mL) and the two phases were stirred for 30 min. The organic phase was washed several times with sat. $NaHCO_3$ (3 × 25 mL), dried over anhydrous Na_2SO_4 and concentrated to give the product as a colorless liquid (380 mg, 23%). **^{1}H NMR (400 MHz, $CDCl_3$) δ** 5.09 – 5.02 (m, 1H, C*H*-O), 3.26 (d, *J* = 4.9 Hz, 1H, C*H*-NH_2), 2.07 – 1.99 (m, 1H, C*H*-$(CH_3)_2$), 1.82 (s, 2H, N*H*$_2$), 1.42 – 1.16 (m, 6H, 2 x C*H*$_3$-CH-O), 0.98 (d, *J* = 6.9 Hz, 3H, C*H*$_3$-CH), 0.91 (d, *J* = 6.9 Hz, 3H, C*H*$_3$-CH).

Benzyl L-valinate [7]

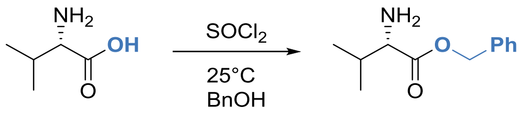

To a solution of L-valine (1.0 g, 8.5 mmol, 1.0 equiv.) in benzyl alcohol (26 mL, 341 mmol, 40 equiv.) was added thionyl chloride (3.1 mL, 43 mmol, 5.0 equiv.) at 0°C. The reaction mixture was then stirred for 18 hours at room temperature. Et_2O (50 mL) was added and the amino acid hydrochloride salt was

filtered off. CH_2Cl_2 (50 mL) was added, followed by the addition of sat. $NaHCO_3$ (50 mL) and the two phases were stirred for 30 min. The organic phase was washed several times with sat. $NaHCO_3$ (3 × 25 mL), dried over anhydrous Na_2SO_4 and concentrated under reduced pressure to give the product as a colorless liquid (1.12 g, 62%). **^{1}H NMR (400 MHz, $CDCl_3$) δ** 7.37 – 7.32 (m, 5H, *H*-arom), 5.15 (dd, *J* = 12.0 Hz, 16.0 Hz, 1H, CH_2-arom), 3.34 (d, *J* = 4.0 Hz, 1H, C*H*-NH_2), 2.08 – 2.01 (m, 1H, C*H*-$(CH_3)_2$), 1.68 (s, 2H, NH_2), 0.96 (d, *J* = 6.9 Hz, 3H, CH_3-CH), 0.88 (d, *J* = 6.9 Hz, 3H, CH_3-CH).

3. General synthesis of the phosphoric acids

The phosphoric acids have been prepared following either a one or a two-step reaction pathway:

1.) One step procedure: [8]

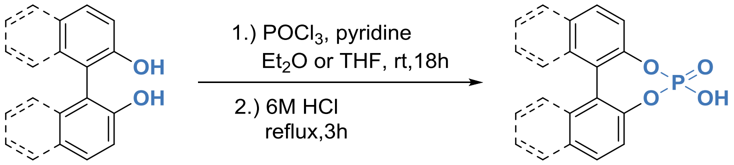

To a solution of the corresponding diol (1.0 equiv.) in dry Et_2O or THF pyridine (2.0 equiv.) was added. The reaction mixture was cooled to 0°C and $POCl_3$ (1.4 equiv.) was added slowly *via* syringe. The resulting reaction mixture was stirred for 18 h at room temperature. The pyridinium hydrochloride was filtered off and it was rinsed several times with Et_2O. The filtrate was concentrated and recrystallized from *n*-heptane. The white solid was filtered off, washed with *n*-heptane (3×) and hydrolyzed with 6 N HCl for 3 h at reflux temperature. Filtration gave the products as a white solid.

6-Hydroxydibenzo[d,f][1,3,2]dioxaphosphepine 6-oxide [8]

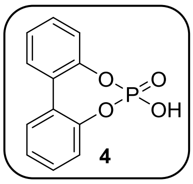

White solid (81% yield). **^{1}H NMR (400 MHz, DMSO-d$_6$) δ** 7.63 (dd, *J* = 7.6, 1.7 Hz, 2H, *H*-arom), 7.50 (td, *J* = 7.8, 1.7 Hz, 2H, *H*-arom), 7.39 (tt, *J* = 7.5, 1.3 Hz, 2H, *H*-arom), 7.32 – 7.21 (m, 2H, *H*-arom); **^{31}P NMR (162 MHz, DMSO-d$_6$)** 1.72. *Analytical data were in accordance with the literature.*[8]

4-Hydroxydinaphtho[2,1-d:1',2'-f][1,3,2]dioxaphosphepine 4-oxide

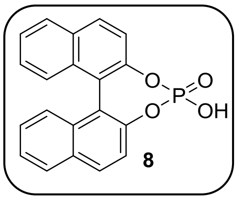

White solid ((*S*,*R*): 89% yield, (*R*): 93% yield, (*S*): 94% yield). **^{1}H NMR (400 MHz, DMSO-d$_6$) δ** 8.18 (d, *J* = 8.9 Hz, 2H, *H*-arom), 8.08 (d, *J* = 1.3 Hz, 2H, *H*-arom), 7.57 (dd, *J* = 8.8, 0.9 Hz, 2H, *H*-arom), 7.52 (ddd, *J* = 8.1, 6.7, 1.2 Hz, 2H, *H*-arom), 7.37 (ddd, *J* = 8.3, 6.8, 1.4 Hz, 2H, *H*-arom), 7.23 (dd, *J* = 8.5, 1.1 Hz, 2H, *H*-arom); **^{31}P NMR (162 MHz, DMSO-d$_6$)** 2.66. *Analytical data were in accordance with the literature.*[8]

2.) Two step procedure: [9, 10]

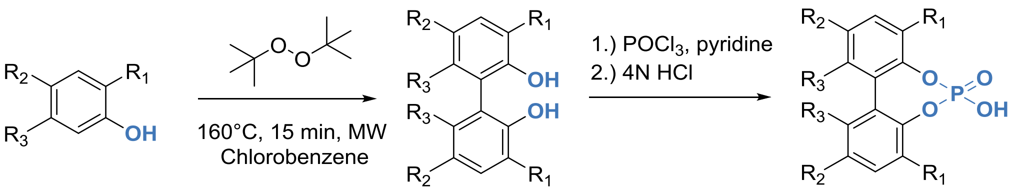

R_1 = *i*-Pr, R_2 = H, R_3 = CH_3	R_1 = *i*-Pr, R_2 = H, R_3 = CH_3	R_1 = *i*-Pr, R_2 = H, R_3 = CH_3
R_1 = *t*-Bu, R_2 = H, R_3 = CH_3	R_1 = *t*-Bu, R_2 = H, R_3 = CH_3	R_1 = *t*-Bu, R_2 = H, R_3 = CH_3
R_1 = *t*-Bu, R_2 = CH_3, R_3 = H	R_1 = *t*-Bu, R_2 = CH_3, R_3 = H	R_1 = *t*-Bu, R_2 = CH_3, R_3 = H

In a 20 mL microwave vial equipped with a stir bar, the corresponding phenol (1.0 equiv.) was dissolved in chlorobenzene (1.66 M). Di-*tert*-butyl peroxide (1.05 equiv.) was then added *via* syringe. The vial was capped and the reaction mixture was stirred for 15 min at room temperature. The vial was then placed in the microwave reactor and heated to 160°C (high absorption setting) and stirred for 15 min. The reaction mixture was allowed to cool down and volatiles have been removed under reduced pressure. The resulting crude mixture was purified by column chromatography (EtOAc: PE, UV visualization) to afford the desired 2,2'-diols (**Step A**).

To a solution of the corresponding 2,2'-diol (1.0 equiv.) in pyridine, $POCl_3$ (2.0 equiv.) was added slowly *via* syringe at 0°C. After stirring the reaction mixture for 24 h at 95°C, it was cooled to room temperature and distilled H_2O was added slowly. The resulting clear solution was stirred for another 18 h at 95 °C. After cooling down to room temperature, 4 N HCl was added slowly. The precipitate of the product was filtered off and washed with 4 N HCl. The solid product was further hydrolyzed by re-dissolving it in CH_2Cl_2 and washing several times with 4 N HCl (3-4×) After being dried over anhydrous Na_2SO_4, removal of the solvent gave the phosphoric acids (**Step B**).

*3,3'-Di-tert-butyl-5,5'-dimethyl-[1,1'-biphenyl]-2,2'-diol (**STEP A**)* [11]

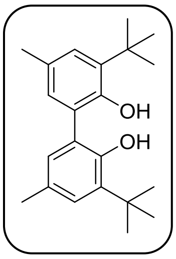

Column chromatography (1.5% EtOAc in light petrol) afforded the product as pale yellow solid (53% yield). **^{1}H NMR (400 MHz, $CDCl_3$) δ** 7.20 (d, *J* = 2.1 Hz, 2H, *H*-arom), 6.94 (d, *J* = 2.1 Hz, 2H, *H*-arom), 5.24 (s, 2H, 2 × O*H*), 2,36 (s, 6H, 2 × C*H*$_3$), 1.48 (s, 18H, 6 × C*H*$_3$-C).

3,3'-Diisopropyl-6,6'-dimethyl-[1,1'-biphenyl]-2,2'-diol ***(STEP A)*** [12]

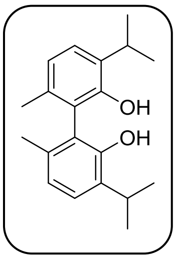

Column chromatography (2% EtOAc in light petrol) afforded the product as light yellow oil (58% yield). **^{1}H NMR (400 MHz, $CDCl_3$) δ** 7.19 (d, *J* = 7.7 Hz, 2H, *H*-arom), 6.89 (d, *J* = 7.8 Hz, 2H, *H*-arom), 4.76 (s, 2H, 2 × O*H*), 3.32 – 3.25 (m, 2H, 2 × C*H*-$(CH_3)_2$), 1.96 (s, 6H, 2 × C*H*$_3$), 1.26 (dd, *J* = 6.9, 2.7 Hz, 12H, 4 × C*H*$_3$-CH); **^{13}C NMR (100 MHz, $CDCl_3$) δ** 150.99, 135.67, 132.57, 126.75, 122.22, 119.38, 27.17, 22.64, 22.50, 19.26.

3,3'-Di-tert-butyl-6,6'-dimethyl-[1,1'-biphenyl]-2,2'-diol ***(STEP A)*** [13]

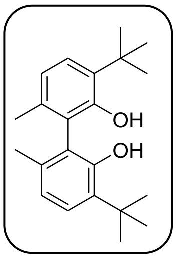

Column chromatography (1.5% EtOAc in light petrol) afforded the product as white solid (45% yield). **^{1}H NMR (400 MHz, $CDCl_3$) δ** 7.27 (d, *J* = 8.0 Hz, 2H, *H*-arom), 6.87 (d, *J* = 8.0 Hz, 2H, *H*-arom), 5.01 (s, 2H, 2 × O*H*), 1.94 (s, 6H, 2 × C*H*$_3$), 1.42 (s, 18H, 6 × C*H*$_3$-C); **^{13}C NMR (100 MHz, $CDCl_3$) δ** 152.35, 134.08, 127.50, 121.97, 120.26, 34.79, 29.64, 19.28.

4,8-Di-tert-butyl-6-hydroxy-2,10-dimethyldibenzo[d,f][1,3,2]dioxaphosphepine 6-oxide ***(STEP B)*** [14]

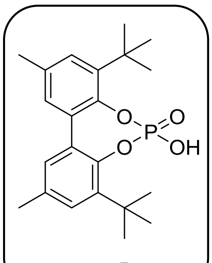

5

Grey solid (63% yield). **^{1}H NMR (400 MHz, $CDCl_3$) δ** 7.26 (s, 2H, *H*-arom), 7.02 (s, 2H, *H*-arom), 2.36 (s, 6H, 2 × C*H*$_3$), 1.50 (s, 18H, 6 × C*H*$_3$-C); **^{31}P NMR (162 MHz, $CDCl_3$) δ** 0.48. *Analytical data were in accordance with the literature.*[15]

6-Hydroxy-4,8-diisopropyl-1,11-dimethyldibenzo[d,f][1,3,2]dioxaphosphepine-6-oxide ***(STEP B)***

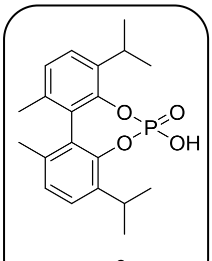

6

Brown solid (85% yield). **^{1}H NMR (400 MHz, $CDCl_3$) δ** 7.27 (d, *J* = 8.0 Hz, 2H, *H*-arom), 7.15 (d, *J* = 8.0 Hz, 2H, *H*-arom), 3.49 – 3.45 (m, 2H, 2 × C*H*-$(CH_3)_2$), 2.14 (s, 6H, 2 × C*H*$_3$), 1.27 (dd, *J* = 25.5, 6.9 Hz, 12H, 4 × C*H*$_3$-CH); **^{31}P NMR (162 MHz, $CDCl_3$) δ** 2.40; **^{13}C NMR (100 MHz, $CDCl_3$) δ** 145.40, 137.74, 136.08, 128.02, 127.11, 126.31, 26.60, 24.10, 22.75, 19.67; **IR ATR (ν_{max}/cm^{-1})** 2963 (O-H), 2872 (C-H), 1610 (C=C), 1204 (C-O), 967 (C-H), 818 (C-H arom); **HRMS (ESI-TOF)** [M + Na]$^+$ calc. 383.1388, found 383.1389.

*4,8-Di-tert-butyl-6-hydroxy-1,11-dimethyldibenzo[d,f][1,3,2]dioxaphosphepine 6-oxide (**STEP B**)*

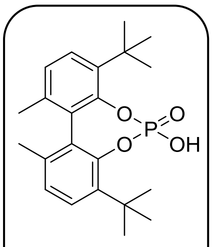

7

White solid (66% yield). **^{1}H NMR (400 MHz, $CDCl_3$) δ** 7.38 (dd, *J* = 8.0, 1.5 Hz, 2H, *H*-arom), 7.11 (dd, *J* = 11.8, 8.2 Hz, 2H, *H*-arom), 2.01 (d, *J* = 27.3 Hz, 6H, 2 × C*H*$_3$), 1.48 (d, *J* = 12.4 Hz, 18H, 6 × C*H*$_3$-C); **^{31}P NMR (162 MHz, $CDCl_3$) δ** 3.99; **^{13}C NMR (100 MHz, $CDCl_3$) δ** 144.48, 144.31, 140.81, 140.72, 134.81, 130.10, 129.91, 128.89, 35.18, 31.26, 21.10; **IR ATR ($ν_{max}$/cm^{-1})** 2961 (O-H), 2872 (C-H), 1610 (C=C), 1308 (C-O), 1023 (C-H), 901 (C-H arom); **HRMS (ESI-TOF)** [M + Na]$^+$ calc. 411.1701, found 411.1732.

4. Synthesis of the Hantzsch ester

Diethyl 2,6-dimethyl-1,4-dihydropyridine-3,5-dicarboxylate [16]

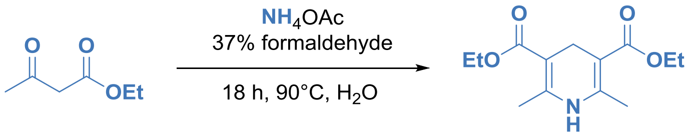

Compound was prepared by the following modified literature procedure.

A mixture of formaldehyde (37% aqueous solution, 4.0 g, 49 mmol, 1.0 equiv.), NH_4OAc (7.6 g, 99 mmol, 2.0 equiv.), ethyl acetoacetate (26 g, 0.2 mol, 4.0 equiv.) and distilled H_2O (100 mL) was refluxed at 90°C for 18 h. After cooling the reaction mixture to room temperature, cold distilled H_2O (100 mL) was added, the crude product was filtered off, washed with cold distilled H_2O (100 mL) and dried in vacuo. Recrystallization from MeOH gave the product as a yellow solid (7.5 g, 60%). **^{1}H NMR (400 MHz, $CDCl_3$) δ** 5.19 (s, 1H, N*H*), 4.16 (q, *J* = 7.1 Hz, 4H, 2 × CH_2-O), 3.26 (s, 2H, CH_2), 2.19 (s, 6H, 2 × CH_3), 1.28 (t, *J* = 7.1 Hz, 6H, 2 × CH_3-CH_2-O).

5. Synthesis of ATH substrates

3-Methyl-2-cyclohexenone, 3-methyl-cyclopentenone and isophorone have been purchased from commercial supplier (*Sigma Aldrich*).

The remaining 3-alkylcyclohexenones and 3-arylcyclohexanones have been prepared by the following procedure, starting from 3-ethoxycyclohexenone (*Acros Organics*): [17]

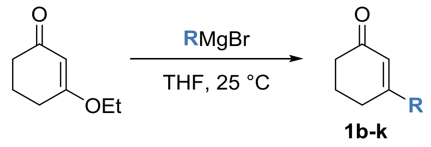

A 3 M solution of the Grignard reagent was prepared freshly from freshly ground Mg (1.0 equiv.), alkyl/aryl bromide (1.0 equiv.) in dry THF, and it was refluxed for 1 h. After being cooled to 0°C, 3-ethoxycyclohexenone (1.0 equiv.) was added slowly and the reaction mixture was stirred for 18 h at room temperature. The reaction was quenched with 1 N HCl solution (50 mL) at 0°C. Et_2O was added and the organic phase was washed with 1 N HCl solution (3 × 25 mL), sat. $NaHCO_3$ (3 × 25 mL), dried over anhydrous Na_2SO_4 and concentrated under reduced pressure. The crude products were purified by column chromatography (Et_2O: PE, UV TLC visualization) to provide the pure products.

3-Ethyl-2-cyclohexenone [18]

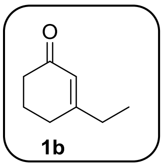

Column chromatography (25% Et_2O in PE) afforded the product as a yellow oil (67% yield). **^{1}H NMR (400 MHz, $CDCl_3$) δ** 5.87 (s, 1H, C*H*), 2.45 – 2.11 (m, 6H, C*H*$_2$-CO, C*H*$_2$-CH$_2$-CO, C*H*$_2$-C-ethyl), 2.07 – 1.81 (m, 2H, C*H*$_2$-CH$_3$), 1.09 (t, *J* = 7.4 Hz, 3H, C*H*$_3$).

3-Propyl-2-cyclohexenone [19]

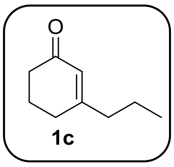

Column chromatography (25% Et_2O in PE) afforded the product as a yellow oil (60% yield). **^{1}H NMR (400 MHz, $CDCl_3$) δ** 5.87 (s, 1H, C*H*), 2.53 – 2.07 (m, 6H, C*H*$_2$-CO, C*H*$_2$-CH$_2$-CO, C*H*$_2$-C-propyl), 2.07 – 1.73 (m, 2H, C*H*$_2$-CH$_2$-CH$_3$), 1.68 – 1.36 (m, 2H, C*H*$_2$-CH$_3$), 0.93 (t, *J* = 7.4 Hz, 3H, C*H*$_3$).

3-Butyl-2-cyclohexenone [20]

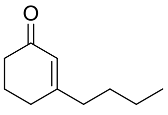

1d

Column chromatography (30% Et_2O in PE) afforded the product as a yellow oil (74% yield). **^{1}H NMR (400 MHz, $CDCl_3$) δ** 5.86 (s, 1H, C*H*), 2.48 – 2.09 (m, 6H, CH_2-CO, CH_2-CH_2-CO, CH_2-C-butyl), 2.06 – 1.79 (m, 2H, CH_2-CH_2-CH_2-CH_3), 1.61 – 1.15 (m, 4H, CH_2-CH_2-CH_3), 0.91 (t, *J* = 7.0 Hz, 3H, CH_3).

3-Isobutyl-2-cyclohexenone [21]

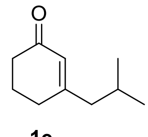

1e

Column chromatography (25% Et_2O in PE) afforded the product as a yellow oil (69% yield). **^{1}H NMR (400 MHz, $CDCl_3$) δ** 5.84 (s, 1H, C*H*), 2.54 – 2.17 (m, 4H, CH_2-CO, CH_2-CH_2-CO), 2.18 – 1.56 (m, 5H, CH_2-C-isobutyl, CH_2-$CH(CH_3)_2$, CH_2-$CH(CH_3)_2$), 0.90 (d, *J* = 6.5 Hz, 6H, 2 × CH_3-CH).

3-Isopropyl-2-cyclohexenone [22]

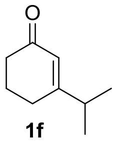

1f

Column chromatography (25% Et_2O in PE) afforded the product as a yellow oil (10% yield). **^{1}H NMR (400 MHz, $CDCl_3$) δ** 5.88 (s, 1H, C*H*), 2.61 – 2.17 (m, 5H, CH_2-CO, CH_2-CH_2-CO, CH-$(CH_3)_2$), 2.13 – 1.81 (m, 2H, CH_2-C-isopropyl), 1.10 (d, *J* = 6.9 Hz, 6H, 2 × CH_3-CH).

3-Phenyl-2-cyclohexenone [23]

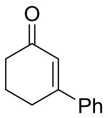

1i

Column chromatography (30% Et_2O in PE) afforded the product as a white solid (88% yield). **^{1}H NMR (400 MHz, $CDCl_3$) δ** 7.53 – 7.41 (m, 5H, *H*-arom), 6.42 (s, 1H, C*H*), 2.85 – 2.78 (m, 2H, CH_2-CO), 2.62 – 2.49 (m, 2H, CH_2-CH_2-CO), 2.18 – 2.14 (m, 2H, CH_2-C-phenyl).

3-Phenethyl-2-cyclohexenone [24]

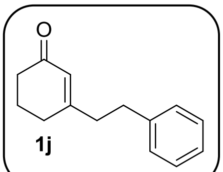

1j

The formation of the grignard-reagent and the reaction have been carried out at 40°C. Column chromatography (30% Et_2O in PE) afforded the product as a light yellow oil (91% yield). **^{1}H NMR (200 MHz, $CDCl_3$) δ** 7.34 – 7.15 (m, 5H, *H*-arom), 5.90 (s, 1H, C*H*), 2.89 – 2.76 (m, 2H, CH_2-CO), 2.58 – 2.47 (m, 2H, CH_2-CH_2-CO), 2.40 – 2.26 (m, 4H, phenyl-CH_2-CH_2) 2.05 – 1.92 (m, 2H, CH_2-C-phenyl).

3-(Naphtalen-1-yl)-2-cyclohexenone [25]

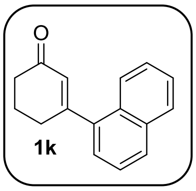

Column chromatography (30% - 50% Et_2O in PE) afforded the product as a yellow oil (94% yield). **^{1}H NMR (200 MHz, $CDCl_3$) δ** 7.92 – 7.82 (m, 3H, *H*-arom), 7.54 – 7.45 (m, 3H, *H*-arom), 7.35 – 7.28 (m, 1H, *H*-arom), 6.21 (s, 1H, C*H*), 2.82 – 2.74 (m, 2H, CH_2-CO), 2.64 – 2.56 (m, 2H, CH_2-CH_2-CO), 2.33 – 2.19 (m, 2H, CH_2-C-arom).

3-Piperonyl-2-cyclohexenone [26]

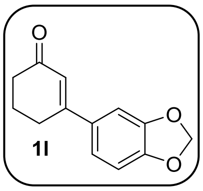

Column chromatography (30% Et_2O in PE) afforded the product as a yellow foam (90% yield). **^{1}H NMR (200 MHz, $CDCl_3$) δ** 7.06 (ddd, *J* = 8.0 Hz, 4.0 Hz, 2.0 Hz, 2H, *H*-arom), 6.83 (d, *J* = 8.0 Hz, 1H, *H*-arom), 6.33 (s, 1H, C*H*), 6.01 (s, 2H, OCH_2), 2.76 – 2.67 (t, *J* = 6.0 Hz, 2H, CH_2-CO), 2.50 – 2.41 (t, *J* = 6.0 Hz, 2H, CH_2-CH_2-CO), 2.19 – 2.06 (m, 2H, CH_2-C-arom).

6. General procedure and optimization of the ATH reactions

6.1 General procedures

General procedure for parameter optimization

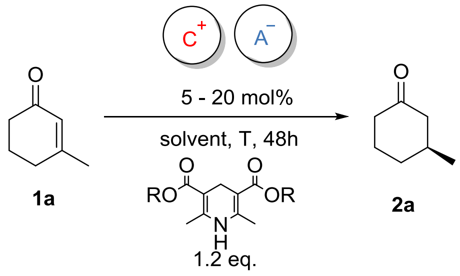

C: protonated L-amino-acid ester
A: achiral phosphate

An 8 mL screw cap vial equipped with a magnetic stir bar was charged with 3-methylcyclohexenone (0.18 mmol, 1.0 equiv.) in the appropriate solvent (0.55 mL, 0.33 M), followed by the addition of the catalyst (0.009 – 0.036 mmol, 5 mol% – 20 mol%) and the appropriate Hantzsch ester (0.22 mmol, 1.2 equiv.). The reaction mixture was stirred at the given temperature for 48 h. The conversion and yield have been determined by GC measurement by using *n*-dodecane as internal standard, while chiral GC have been used to determine the enantiomeric excess.

General procedure for scope and limitations

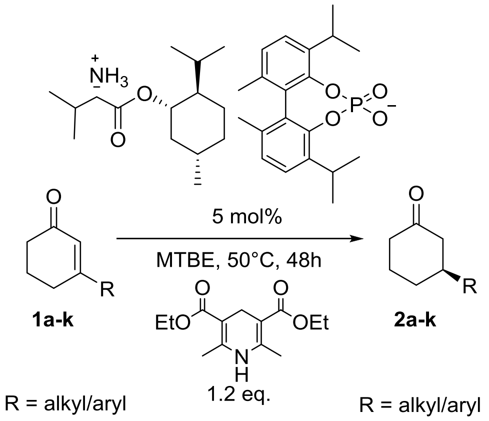

A 20 mL microwave vial equipped with a magnetic stir bar was charged with ketone (1.8 mmol, 1.0 equiv.) in MTBE (5.5 mL, 0.33 M), followed by the addition of the catalyst (55.4 mg, 0.09 mmol, 5 mol%) and Hantzsch ester (552 mg, 2.2 mmol, 1.2 equiv.). The reaction mixture was stirred at 50°C for 48 h. After cooling to room temperature, Et_2O (5 mL) and 4 M HCl (10 mL) was added and the mixture was stirred until the phases were transparent (30 min). The phases were separated and the organic phase

was washed with 4 M HCl (3 × 20 mL). The organic phase was dried over Na_2SO_4 and concentrated under reduced pressure. Purification by column chromatography (Et_2O: PE, vanillin staining agent or UV visualization) gave the 3-substituted cyclohexanones.

6.2 Analytical data of ATH products

(S)-3-Methylcyclohexanone

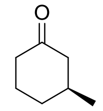
2a

Column chromatography (25% Et_2O in PE) afforded the product as a colorless oil. GC yield: 98%, isolated yield: 72% due to high volatility, 95% ee. $[\alpha]_D^{20}$**:** −11.1 (c = 1.0, $CHCl_3$); **^{1}H NMR (400 MHz, $CDCl_3$) δ** 2.41 – 2.21 (m, 3H), 2.09 – 1.82 (m, 4H), 1.76 – 1.57 (m, 1H), 1.42 – 1.24 (m, 1H), 1.02 (d, *J* = 6.4 Hz, 3H). **Determination of the enantiomeric excess** BGB175 column (85°C isotherm 20 min, 30°C/min to 220°C): t_R (*S*) = 9.0 min, t_R (*R*) = 9.6 min.

(S)-3-Ethylcyclohexanone

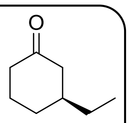
2b

Column chromatography (25% Et_2O in light petrol) afforded the product as a colorless oil. GC yield: 85%, isolated yield: 76% due to high volatility, 92% ee. $[\alpha]_D^{20}$**:** −15.6 (c = 1.0, $CHCl_3$); **^{1}H NMR (400 MHz, $CDCl_3$) δ** 2.45 – 2.23 (m, 3H), 2.09 – 1.87 (m, 3H), 1.76 – 1.63 (m, 2H), 1.41 – 1.28 (m, 3H), 0.91 (t, *J* = 7.5 Hz, 3H). **Determination of the enantiomeric excess** BGB173 column (52°C isotherm 110 min, 30°C/min to 220°C): t_R (*R*) = 51.0 min, t_R (*S*) = 54.9 min.

(S)-3-Propylcyclohexanone

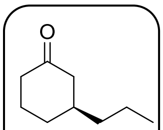
2c

Column chromatography (25% Et_2O in PE) afforded the product as a colorless oil. GC yield: 70%, isolated yield: 55% due to high volatility, 91% ee. $[\alpha]_D^{20}$**:** −14.7 (c = 1.0, $CHCl_3$); **^{1}H NMR (400 MHz, $CDCl_3$) δ** 2.42 – 2.22 (m, 3H), 2.06 – 1.86 (m, 3H), 1.80 – 1.74 (m, 1H), 1.67 – 1.59 (m, 1H), 1.35 – 1.26 (m, 5H), 0.92 – 0.86 (m, 3H). **Determination of the enantiomeric excess** BGB175 column (70°C isotherm 60 min, 1°C/min to 150 °C, 5 min at 150°C, 30°C /min to 220°C): t_R (*S*) = 50.6 min, t_R (*R*) = 53.1 min.

(S)-3-Butylcyclohexanone

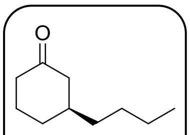

2d

Column chromatography (25% Et_2O in PE) afforded the product as a colorless oil. GC yield: 91%, isolated yield: 87%, 93% ee. $[\alpha]_D^{20}$: −15.0 (c = 1.0, $CHCl_3$); **^{1}H NMR (400 MHz, $CDCl_3$) δ** 2.44 – 1.56 (m, 7H), 1.40 – 1.09 (m, 7H), 0.91 – 0.82 (m, 4H). **Determination of the enantiomeric excess** BGB175 column (70 °C isotherm 60 min, 1°C/min to 150°C, 5 min at 150°C, 30 °C /min to 220°C): t_R (*S*) = 76.9 min, t_R (*R*) = 78.1 min.

(S)-3-Isobutylcyclohexanone

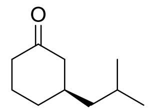

2e

Column chromatography (25% Et_2O in PE) afforded the product as a colorless oil. GC yield: 85%, isolated yield: 60% due to high volatility, 86% ee. $[\alpha]_D^{20}$: −11.3 (c = 1.0, $CHCl_3$); **^{1}H NMR (400 MHz, $CDCl_3$) δ** 2.42 – 2.21 (m, 2H), 2.03 – 1.61 (m, 4H), 1.66 – 1.41 (m, 2H), 1.33 – 1.10 (m, 3H), 0.89 – 0.84 (m, 6H). **Determination of the enantiomeric excess** BGB175 column (70 °C isotherm 5 min, 0.5 °C/min to 110°C, 5 min at 110°C, 30°C /min to 220°C): t_R (*R*) = 39.2 min, t_R (*S*) = 40.7 min.

(S)-3-Isopropylcyclohexanone

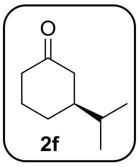

2f

Column chromatography (25% Et_2O in PE) afforded the product as a colorless oil. GC yield: 75%, isolated yield: 68% due to high volatility, 88% ee. $[\alpha]_D^{20}$: −12.4 (c = 1.0, $CHCl_3$); **^{1}H NMR (400 MHz, $CDCl_3$) δ** 2.44 – 1.23 (m, 10H), 0.92 (d, *J* = 6.0 Hz, 3H), 0.90 (d, *J* = 6.0 Hz, 3H). **Determination of the enantiomeric excess** BGB175 column (85°C isotherm 20 min, 30°C/min to 220°C): t_R (*R*) = 20.75 min, t_R (*S*) = 20.96 min.

(S)-3,5,5-Trimethylcyclohexanone

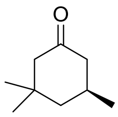

2g

Column chromatography (25% Et_2O in PE) afforded the product as a colorless oil. GC yield: 66%, isolated yield: 60% due to high volatility, 92% ee. $[\alpha]_D^{20}$: −11.9 (c = 1.0, $CHCl_3$); **^{1}H NMR (400 MHz, $CDCl_3$) δ** 2.33 – 2.14 (m, 2H), 1.92 – 1.83 (m, 3H), 1.60 – 1.56 (m, 1H), 1.38 – 1.25 (m, 2H), 1.05 – 1.01 (m, 5H), 0.88 (s, 3H). **Determination of the enantiomeric excess** BGB175 column (85°C isotherm 20 min, 30°C/min to 220°C): t_R (*S*) = 13.4 min, t_R (*R*) = 17.9 min.

(S)-3-Methylcyclopentanone

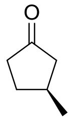

2h

Column chromatography (25% Et_2O in PE) afforded the product as a colorless oil. GC yield: 94%, isolated yield: 80% due to high volatility, 89% ee. $[\alpha]_D^{20}$**:** −13.2 (c = 1.0, $CHCl_3$); **^{1}H NMR (200 MHz, $CDCl_3$) δ** 3.22 – 2.70 (m, 1H), 2.51 – 1.81 (m, 5H), 1.32 – 1.29 (m, 1H), 1.11 – 1.02 (m, 3H). **Determination of enantiomeric excess** BGB175 column (85°C isotherm 20 min, 30°C/min to 220°C): t_R (*S*) = 8.1 min, t_R (*R*) = 8.5 min.

(S)-3-Phenylcyclohexanone

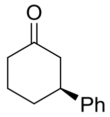

2i

Column chromatography (25% Et_2O in light petrol) afforded the product as a colorless oil. GC yield: 86%, isolated yield: 84%, 82% ee. $[\alpha]_D^{20}$**:** −17.2 (c = 1.0, $CHCl_3$); **^{1}H NMR (200 MHz, $CDCl_3$) δ** 7.39 – 7.37 (m, 2H), 7.32 – 7.27 (m, 3H), 3.11 – 3.00 (m, 1H), 2.68 – 2.44 (m, 4H), 2.23 – 2.15 (m, 2H), 1.93 – 1.81 (m, 2H). **Determination of the enantiomeric excess** BGB175 column (120°C isotherm 55 min, 30°C /min to 220°C): t_R (*R*) = 48.8 min, t_R (*S*) = 49.5 min.

(S)-3-Phenethylcyclohexanone

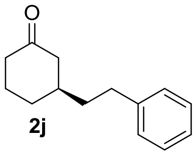

2j

Column chromatography (25% Et_2O in light petrol) afforded the product as a colorless oil. GC yield: 94%, isolated yield: 91%, 90% ee. **^{1}H NMR (200 MHz, $CDCl_3$) δ** 7.37 – 7.22 (m, 5H), 2.75 – 2.33 (m, 5H), 2.14 – 1.70 (m, 7H), 1.49 – 1.38 (m, 1H). **Determination of the enantiomeric excess** Chiralcel Diacel AS-H column (*n*-heptane/*i*-PrOH 97/3 V/V%, 1 mL/min, 25˚C, λ = 220 nm): t_R (*R*) = 15.6 min, t_R (*S*) = 16.6 min.

(S)-3-(Naphtalen-1-yl)cyclohexanone

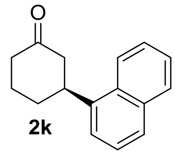

2k

Column chromatography (25% Et_2O in light petrol) afforded the product as a colorless oil. GC yield: 65%, isolated yield: 62%, 94% ee. $[\alpha]_D^{20}$**:** −49.2 (c = 1.0, $CHCl_3$); **^{1}H NMR (200 MHz, $CDCl_3$) δ** 8.05 – 7.75 (m, 3H), 7.54 – 7.38 (m, 4H), 3.92 – 3.82 (m, 1H), 2.75 – 1.94 (m, 8H). **Determination of the enantiomeric excess** Chiracel Diacel AS-H column (*n*-heptane/*i*-PrOH 95/5 V/V%, 1mL/min, 25˚C, λ = 220 nm): t_R (*R*) = 13.8 min, t_R (*S*) = 22.7 min.

(S)-3-Piperonycyclohexanone

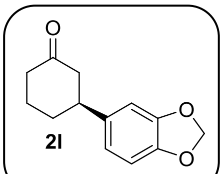

2l

Column chromatography (25% Et_2O in light petrol) afforded the product as a colorless oil. GC yield: 65%, isolated yield: 62%, 94% ee. $[\boldsymbol{\alpha}]_{\mathbf{D}}^{\mathbf{20}}$**:** -15.3 (c = 1, $CHCl_3$); **^{1}H NMR (200 MHz, $CDCl_3$) δ** 6.74 – 6.63 (m, 3H), 5.93 (s, 2H), 2.98 – 2.87 (m, 2H), 2.54 – 2.39 (m, 4H), 2.17 – 2.02 (m, 2H), 1.83 – 1.73 (m, 2H). **Determination of enantiomeric excess** Chiralcel Diacel AS-H column (*n*-heptane/*i*-PrOH 85/15 V/V%, 1 mL/min, 25˚C, λ = 220 nm): t_R (*S*) = 23.5 min, t_R (*R*) = 28.0 min.

6.3 Optimization of reaction conditions

Table S1.: Proof of concept in 1,4-dioxane

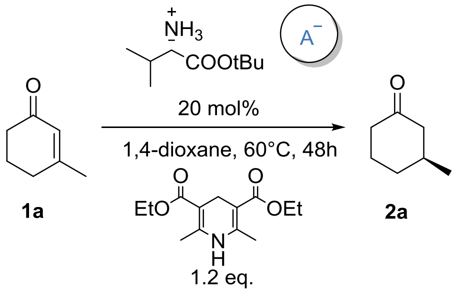

Entry[a]	Counteranion	Yield[b] [%]	ee[c] [%]	Δee[d] [%]
1	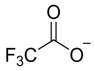	49	54 (*S*)	-
2	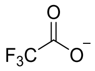	49	54 (*S*)	-
**3**	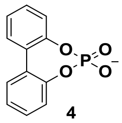 **4**	**27**	**64 (*S*)**	**+ 10**
**4[e]**	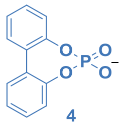 **4**	**72**	**74 (*S*)**	**+ 23**
5	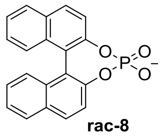 **rac-8**	13	54 (*S*)	0
6	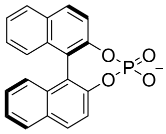 **(*R*)-8**	13	54 (*S*)	0
7	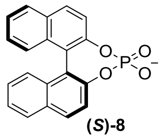 **(*S*)-8**	15	54 (*S*)	0

[a] Performed with 0.18 mmol 3-methyl-2-cyclohexenone, 0.036 mmol catalyst, 0.22 mmol Hantzsch ethyl ester in 0.55 mL 1,4-dioxane at 60°C for 48 hours. [b] Determined by GC analysis using *n*-dodecane as internal standard. [c] Determined by chiral GC analysis using a BGB175 chiral capillary column. Absolute configurations have been determined by measuring the optical rotation and comparing with literature data. [d] ee difference compare to the reaction with TFA counteranion. [e] MTBE was used as solvent.

Table S2.: Solvent screening

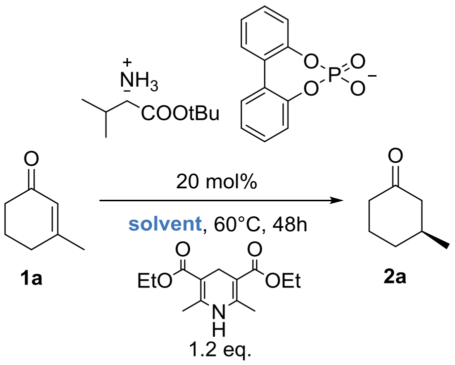

Entry[a]	Solvent	Yield[b] [%]	ee[c] [%]
1	EtOAc	48	67 (*S*)
2	BuOAc	41	62 (*S*)
3	THF	60	68 (*S*)
4	1,4-Dioxane	27	64 (*S*)
**5**	**MTBE**	**72**	**74 (*S*)**
6	Bu_2O	16	62 (*S*)
7	Toluene	17	75 (*S*)
8	$CHCl_3$	23	53 (*S*)
9	H_2O	9	42 (*S*)
10	MeOH	10	30 (*S*)
11	EtOH	20	50 (*S*)
12	^{i}PrOH	26	57 (*S*)
13	^{t}BuOH	30	60 (*S*)
14	Acetonitrile	17	37 (*S*)

[a] Performed with 0.18 mmol 3-methyl-2-cyclohexenone, 0.036 mmol catalyst, 0.22 mmol Hantzsch ethyl ester in 0.55 mL solvent at 60°C for 48 hours. [b] Determined by GC analysis using *n*-dodecane as internal standard. [c] Determined by chiral GC analysis using a BGB175 chiral capillary column. Absolute configurations have been determined by measuring the optical rotation and comparing with literature data.

Table S3.: Phosphoric acid screening

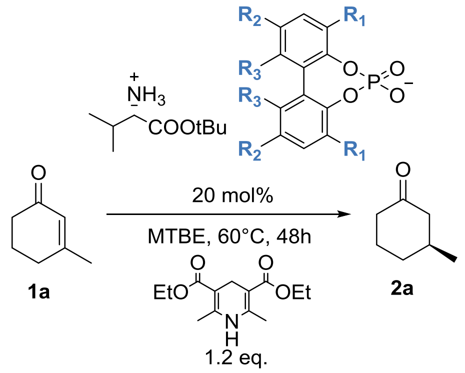

Entry[a]	Phosphoric acid	Yield[b] [%]	ee[c] [%]
1	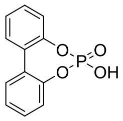 **4**	72	74 (*S*)
2	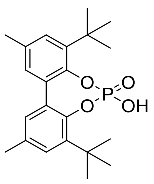 **5**	70	77 (*S*)
**3**	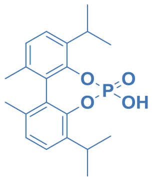 **6**	**94**	**80 (*S*)**
4	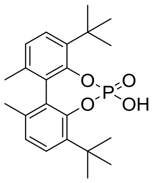 **7**	43	76 (*S*)

[a] Performed with 0.18 mmol 3-methyl-2-cyclohexenone, 0.036 mmol catalyst, 0.22 mmol Hantzsch ethyl ester in 0.55 mL MTBE at 60°C for 48 hours. [b] Determined by GC analysis using *n*-dodecane as internal standard. [c] Determined by chiral GC analysis using a BGB175 chiral capillary column. Absolute configurations have been determined by measuring the optical rotation and comparing with literature data.

Table S4.: Comparison of selectivity by screening other acids

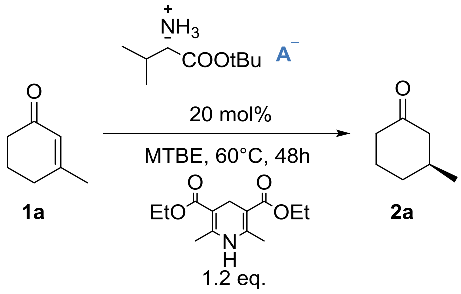

Entry[a]	Acid	Yield[b] [%]	ee[c] [%]
1	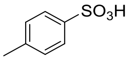	34	58 (*S*)
2	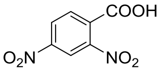	91	37 (*S*)
3	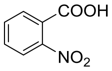	93	45 (*S*)
4	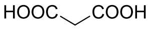	45	55 (*S*)
5	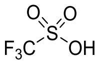	40	52 (*S*)
6	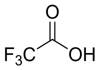	88	51 (*S*)
**7**	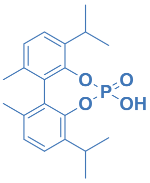 **6**	**94**	**80 (*S*)**

[a] Performed with 0.18 mmol 3-methyl-2-cyclohexenone, 0.036 mmol catalyst, 0.22 mmol Hantzsch ethyl ester in 0.55 mL MTBE at 60°C for 48 hours. [b] Determined by GC analysis using *n*-dodecane as internal standard. [c] Determined by chiral GC analysis using a BGB175 chiral capillary column. Absolute configurations have been determined by measuring the optical rotation and comparing with literature data.

Table S5: L-Amino acid screening

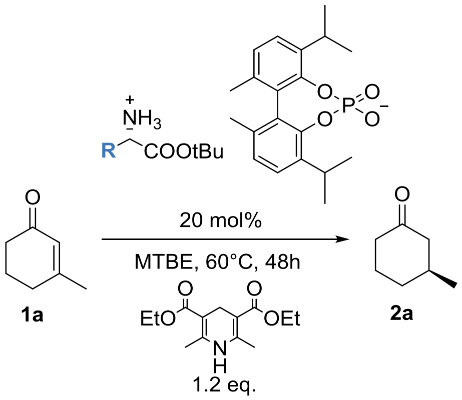

Entry[a]	L-amino acid	Yield[b] [%]	ee[c] [%]
1	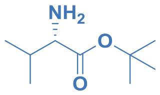	94	80 (*S*)
2	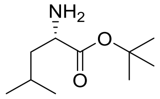	52	77 (*S*)
3	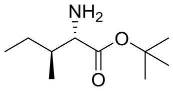	73	72 (*S*)
4	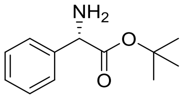	55	9 (*S*)
5	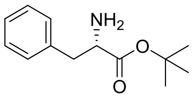	83	60 (*S*)
6	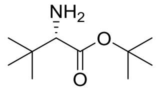	91	76 (*S*)
7	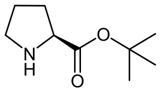	73	33 (S)

[a] Performed with 0.18 mmol 3-methyl-2-cyclohexenone, 0.036 mmol catalyst, 0.22 mmol Hantzsch ethyl ester in 0.55 mL MTBE at 60°C for 48 hours. [b] Determined by GC analysis using *n*-dodecane as internal standard. [c] Determined by chiral GC analysis using a BGB175 chiral capillary column. Absolute configurations have been determined by measuring the optical rotation and comparing with literature data.

Table S6: Ester screening

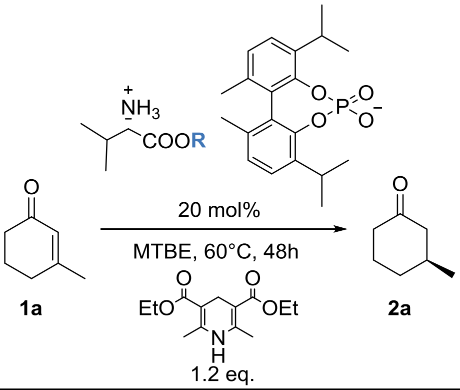

Entry[a]	L-valine ester	Yield[b] [%]	ee[c] [%]
1	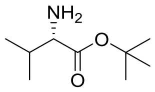	94	80 (*S*)
2	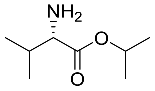	90	76 (*S*)
3	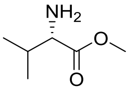	70	57 (*S*)
4	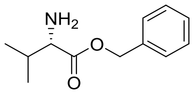	95	50 (*S*)
**5**	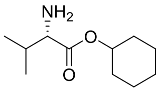	92	74 (*S*)
6	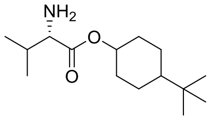	94	73 (*S*)
7	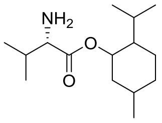	98	85 (*S*)
**8**	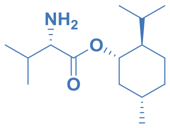	**98**	**93 (*S*)**
9	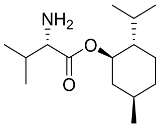	98	82 (*S*)

[a] Performed with 0.18 mmol 3-methyl-2-cyclohexenone, 0.036 mmol catalyst, 0.22 mmol Hantzsch ethyl ester in 0.55 mL MTBE at 60°C for 48 hours. [b] Determined by GC analysis using *n*-dodecane as internal standard. [c] Determined by chiral GC using a BGB175 chiral capillary column. Absolute configurations have been determined by measuring the optical rotation and comparing with literature data.

Table S7: Screening of catalyst loading and temperature

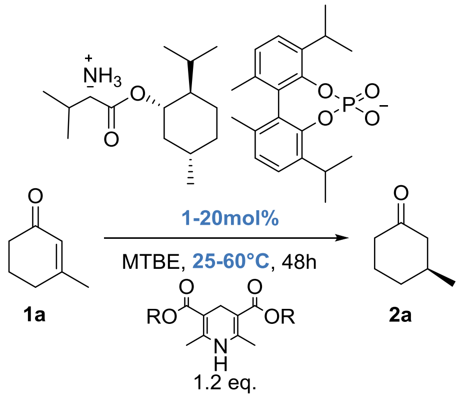

Entry[a]	Temperature	Catalyst loading [mol%]	Hantzsch ester	Yield[b] [%]	ee[c] [%]
1	60	20	ethyl	98	93 (*S*)
2	50	20	ethyl	98	95 (*S*)
3	40	20	ethyl	96	95 (*S*)
4	25	20	ethyl	59	96 (*S*)
5	25	10	ethyl	43	96 (*S*)
6	25	5	ethyl	29	96 (*S*)
7	25	1	ethyl	11	96 (*S*)
8	50	10	ethyl	99	94 (*S*)
**9**	**50**	**5**	**ethyl**	**98**	**95 (*S*)**
10	50	1	ethyl	68	93 (*S*)
11[d]	25	20	methyl	14	83 (*S*)
12[d]	25	20	ethyl	17	88 (*S*)
13[d]	25	20	*i*-propyl	58	69 (*S*)
14[d]	25	20	*t*-butyl	58	67 (*S*)

[a] Performed with 0.18 mmol 3-methyl-2-cyclohexenone, 0.009-0.036 mmol catalyst, 0.22 mmol Hantzsch ester in 0.55 mL MTBE at the given temperature for 48 hours. [b] Determined by GC analysis using *n*-dodecane as internal standard. [c] Determined by chiral GC analysis using a BGB175 chiral capillary column. Absolute configurations have been determined by measuring the optical rotation and comparing with literature data. [d] Performed with (L)-valine *t*-butyl ester instead of (L)-valine (+)-(1*R*,3*R*,4*S*) menthyl ester.

6.4 Reaction scope and limitations

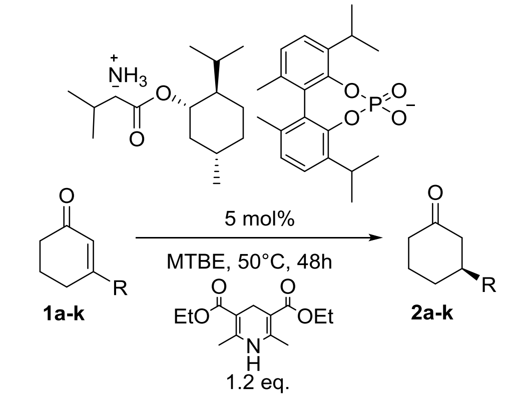

Entry[a]	Substrate	Yield[d] [%]	ee[e] [%]
1	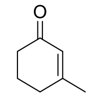 1a	98 (72)[f]	95 (*S*)
2	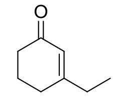 1b	85 (76)[f]	92 (*S*)
3	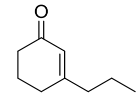 1c	70 (55)[f]	91 (*S*)
4	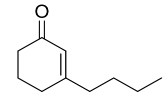 1d	91 (87)	93 (*S*)
5	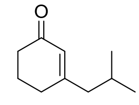 1e	85 (60)[f]	86 (*S*)
6	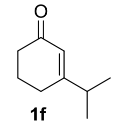 1f	75 (68)[f]	88 (*S*)
7[b]	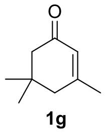 1g	66 (60)	92 (*S*)
8[c]	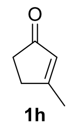 1h	94 (80)[f]	89 (*S*)
9	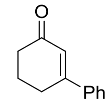 1i	86 (84)	82 (*S*)

10	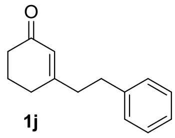 **1j**	94 (91)	90 (*S*)
11	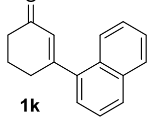 **1k**	65 (62)	94 (*S*)
12	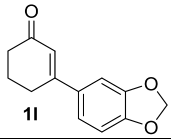 **1l**	89 (86)	70 (*S*)

[a] Performed with 1.8 mmol ketone, 5 mol% catalyst and 2.2 mmol Hantzsch ethyl ester in 5.5 mL MTBE at 50°C for 48 h. [b] 20 mol% catalyst. [c] 10 mol% catalyst. [d] Determined by GC or GC-MS analysis. Isolated yields after flash column chromatography are given in parenthesis. [e] Determined by chiral GC analysis using a BGB175 or BGB173 chiral capillary column, or by chiral HPLC analysis using a Diacel Chiralcel AS-H column. Absolute configurations have been determined by measuring the optical rotation and comparing with literature data. [f] Lower isolated yield because of high volatility of the product.

7. Computational studies

7.1 Quantummechanical calculation of rotational barriers

7.1.1 Choice of method and basis set

Although several studies on the calculation of rotational barriers in substituted biphenyls have been published[27,28,29], the only systematic protocol so far has been established by Masson.[30] A key finding of Masson's study is that for accurate barrier energies, inclusion of vibrational corrections is crucial. Since frequency computations as well as transition state optimizations for molecules as large as the substituted phosphoric acids can become quite expensive, a compromise between accuracy and efficiency is necessary in terms of the method.

The popular as well as efficient B3LYP functional[31] has been shown to perform well for the estimation of rotational barriers for unsubstituted and substituted biphenyls[29,30,32].However, as also noted by Masson, empirical dispersion correction terms such as Grimme's DFT-D or DFT-D3[33,34] are necessary to obtain accurate energies with B3LYP, so we chose B3LYP-D3 as a functional. The correlation of the uniform electron gas in the B3LYP functional was modeled according to the Vosko-Wilk-Nusair VWN5 formalism.[35]

Since Masson achieved good results with DFT/def2-TZVPP[36], we tested both the single-polarized def2-TZVP and double-polarized def2-TZVPP set for compound **4**. Since energies on the B3LYP-D3 level of theory differed only by 0.07 kcal/mol between both basis sets, we chose the computationally cheaper def2-TZVP basis set. Summarizing, all geometry optimizations, energy evaluations and frequency calculations (at 333.15K) were performed on the B3LYP-D3/def2-TZVP level of theory. All computations were performed with ORCA.[37] For further validation of the method, we compared the B3LYP-D3/def2-TZVP electronic barrier energies of phosphoric acid **4** against single point electronic energies on the same geometry computed on the benchmark DLPNO-CCSD(T)/def2-QZVP[36,38] level of theory. Only a small energy difference of 0.28 kcal/mol could be observed.

Masson also addresses the role of solvent when computing rotational barriers. In all studies on the computation of biphenyl racemization barriers published so far, good agreement with experiment is achieved without the consideration of solvent effects. Possible justification can be found in a study by Graybill and Leffler[39], who found a compensation of enthalpy and entropy in different solvents. For a more detailed discussion, see References 30 and 39.

However, Masson emphasizes that solvent effects cannot be confidently neglected for negatively charged biphenyls. Since solvent effects and likely specific interactions with both solvent and cation

need to be considered when computing rotational barriers of the anionic forms of phosphoric acids **4**-**7**, the barrier of the corresponding anions will be the topic of more detailed future work.

7.1.2 Optimization of transition states

The systematic search for rotational transition states is not straightforward. Usually, fixing the torsional dihedral angle around the aryl-aryl bond to 0 degrees and optimizing this constrained structure yields good guesses for rotational transition states of substituted biphenyl.[30] The situation is trickier when the planar transition state is hindered by bulky ortho-substituents such as the isopropyl and tert-butyl groups in phosphoric acids **6** and **7**. For all phosphoric acids, we optimized the structure while fixing one of the dihedral angles to 0° and used the result as guesses for the transition state optimizations. In addition, for phosphoric acid **6** we performed a systematic transition state search with the nudged elastic band (NEB) method implemented in ORCA.[40,41] We also used the result of this NEB calculation to construct another transition state guess for phosphoric acid **7**. The imaginary frequencies of all transition states corresponded to an out-of-plane torsion of the phenyl rings.

For phosphoric acids **4** and **5**, fully planar transition states as depicted in Figure S1 are obtained when using the constrained structures as transition state guesses.

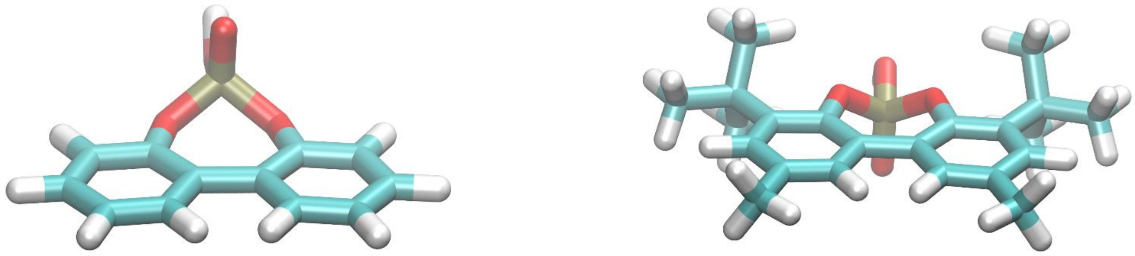

*Figure S1: Rotational transition states for phosphoric acids **4** and **5**.*

As already mentioned, compounds **6** and **7** have methyl groups in the 6,6'-position, which hinder the planar transition state. Instead, they adopt a conformation in which the aryl-aryl-bond is out-of-plane and the two rings roughly coplanar. This peculiar transition state geometry for ortho-substituted biphenyls has been anticipated by Baddeley as early as 1946.[42] Ling and Harris proposed the associated mechanism in 1964.[43] They suggested an asynchronous nature of the racemization, with 6,6'-groups passing over each other subsequently instead of synchronously, minimizing the barrier energy. The out-of-plane transition states as well as the asynchronous nature of the racemization has been confirmed by Masson's calculation.[30] The asynchronous mechanism is also visible in the case of both phosphoric acids **6** and **7**. By constraining different dihedral angles during pre-optimization, two different transition states can be obtained. The first transition state corresponds to passing of the methyl groups prior to

reaching the transition state, in the second transition state the ester oxygens of the phosphate groups have passed instead (Figure S2 and S3, left and right). No pathway is favored over the other, the electronic barriers of the two transition states for both phosphoric acid **6** and **7** only differ by 0.01 kcal/mol.

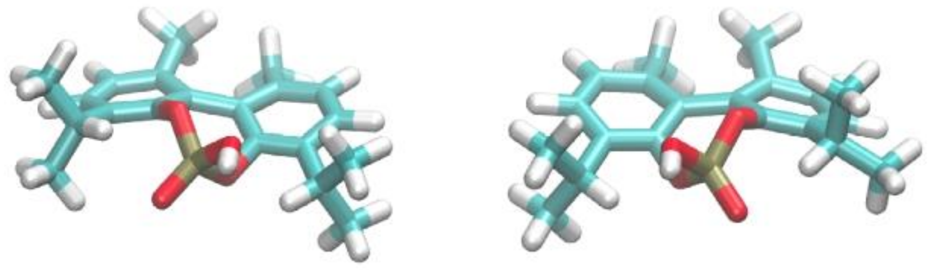

*Figure S2: Asynchronous transition states for phosphoric acid **6**.*

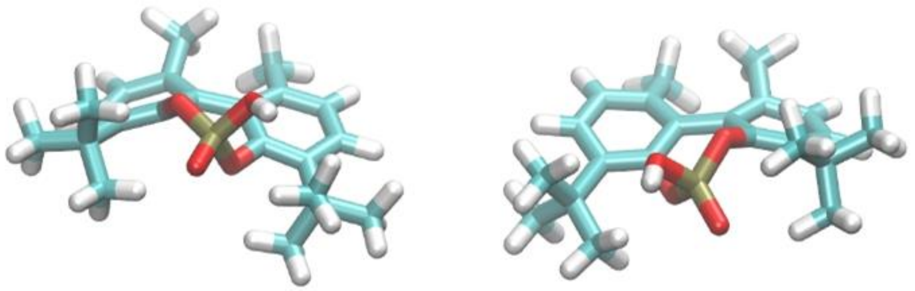

*Figure S3: Asynchronous transition states for phosphoric acid **7**.*

7.2 Molecular Dynamics Simulations

7.2.1 Simulation setup

Force fields for the anionic form of anion **6** and the iminium intermediate evolving from the L-valine (+)-(1*S*,2*R*,5*S*)-menthyl-cation were obtained from PARAMCHEM[44,45] without further modification. Since no force field for MTBE was available, we chose n-methylbutylether as a solvent. The solvent parameters were taken from the CGenFF force field[46]. Simulation boxes contained a single ion pair and 216 molecules of n-methylbutylether. To consider all possible isomers and stereisomers, both the (*E)*- and (*Z)*-form of the cation as well as both anion enantiomers were considered, yielding four different systems (see Table S8.). 10 replica simulations of every system were conducted under the following conditions: All simulations were performed with the program package CHARMM.[47] Initial configurations were created with PACKMOL.[48] All simulations were conducted in the NpT ensemble

and employed the CHARMM CPT algorithm, the leapfrog integrator and the Hoover thermostat. A temperature of 330 K, a pressure of 1 atm and a non-bonded cutoff of 11 Å were used. Electrostatics were treated using the Particle Mesh Ewald method, with a grid size of approximately 1 Å, cubic splines of order 6 and a K of 0.41 $Å^{-1}$. The SHAKE algorithm was employed to fix the length of bonds to hydrogens.
Boxes were first equilibrated for 2 ns using a timestep of 1 fs, followed by production simulations of at least 38 ns (see Table S8 for system details) under the same conditions. Coordinates were written to disk every 250 steps.

Table S8: Overview over all four simulated systems.

System number	Cation-Isomer	Anion-Isomer	Average boxsize [A]	Number of replica	Simulation time per replica [ns]
1	Z	R	36.1	10	38
2	Z	S	36.1	10	58
3	E	R	36.1	10	38
4	E	S	36.1	10	78

For trajectory analysis, the MDAnalysis library[49] as well as self-written python code was used. Plots were created with R (ggplot).[50] Hydrogen bonds were analyzed using CHARMM and a cutoff distance of 2.4 Å and a cutoff angle of 135°. Every 10th step of the trajectory was considered in the analysis.

7.2.2 Statistical considerations

To ensure statistical convergence of the simulations, several measures were taken. For both cation isomers (E and Z) 10 different conformers opimized for RMSD diversity were generated using a genetic algorithm implemented in OpenBabel. [51] Sampling of anion conformations was not done due to the structural rigidity of the anion. For each of the 10 replica/systems, a different cation conformer was used to create an initial configuration. Since we were especially interested in ion-ion-interactions, we monitored the distribution of several atom-atom interionic distances to assess sampling. Every system was simulated until the distributions reached convergence, the respective times for every system are listed in Table S8. These converged distributions are depicted in Figure S4 and show that especially the N23-O48 and N23-O49 distance distributions show full overlap.

To illustrate the distribution prior to convergence, the distributions of the E/S system after 38 ns and the full simulation time of 78 ns are depicted in Figure S5. It can be clearly seen that the N23-O48 and

N23-O49 distance distributions are different at 38 ns, but after a simulation time of 78 ns the distributions have equalized.

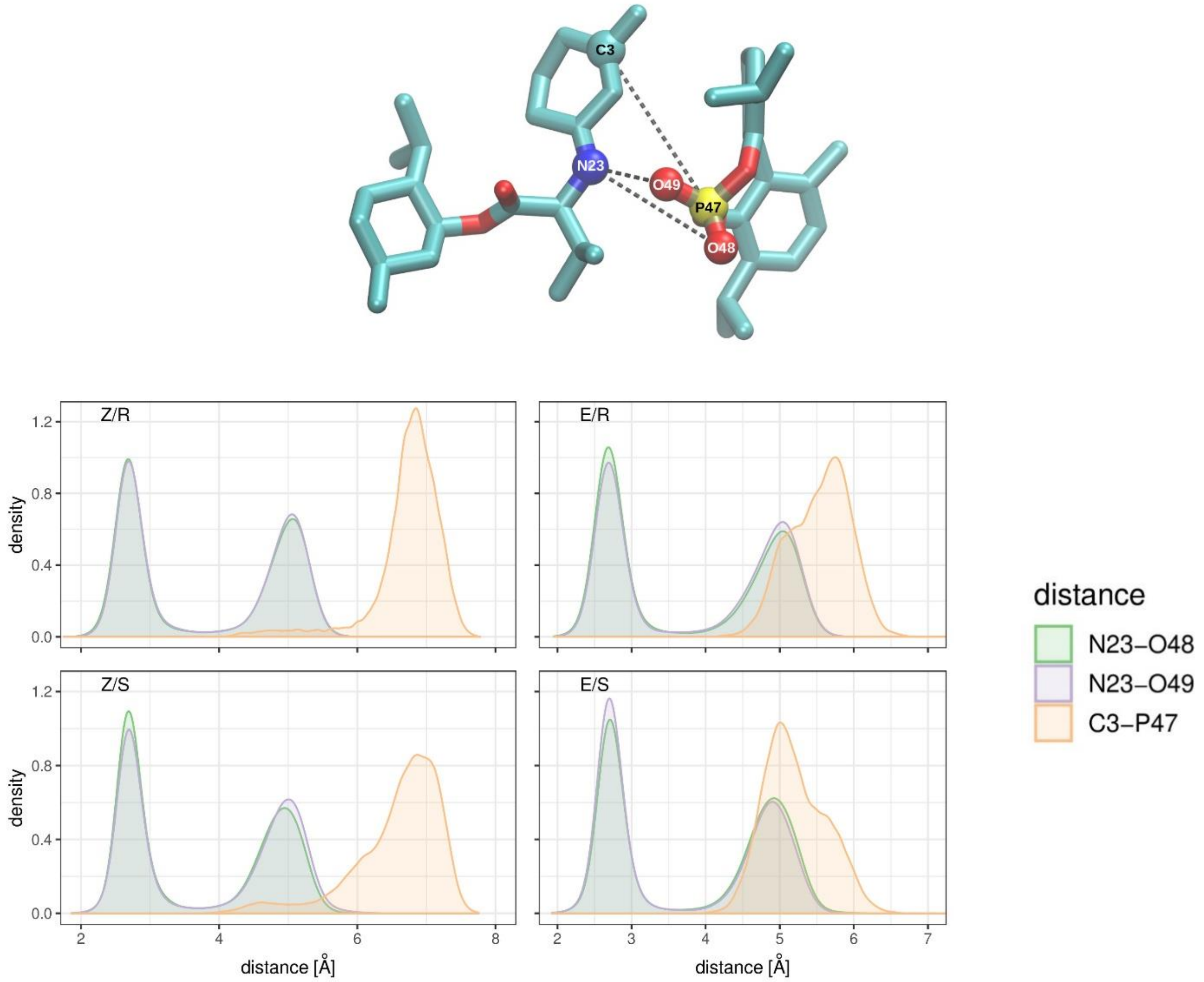

Figure S4. Interionic distances illustrated for the E-cation/S-anion pair (top) and the distributions after the full simulation time (bottom). Simulation times for the different systems are listed in Table S8.

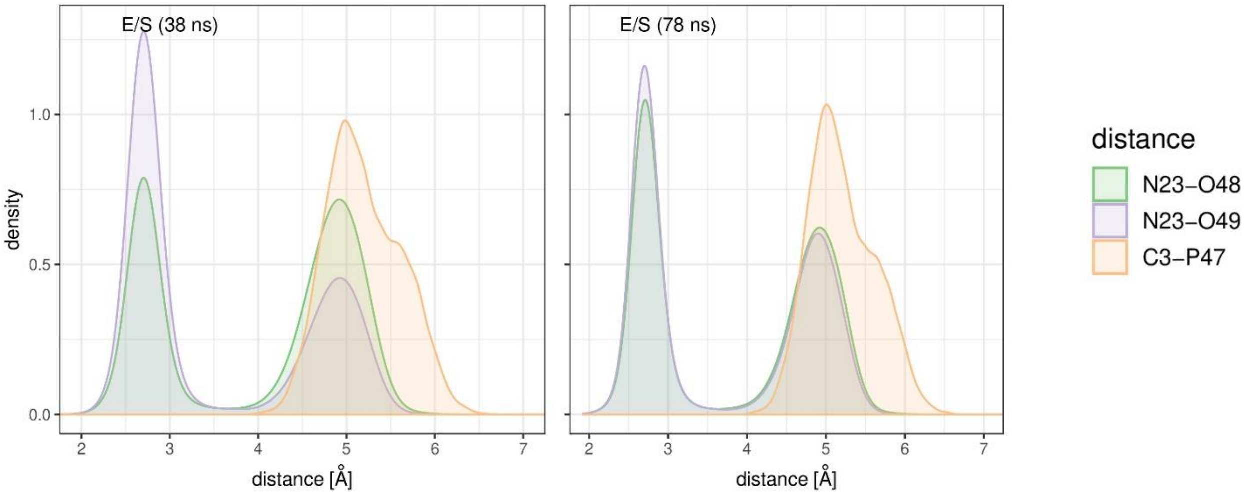

Figure S5. Distribution of interionic distances for the E-cation/S-anion pair after 38 ns (left) and 78 ns (right) simulation time. It is clearly visible that 78 ns are necessary to converge the distributions.

8. NMR specta of L-amino acid derivatives

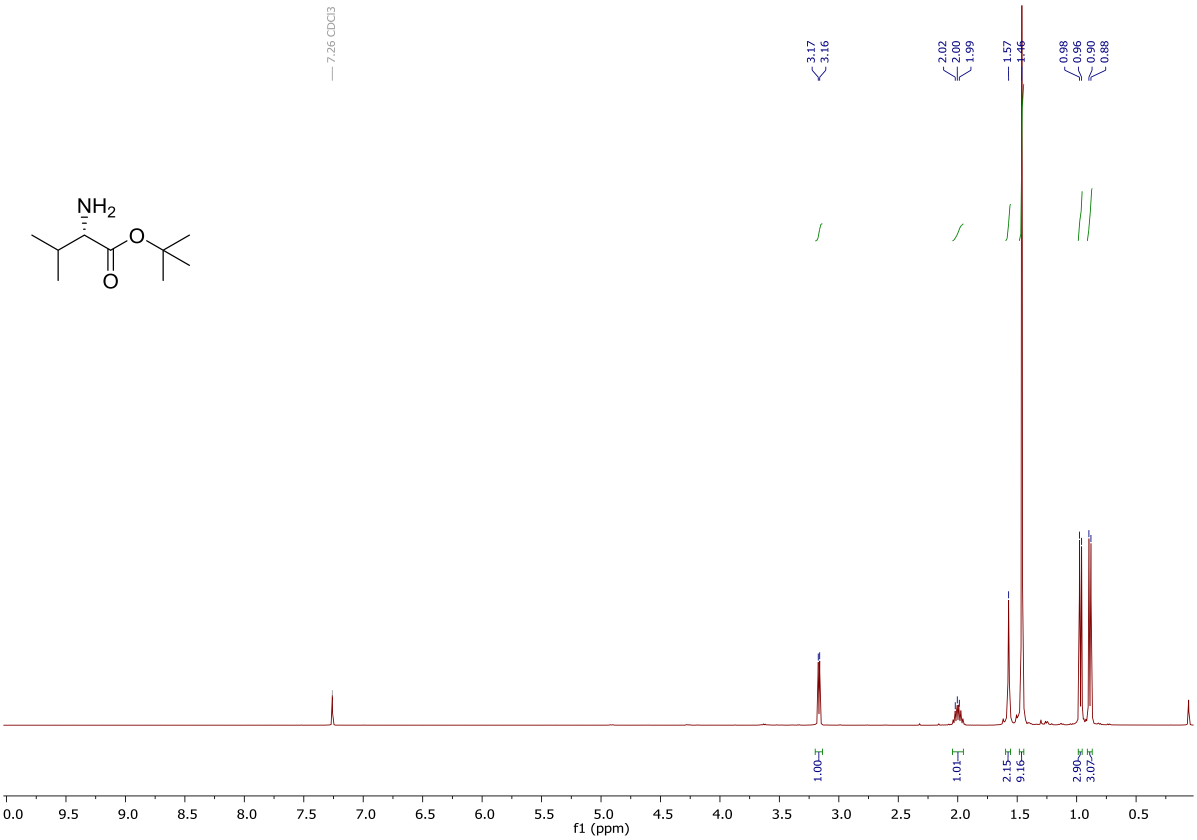

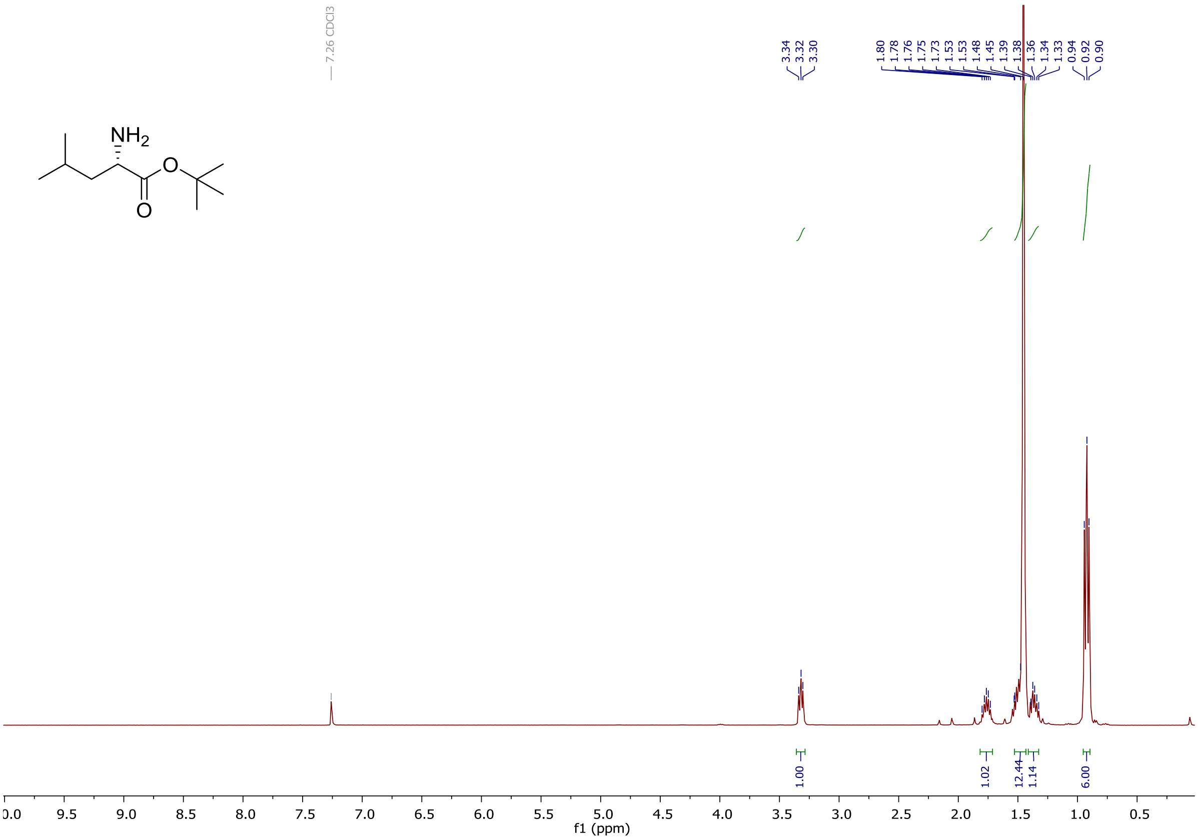

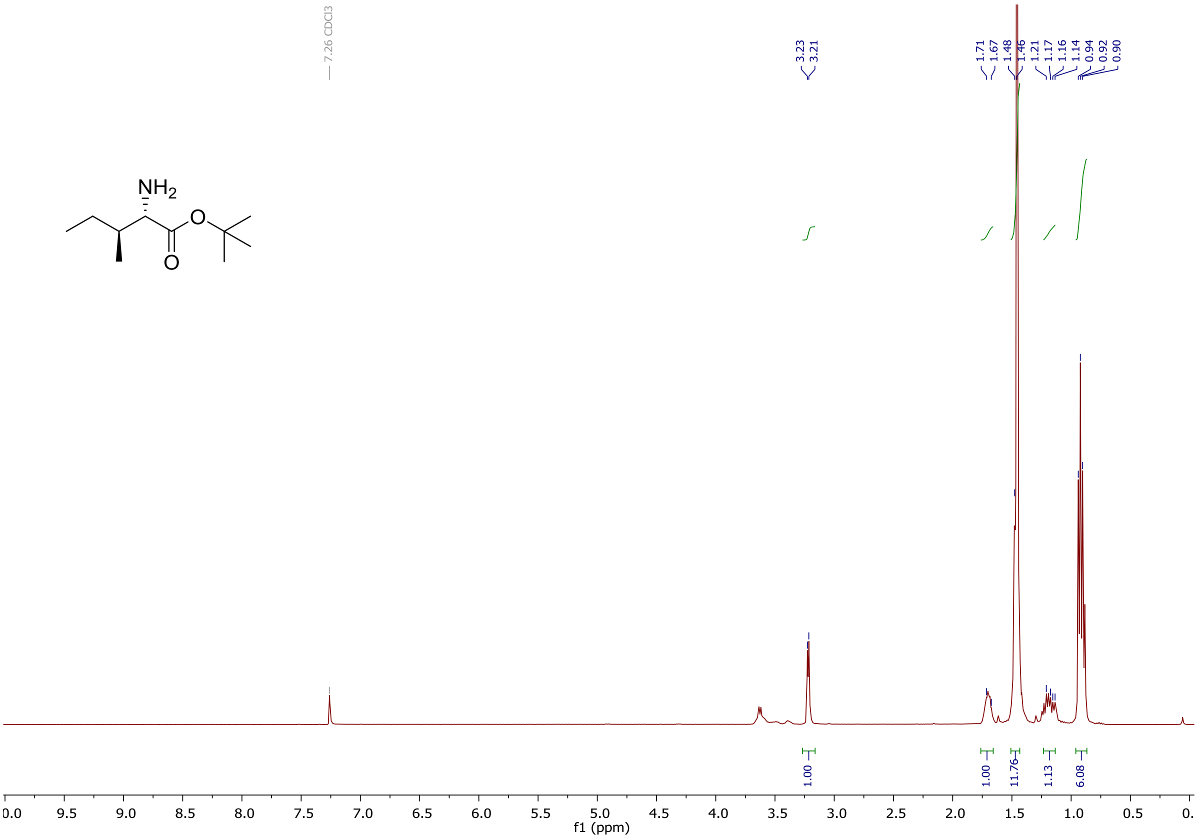
7.26 CDCl3
3.23
3.21
1.71
1.67
1.48
1.46
1.21
1.17
1.16
1.14
0.94
0.92
0.90
NH2
O
O
1.00
1.00
11.76
1.13
6.08
0.0 9.5 9.0 8.5 8.0 7.5 7.0 6.5 6.0 5.5 5.0 4.5 4.0 3.5 3.0 2.5 2.0 1.5 1.0 0.5 0.
f1 (ppm)

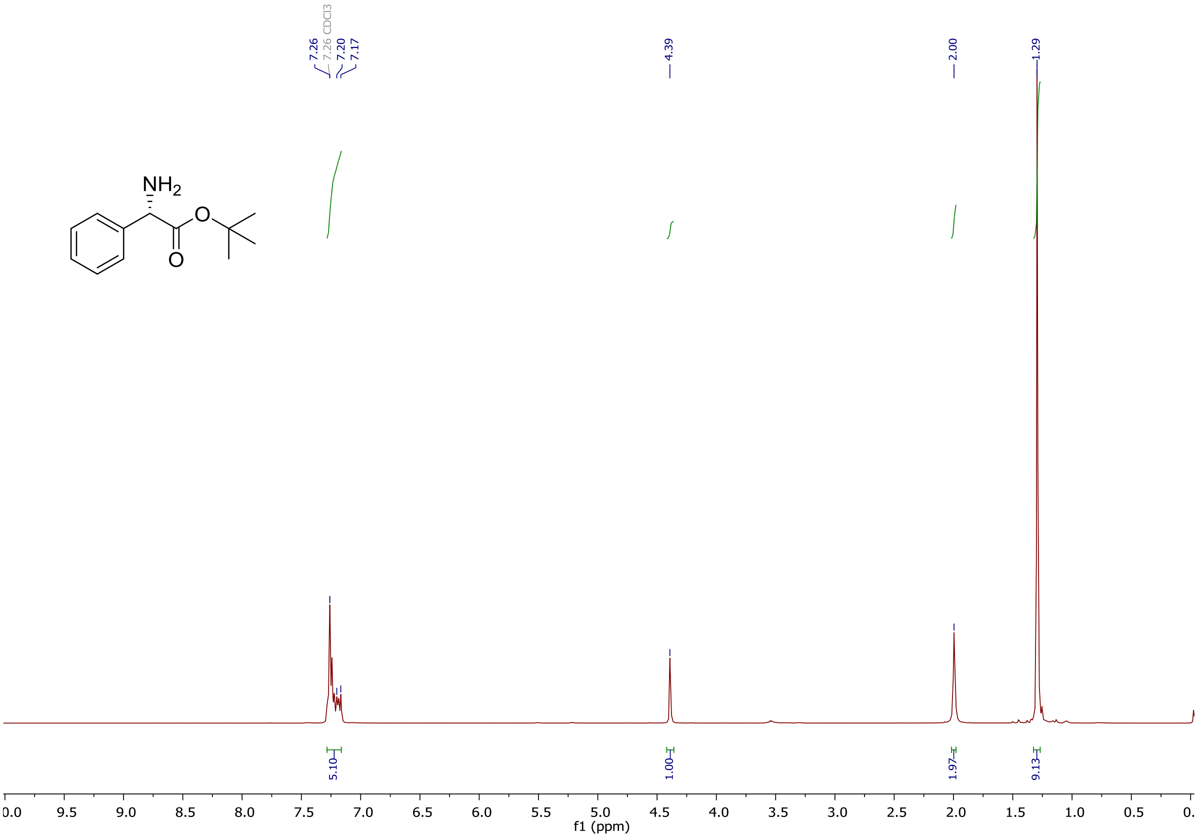
7.26
7.26 CDCl3
7.20
7.17
4.39
2.00
1.29
NH2
O
O
5.10
1.00
1.97
9.13
0.0 9.5 9.0 8.5 8.0 7.5 7.0 6.5 6.0 5.5 5.0 4.5 4.0 3.5 3.0 2.5 2.0 1.5 1.0 0.5 0.
f1 (ppm)

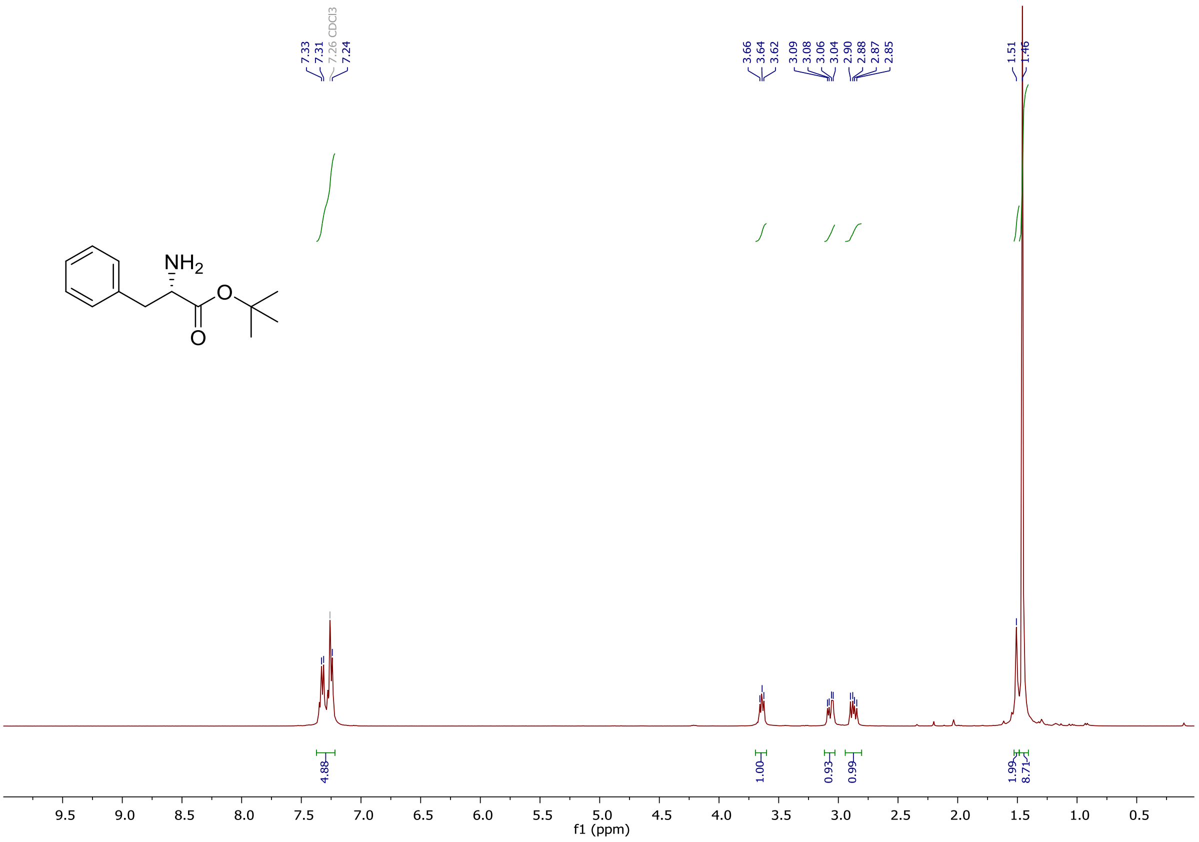

7.33
7.31
7.26 CDCl3
7.24
3.66
3.64
3.62
3.09
3.08
3.06
3.04
2.90
2.88
2.87
2.85
1.51
1.46
NH2
O
O
4.88
1.00
0.93
0.99
1.99
8.71
9.5 9.0 8.5 8.0 7.5 7.0 6.5 6.0 5.5 5.0 4.5 4.0 3.5 3.0 2.5 2.0 1.5 1.0 0.5
f1 (ppm)

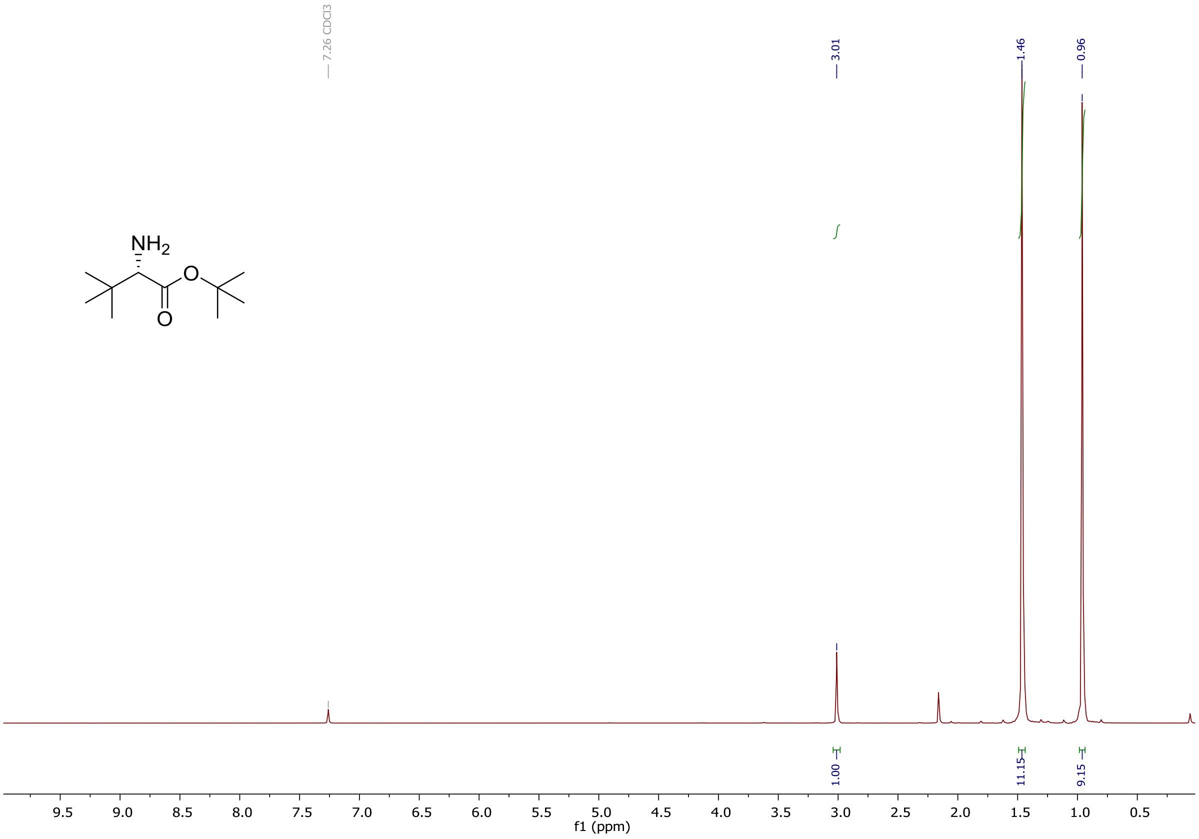

7.26 CDCl3
3.01
1.46
0.96
NH2
O
O
1.00
11.15
9.15
9.5 9.0 8.5 8.0 7.5 7.0 6.5 6.0 5.5 5.0 4.5 4.0 3.5 3.0 2.5 2.0 1.5 1.0 0.5
f1 (ppm)

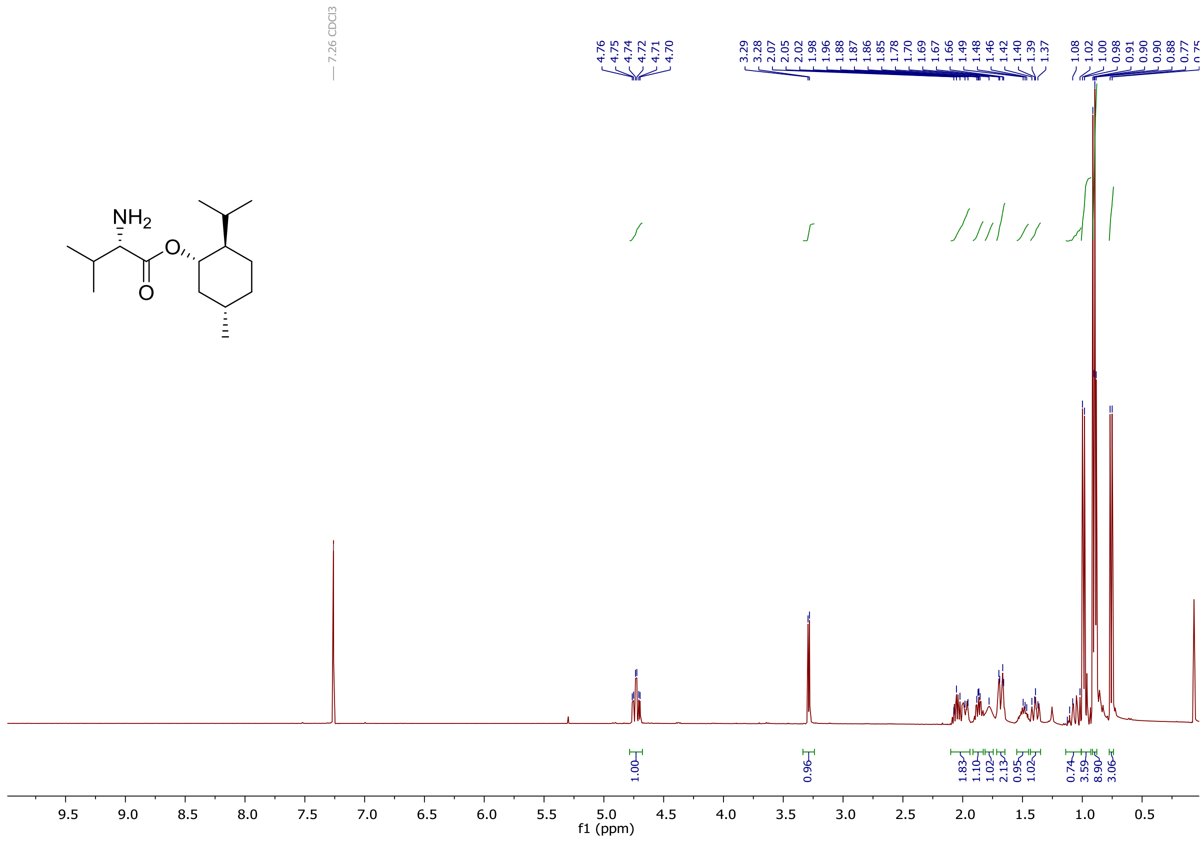
7.26 CDCl3
4.76
4.75
4.74
4.72
4.71
4.70
3.29
3.28
2.07
2.05
2.02
1.98
1.96
1.88
1.87
1.86
1.85
1.78
1.70
1.69
1.67
1.66
1.49
1.48
1.46
1.42
1.40
1.39
1.37
1.08
1.02
1.00
0.98
0.91
0.90
0.90
0.88
0.77
NH2
O
O
1.00
0.96
1.83
1.10
1.02
2.13
0.95
1.02
0.74
3.59
8.90
3.06
9.5
9.0
8.5
8.0
7.5
7.0
6.5
6.0
5.5
5.0
4.5
4.0
3.5
3.0
2.5
2.0
1.5
1.0
0.5
f1 (ppm)

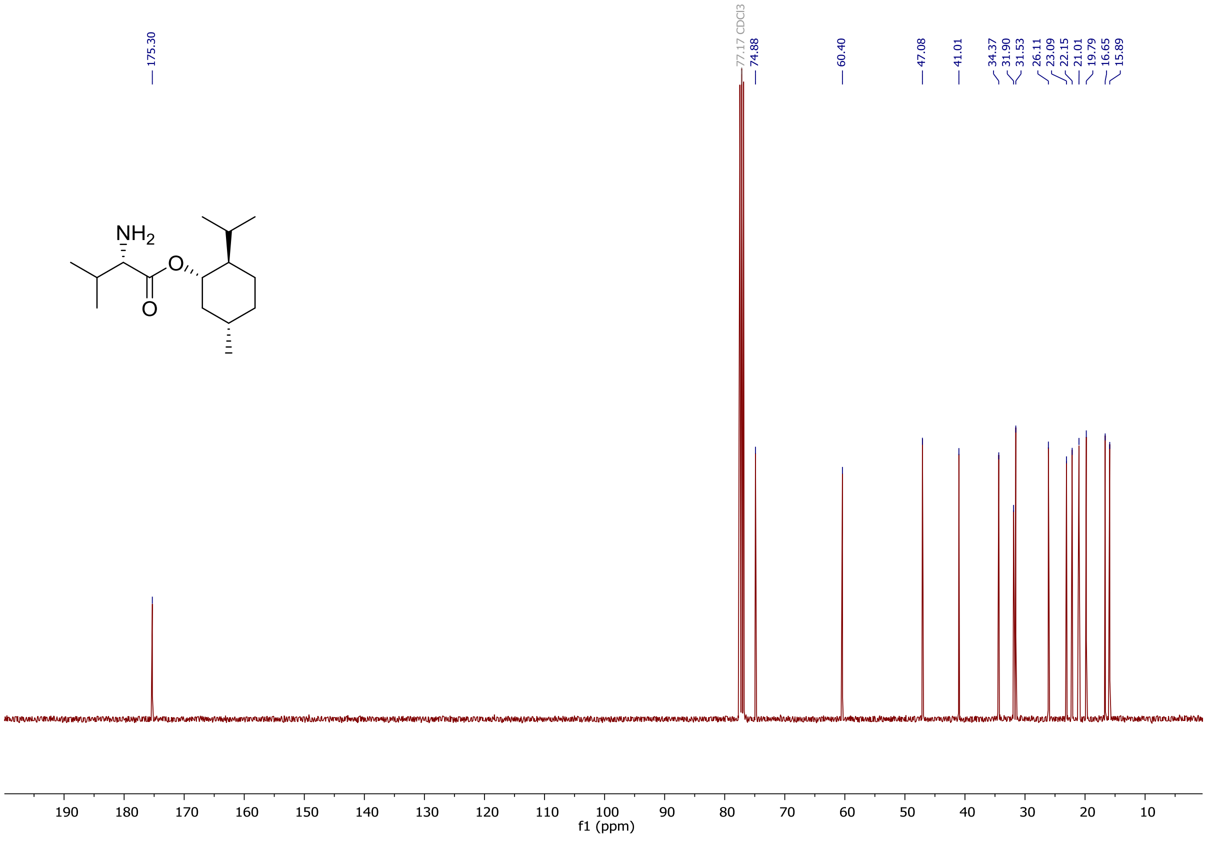
175.30
77.17 CDCl3
74.88
60.40
47.08
41.01
34.37
31.90
31.53
26.11
23.09
22.15
21.01
19.79
16.65
15.89
NH2
O
O
190
180
170
160
150
140
130
120
110
100
90
80
70
60
50
40
30
20
10
f1 (ppm)

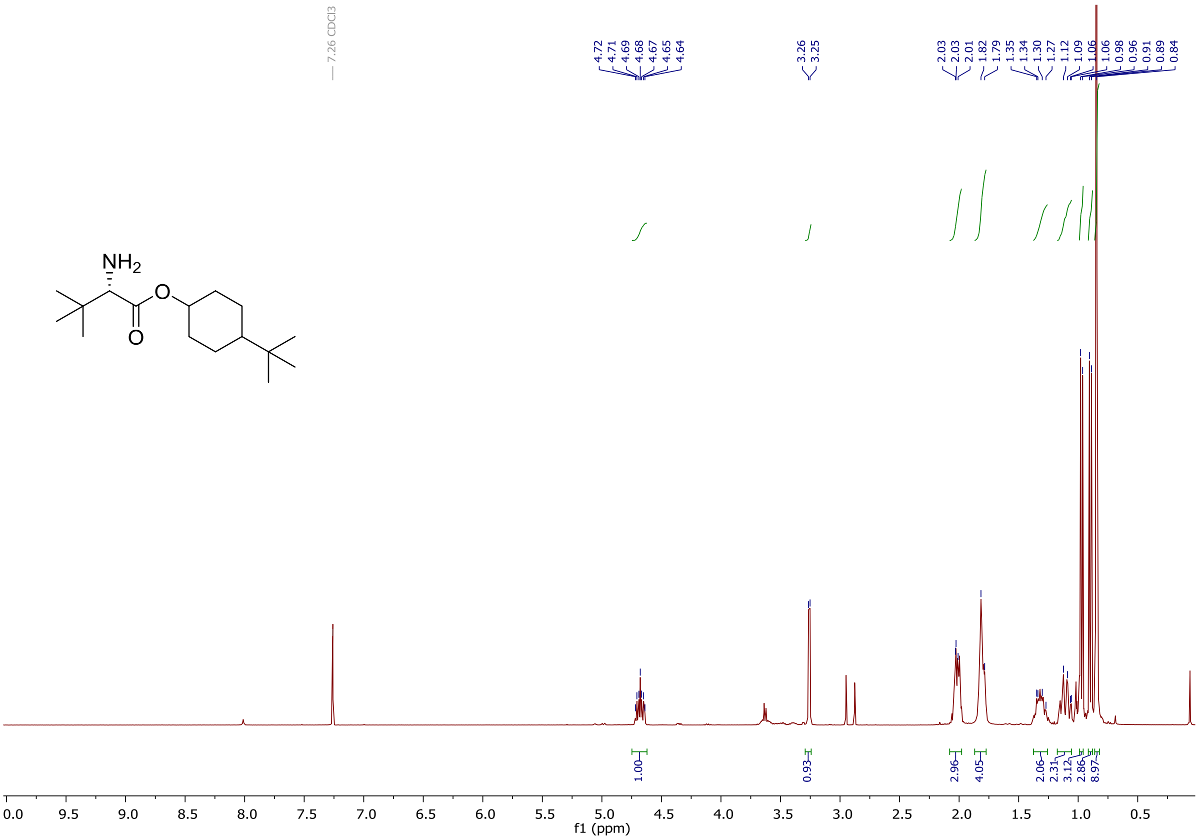
7.26 CDCl3
4.72 4.71 4.69 4.68 4.67 4.65 4.64
3.26 3.25
2.03 2.03 2.01 1.82 1.79 1.35 1.34 1.30 1.27 1.12 1.09 1.06 1.06 0.98 0.96 0.91 0.89 0.84
NH2
O
O
1.00
0.93
2.96
4.05
2.06 2.31 3.12 2.86 8.97
0.0 9.5 9.0 8.5 8.0 7.5 7.0 6.5 6.0 5.5 5.0 4.5 4.0 3.5 3.0 2.5 2.0 1.5 1.0 0.5
f1 (ppm)

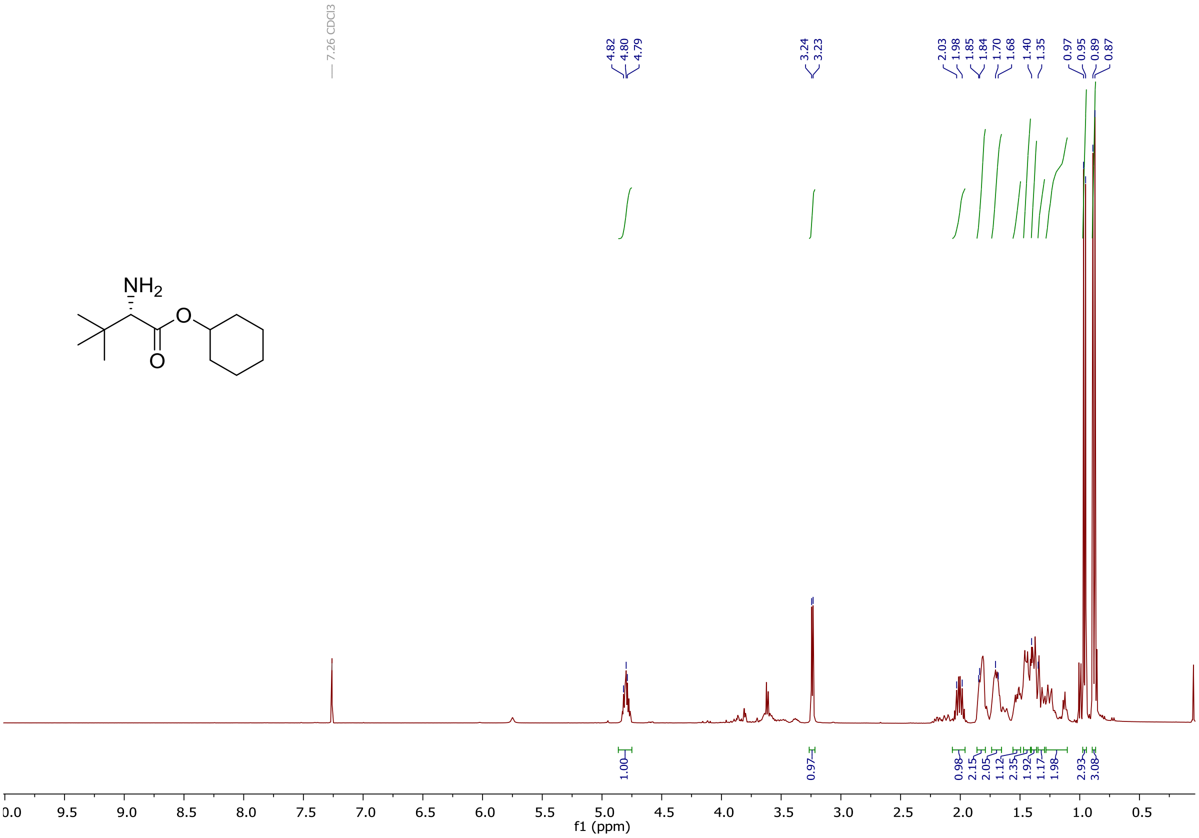
7.26 CDCl3
4.82 4.80 4.79
3.24 3.23
2.03 1.98 1.85 1.84 1.70 1.68 1.40 1.35 0.97 0.95 0.89 0.87
NH2
O
O
1.00
0.97
0.98 2.15 2.05 1.12 2.35 1.92 1.17 1.98 2.93 3.08
0.0 9.5 9.0 8.5 8.0 7.5 7.0 6.5 6.0 5.5 5.0 4.5 4.0 3.5 3.0 2.5 2.0 1.5 1.0 0.5
f1 (ppm)

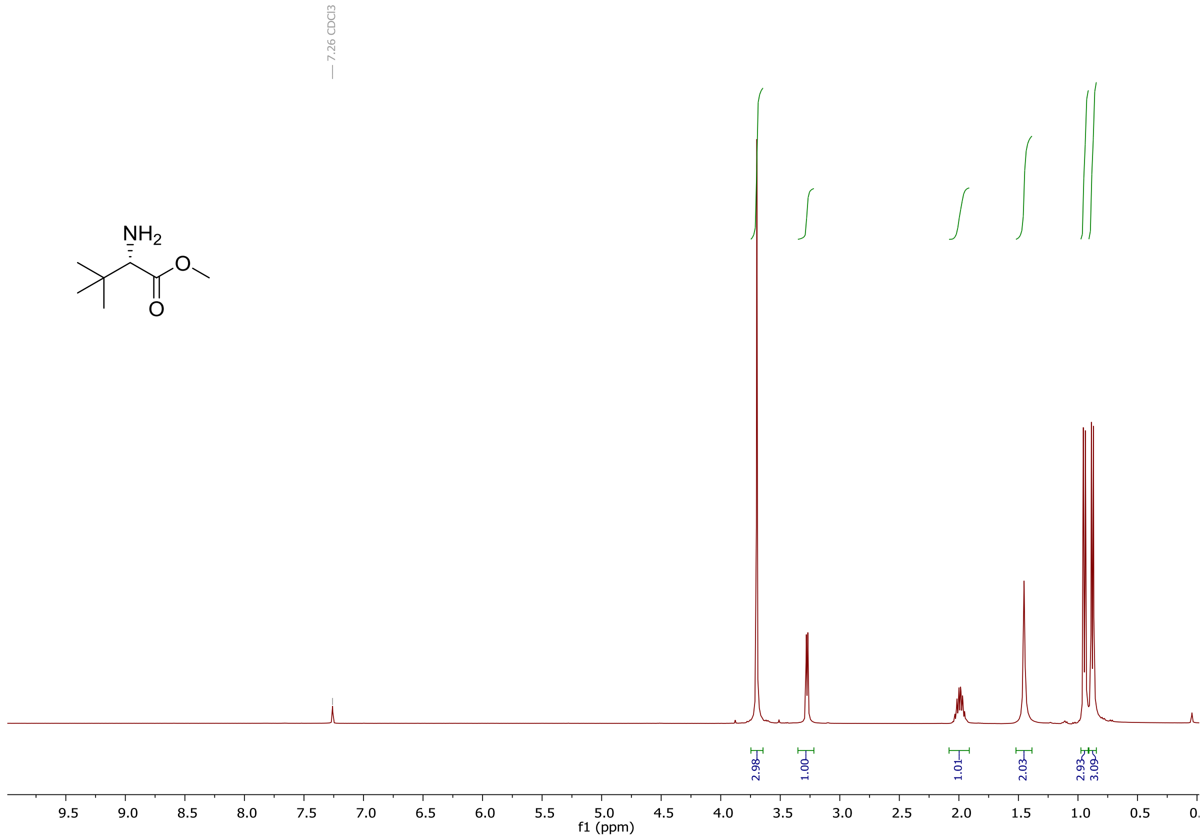
7.26 CDCl3
NH2
O
O
2.98
1.00
1.01
2.03
2.93
3.09
f1 (ppm)

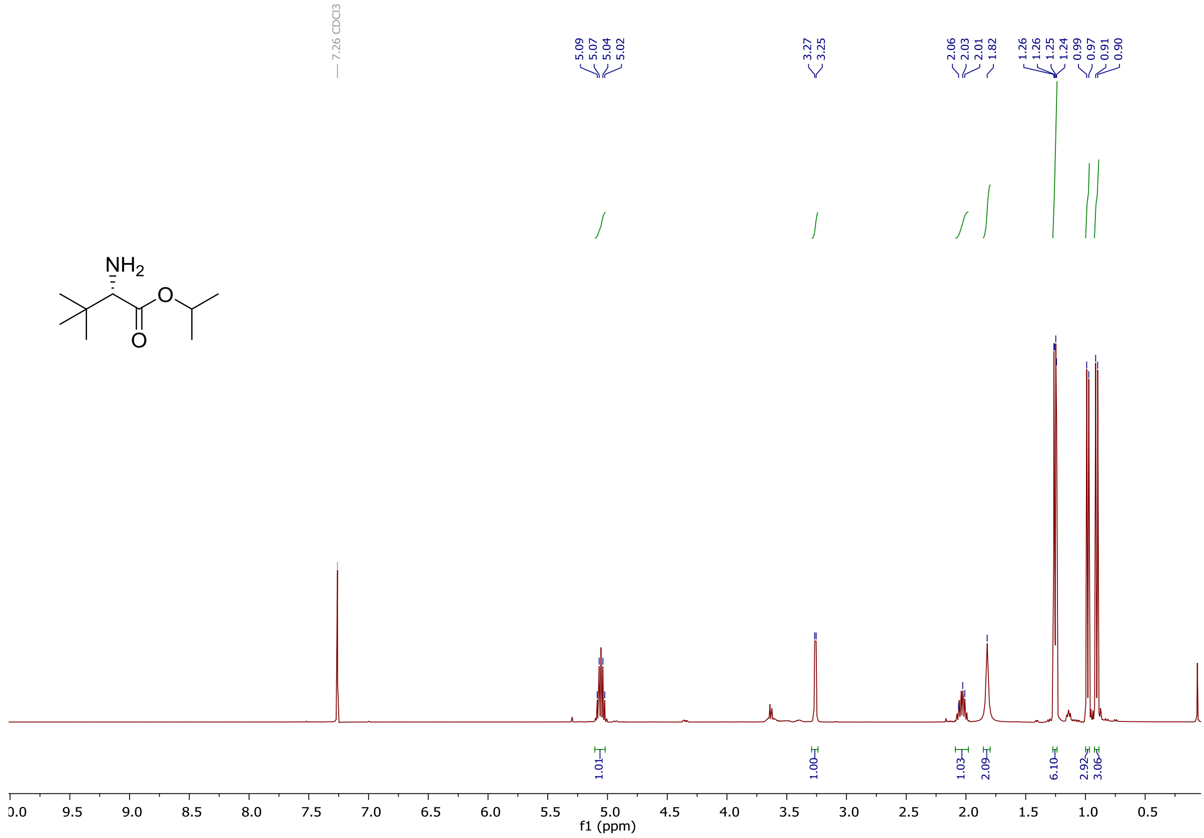
7.26 CDCl3
5.09
5.07
5.04
5.02
3.27
3.25
2.06
2.03
2.01
1.82
1.26
1.26
1.25
1.24
0.99
0.97
0.91
0.90
NH2
O
O
1.01
1.00
1.03
2.09
6.10
2.92
3.06
f1 (ppm)

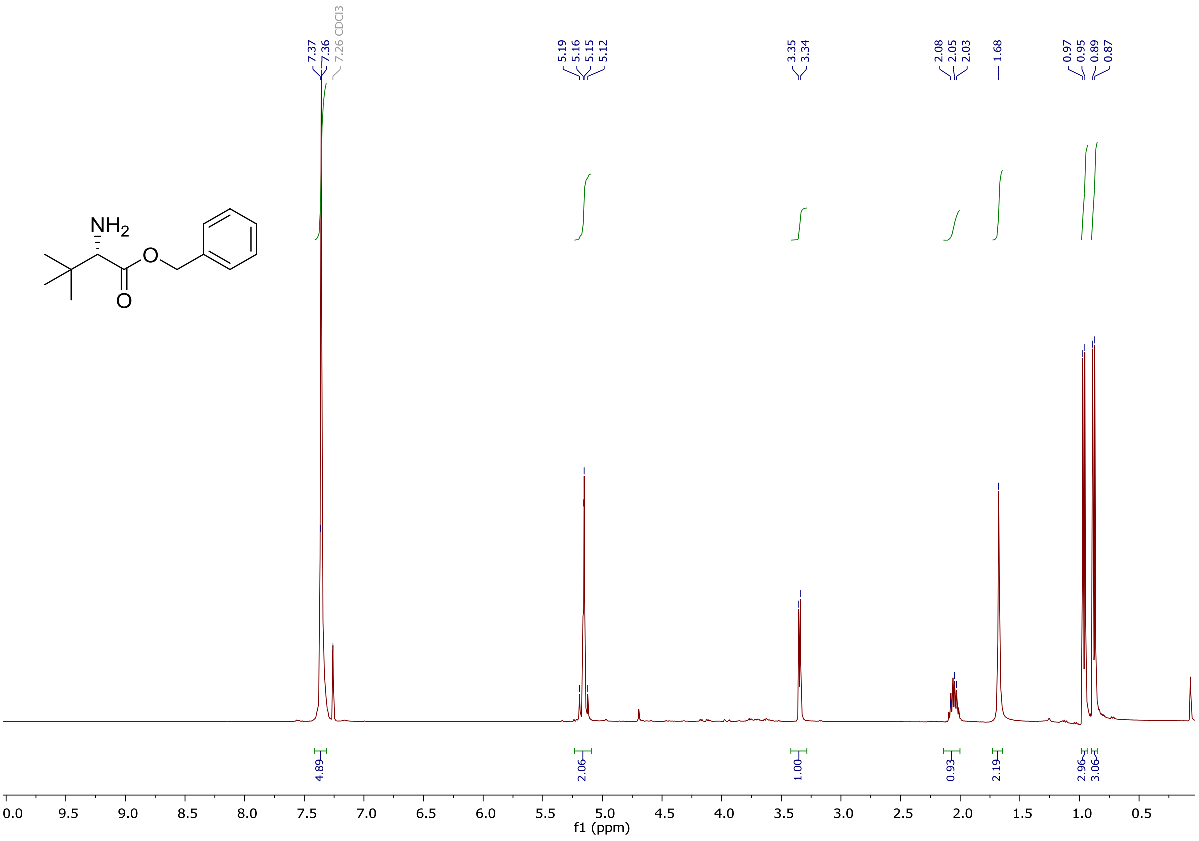
NH2
O
O
7.37
7.36
7.26 CDCl3
5.19
5.16
5.15
5.12
3.35
3.34
2.08
2.05
2.03
1.68
0.97
0.95
0.89
0.87
4.89
2.06
1.00
0.93
2.19
2.96
3.06
0.0
9.5
9.0
8.5
8.0
7.5
7.0
6.5
6.0
5.5
5.0
4.5
4.0
3.5
3.0
2.5
2.0
1.5
1.0
0.5
f1 (ppm)

9. NMR specta of phosphoric acids and its intermediates

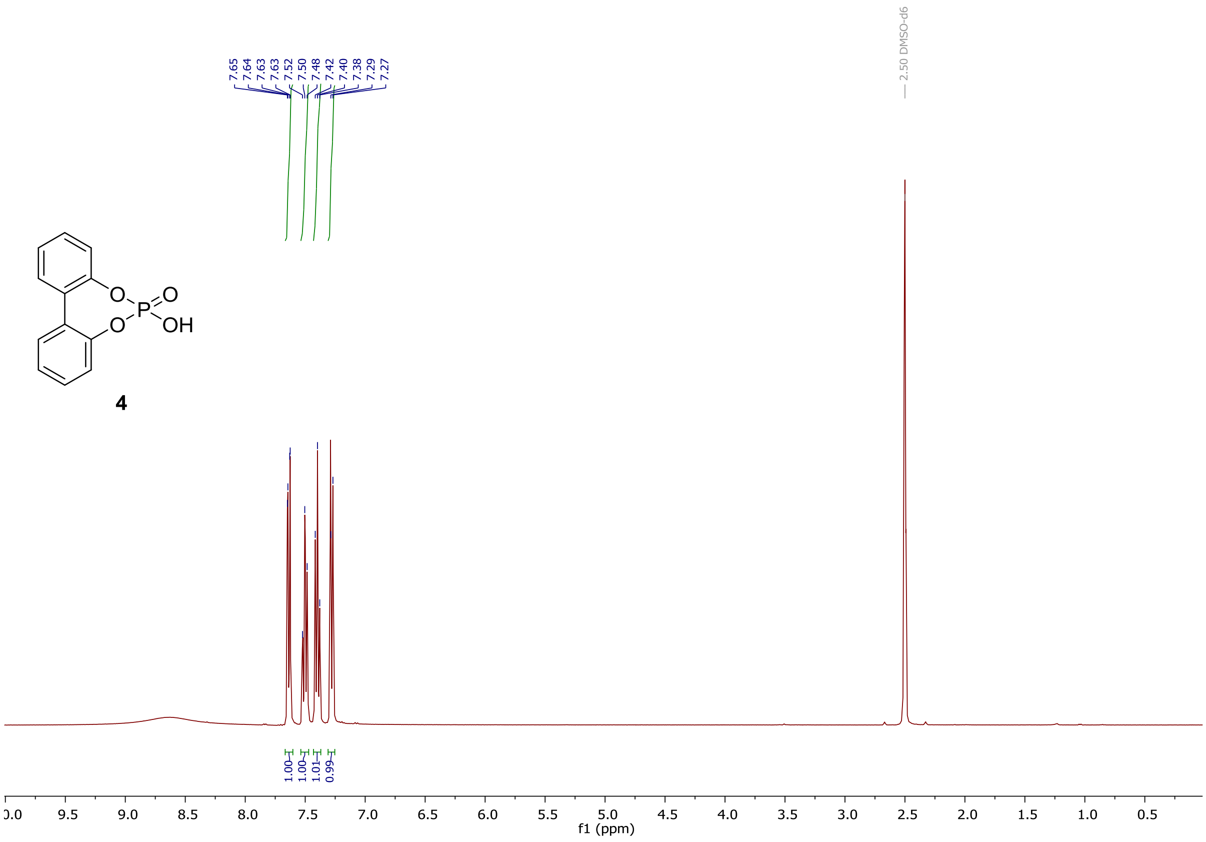

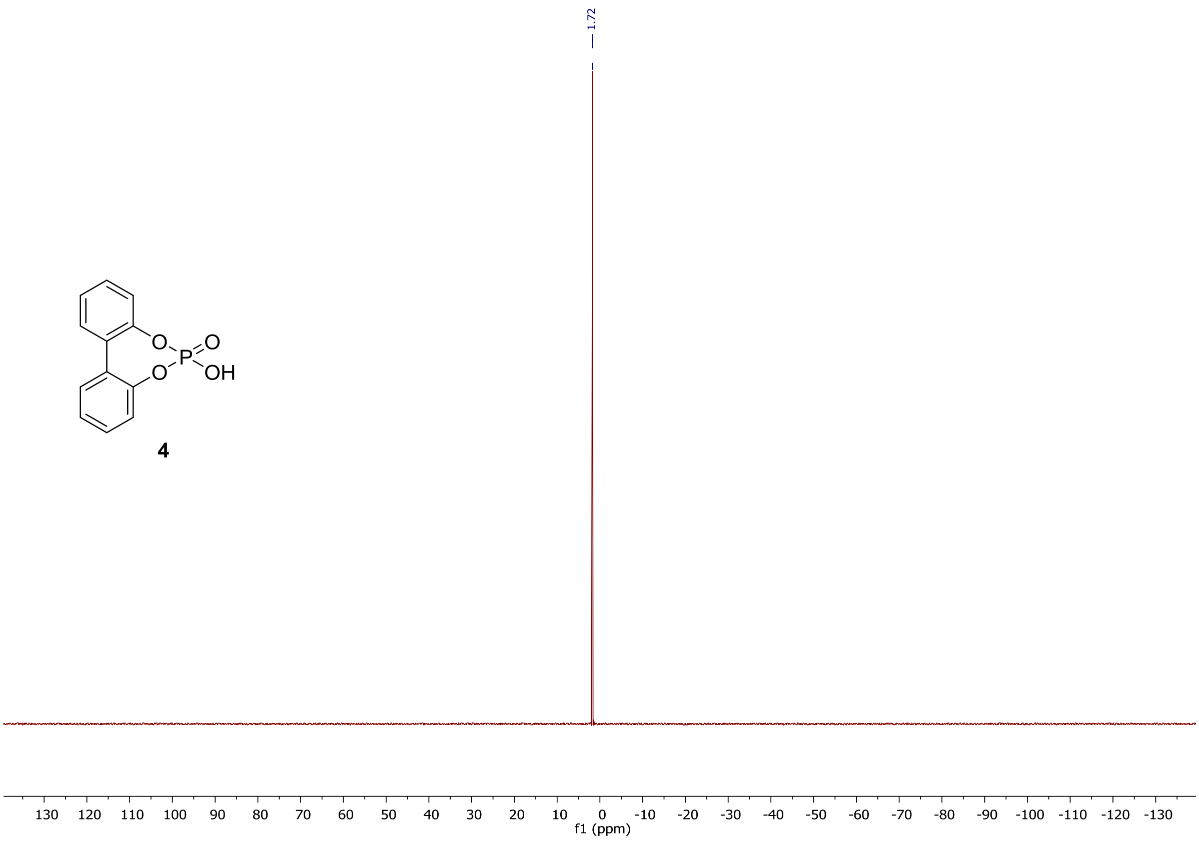

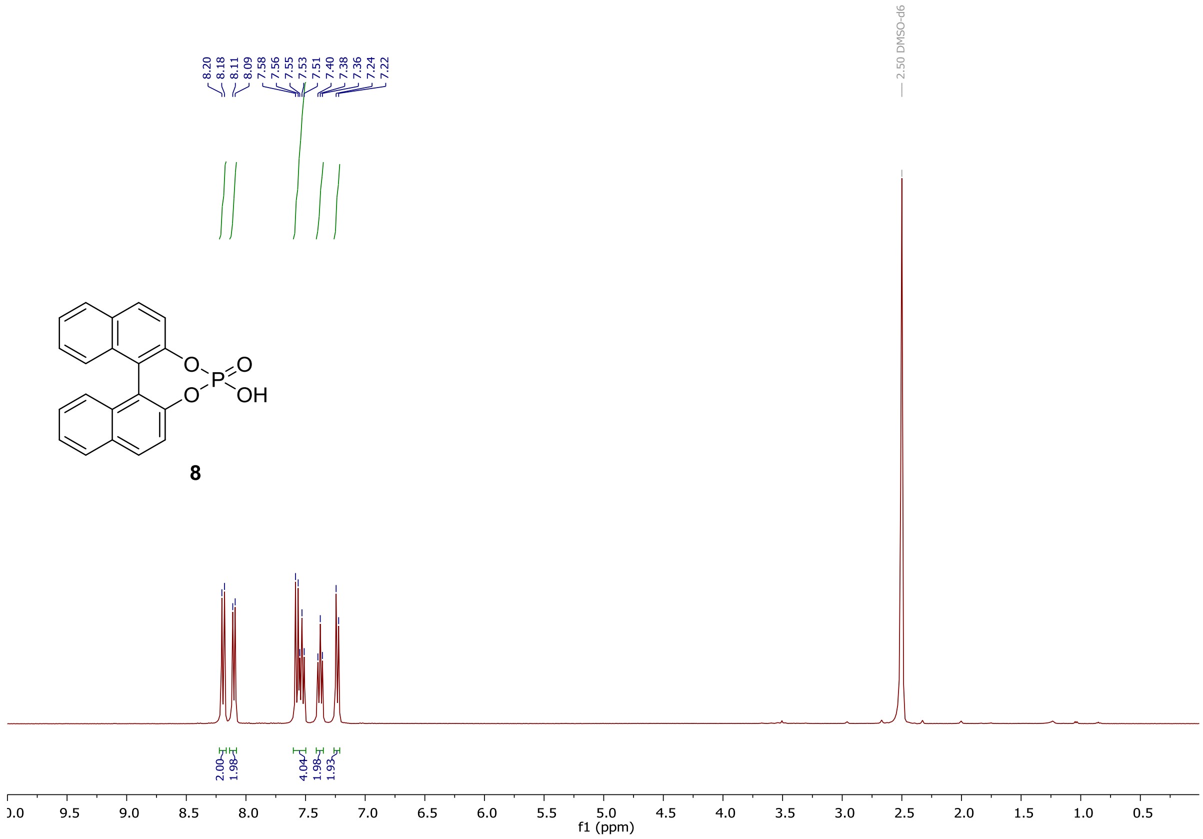
8.20
8.18
8.11
8.09
7.58
7.56
7.55
7.53
7.51
7.40
7.38
7.36
7.24
7.22
2.50 DMSO-d6
O
P
O
O
OH
8
2.00
1.98
4.04
1.98
1.93
f1 (ppm)

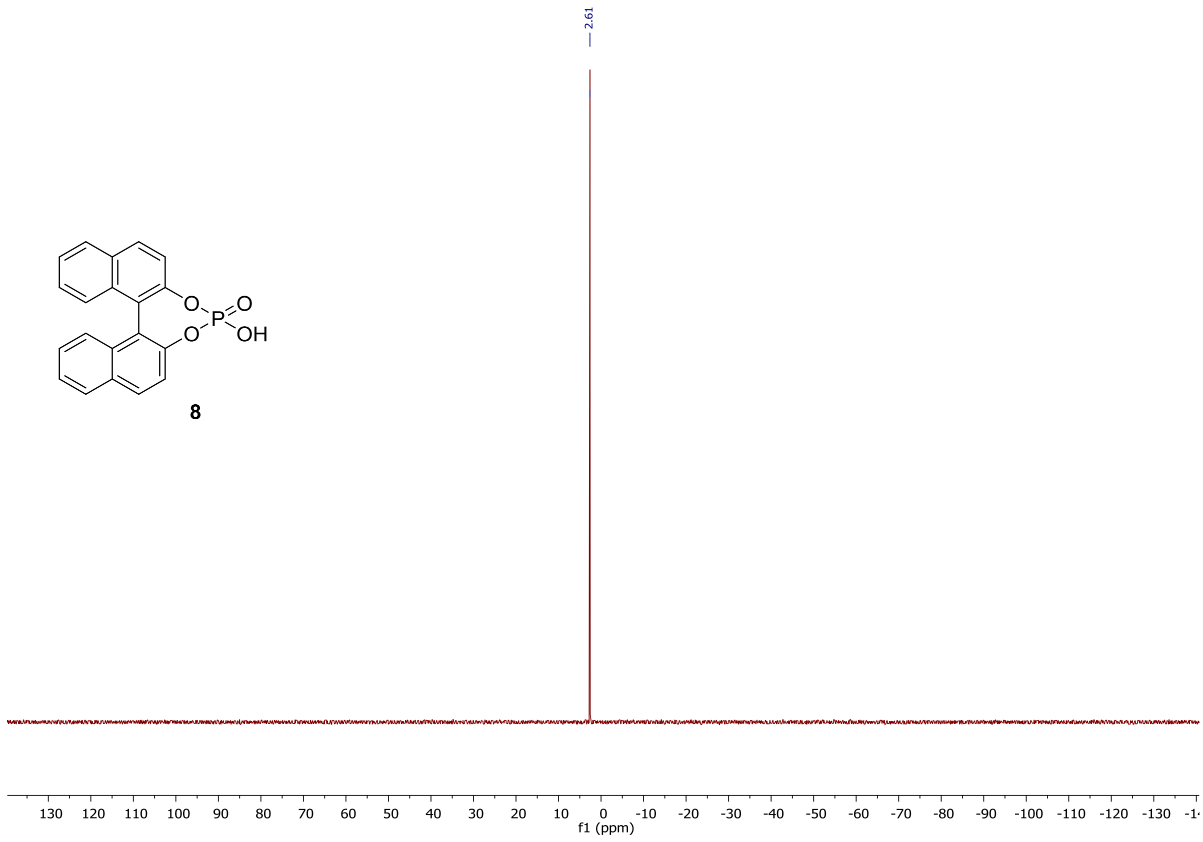
2.61
O
P
O
O
OH
8
f1 (ppm)

mentyhl ester II

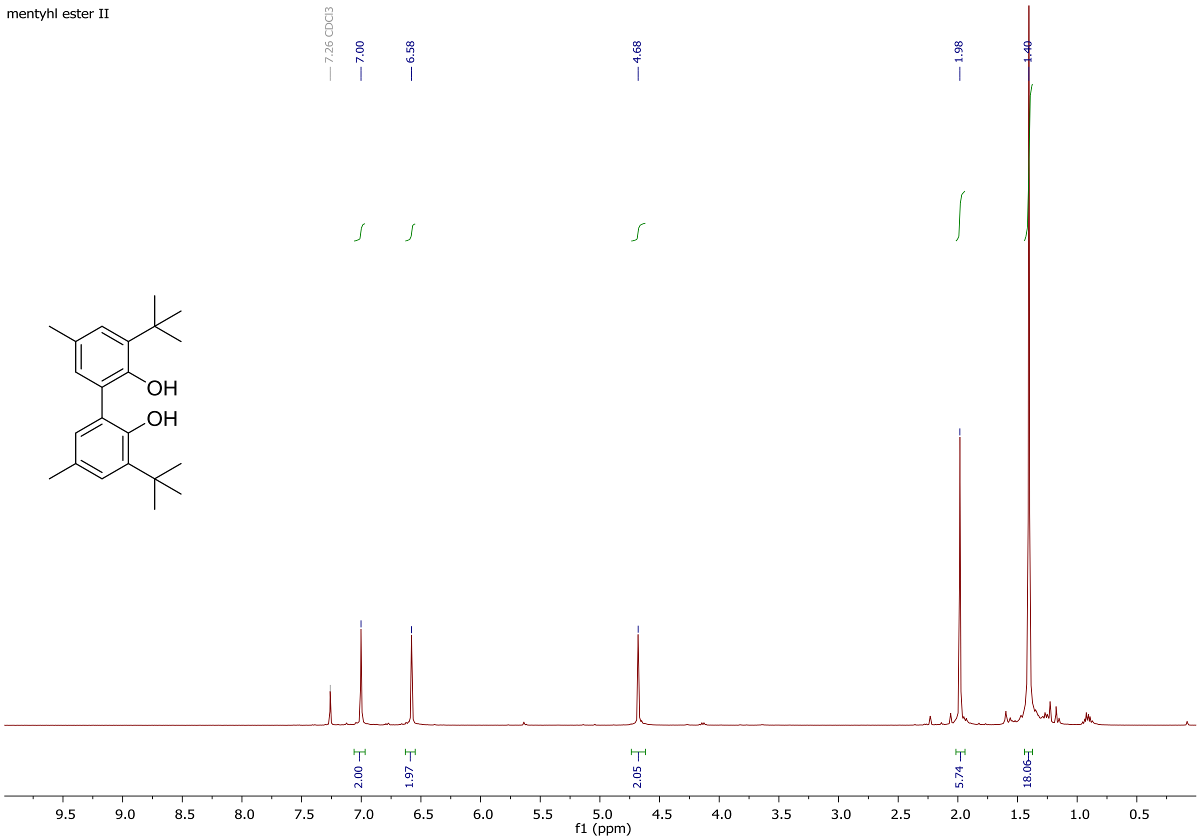

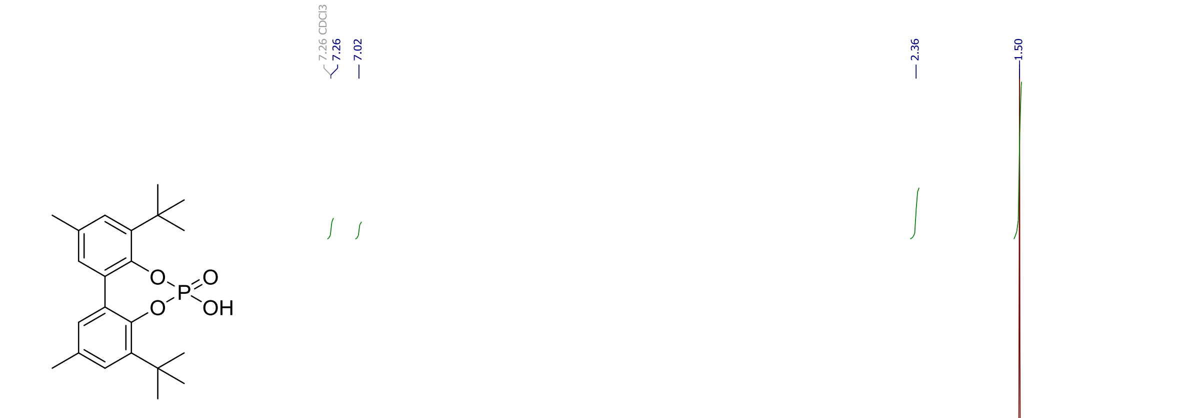

5

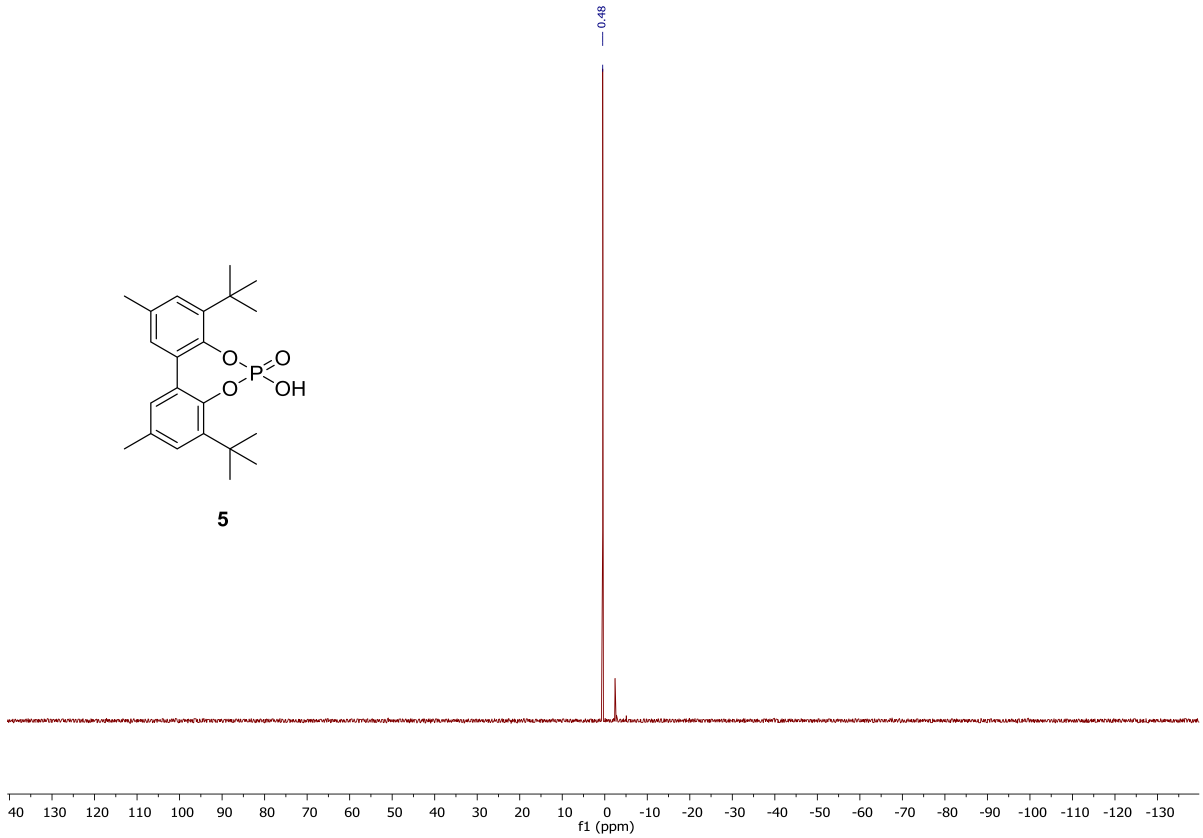
O
O
P
O
OH
5
0.48
f1 (ppm)

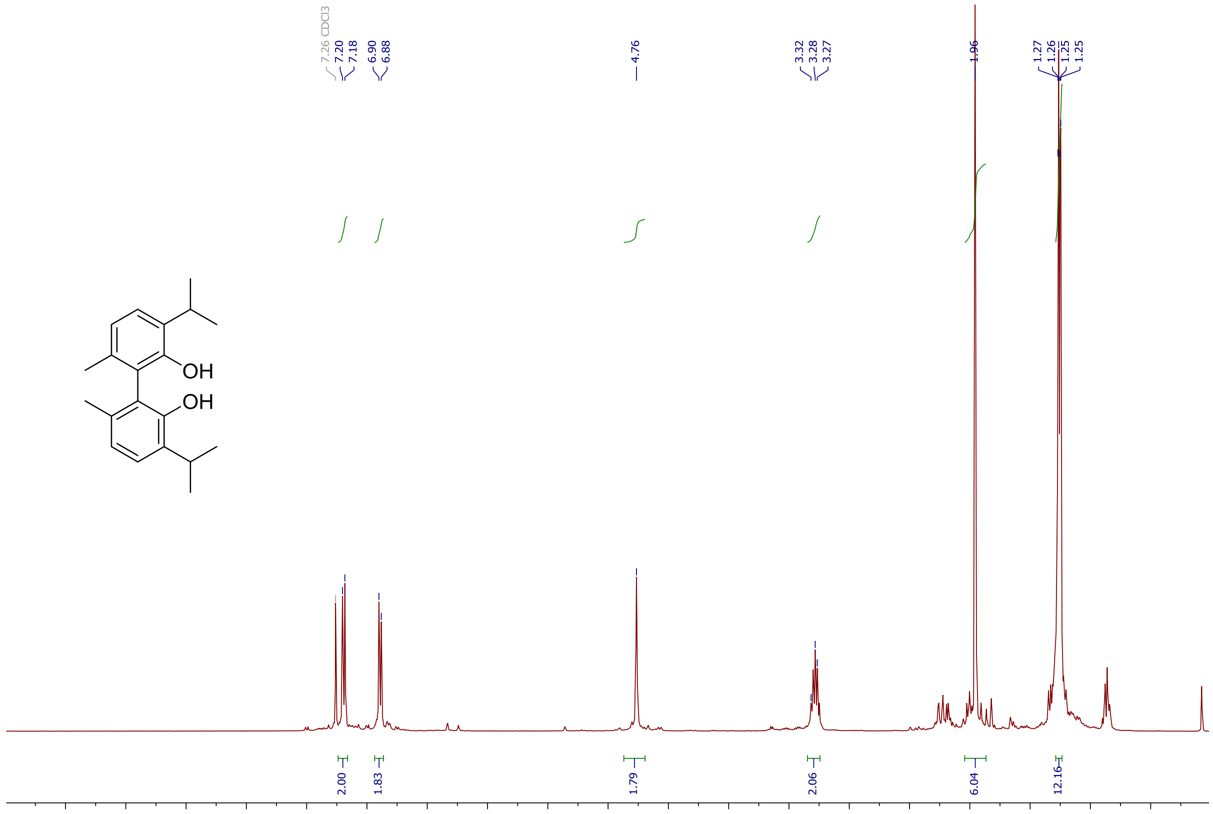
OH
OH
7.26 CDCl3
7.20
7.18
6.90
6.88
4.76
3.32
3.28
3.27
1.96
1.27
1.26
1.25
1.25
2.00
1.83
1.79
2.06
6.04
12.16
f1 (ppm)

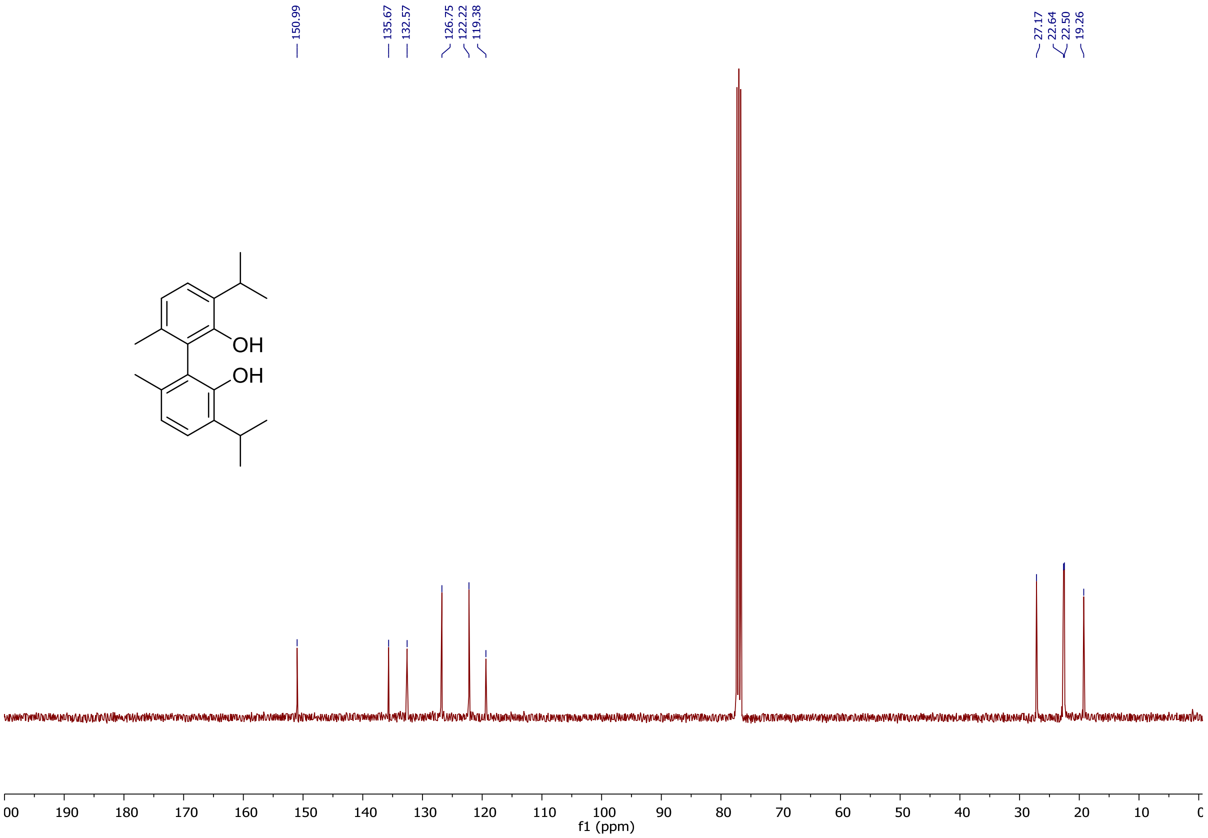
150.99
135.67
132.57
126.75
122.22
119.38
27.17
22.64
22.50
19.26
OH
OH
f1 (ppm)

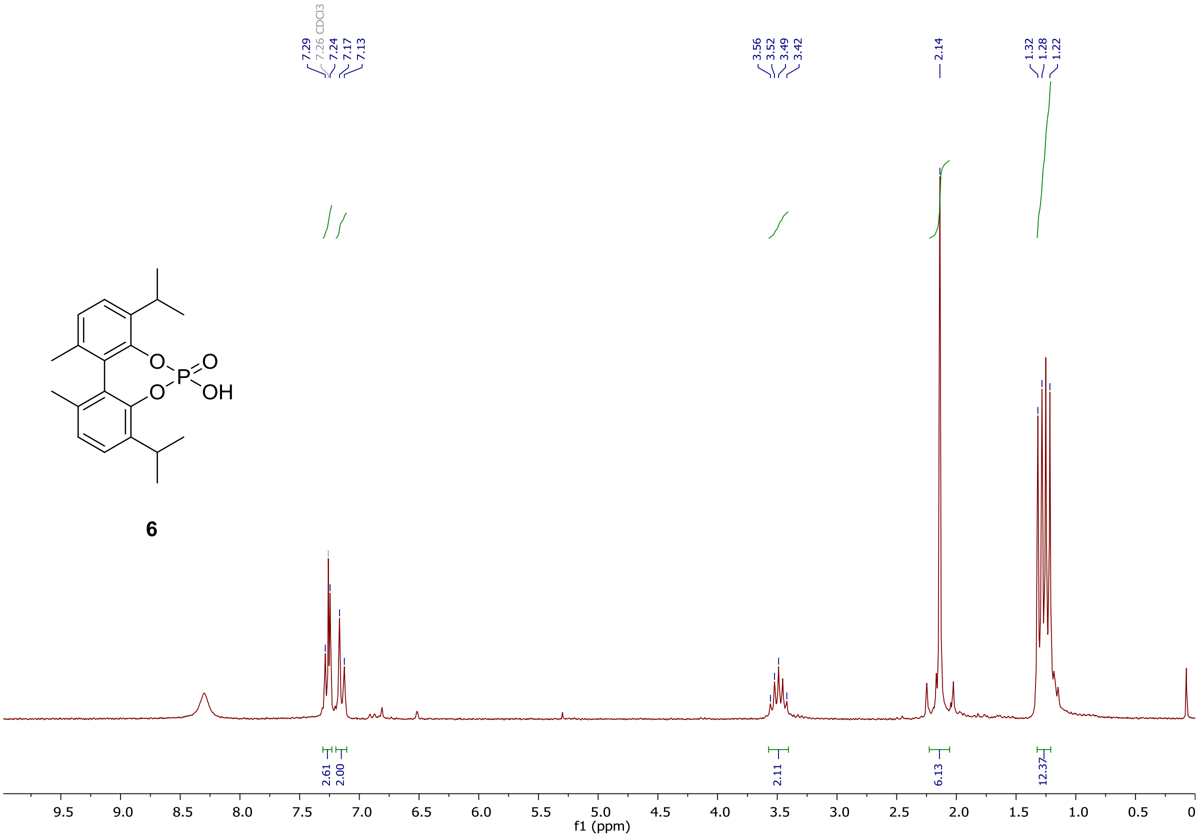
O
P
O
OH
O
7.29
7.26 CDCl3
7.24
7.17
7.13
3.56
3.52
3.49
3.42
2.14
1.32
1.28
1.22
2.61
2.00
2.11
6.13
12.37
f1 (ppm)

6

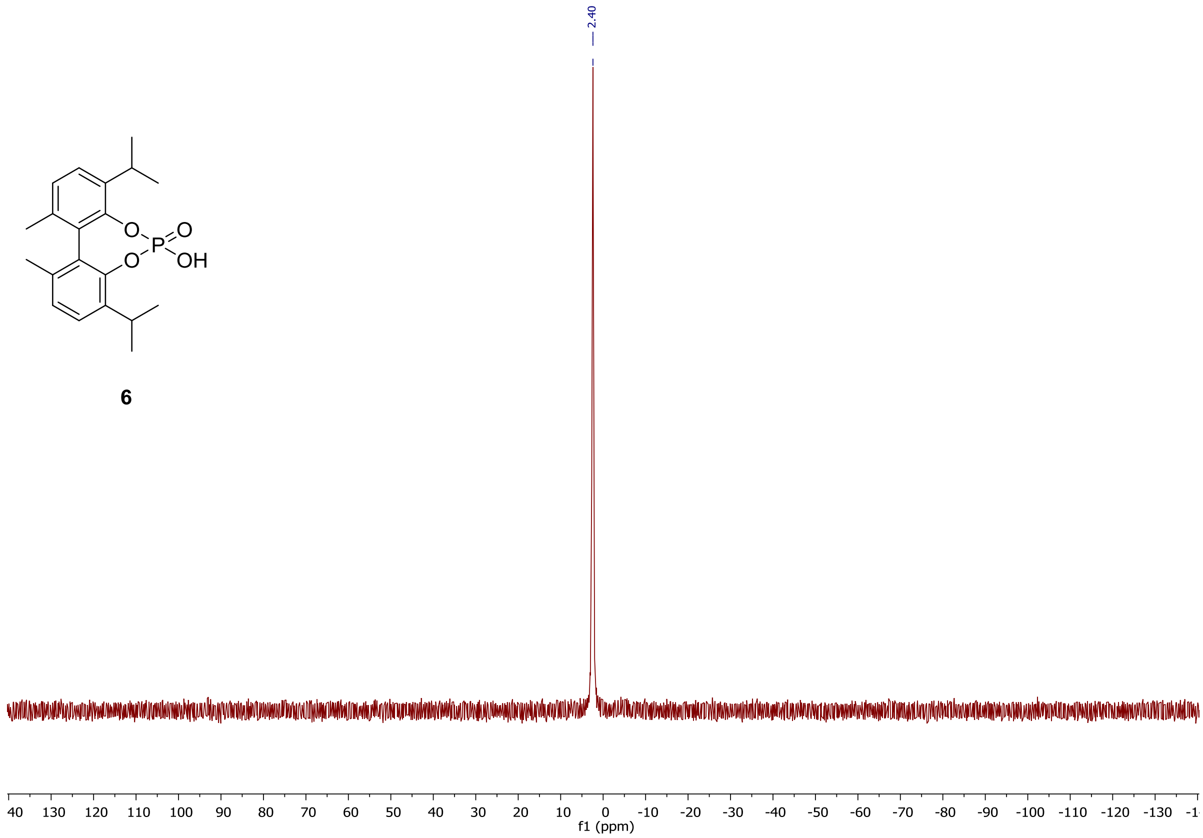
2.40
O
P
O
OH
O
6
f1 (ppm)

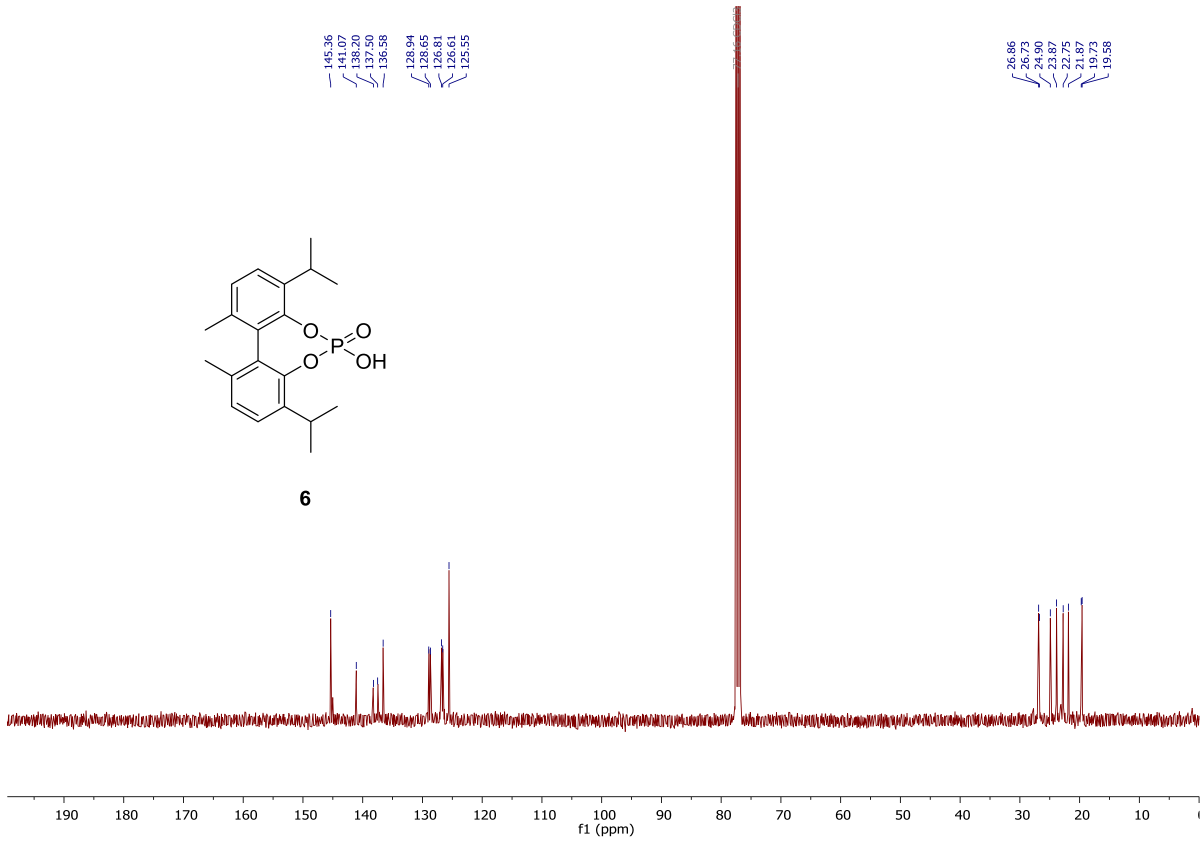
145.36
141.07
138.20
137.50
136.58
128.94
128.65
126.81
126.61
125.55
26.86
26.73
24.90
23.87
22.75
21.87
19.73
19.58
O
P
O
OH
O
6
f1 (ppm)

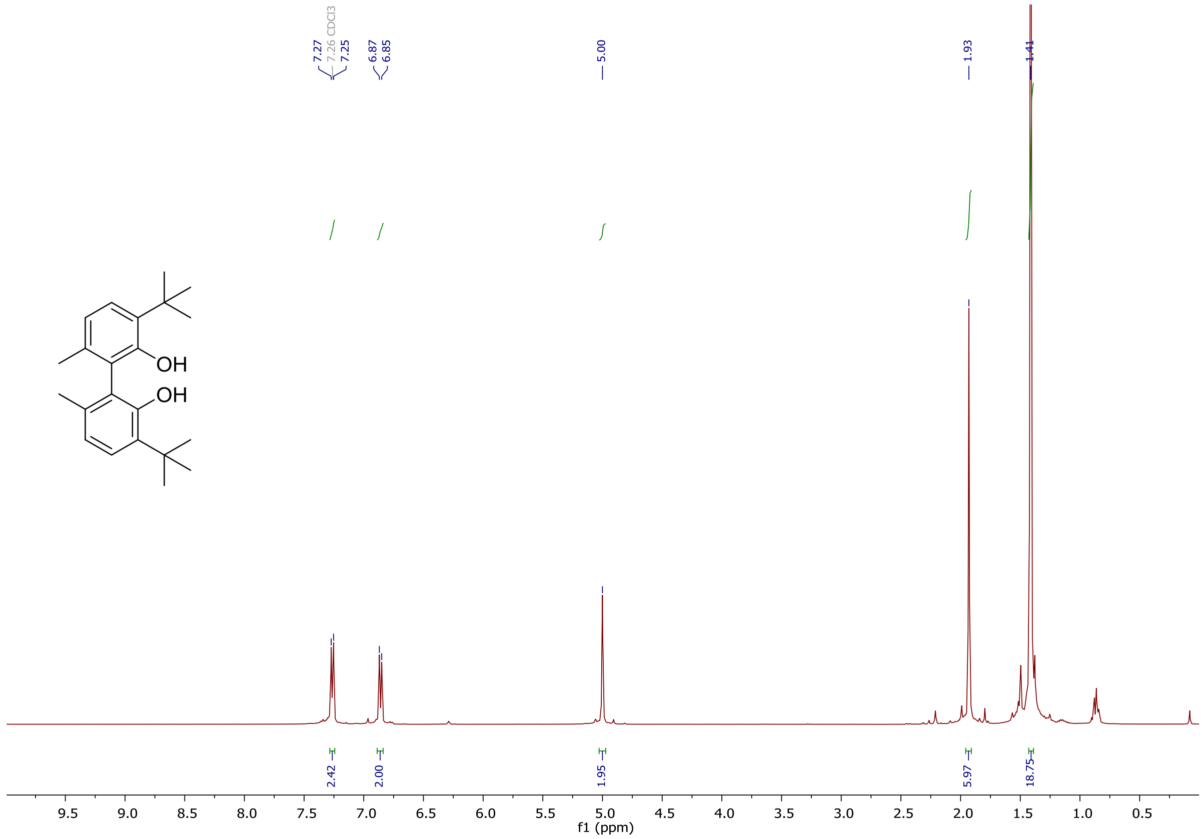
7.27
7.26 CDCl3
7.25
6.87
6.85
5.00
1.93
1.41
OH
OH
2.42
2.00
1.95
5.97
18.75
f1 (ppm)

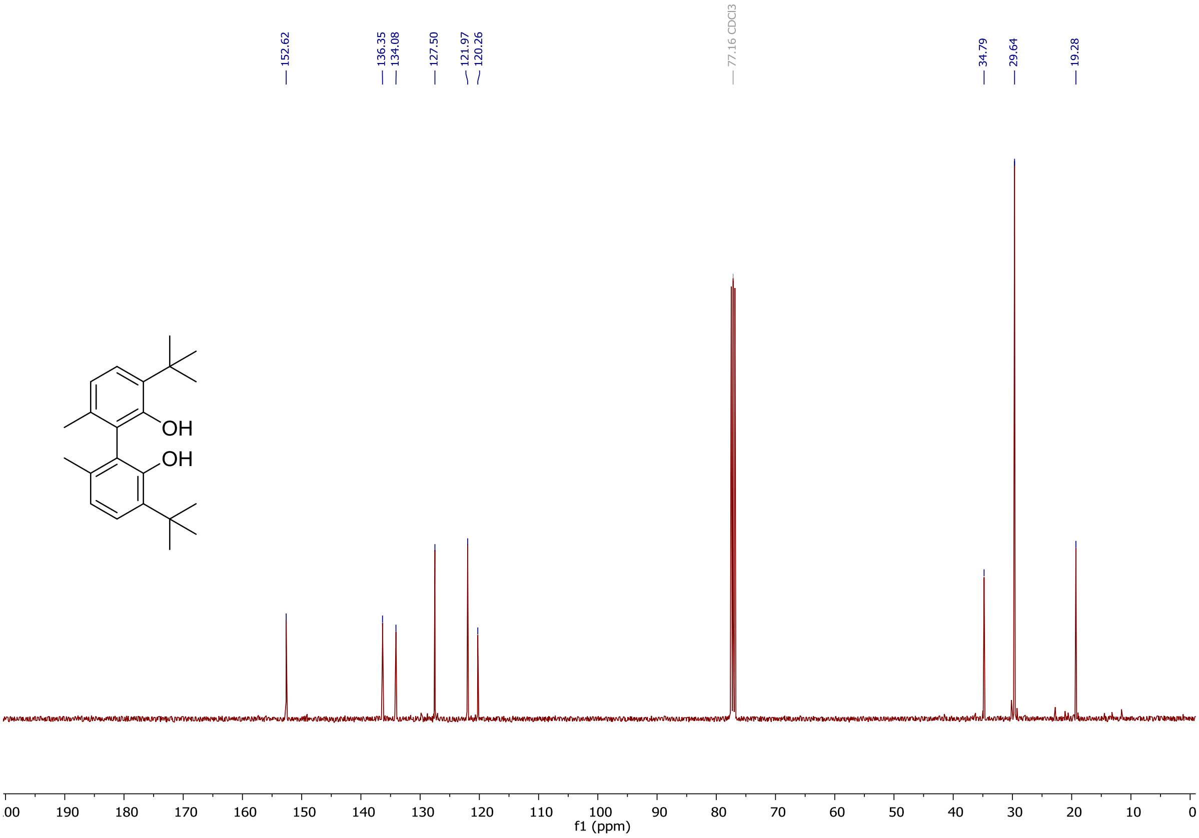
152.62
136.35
134.08
127.50
121.97
120.26
77.16 CDCl3
34.79
29.64
19.28
OH
OH
f1 (ppm)

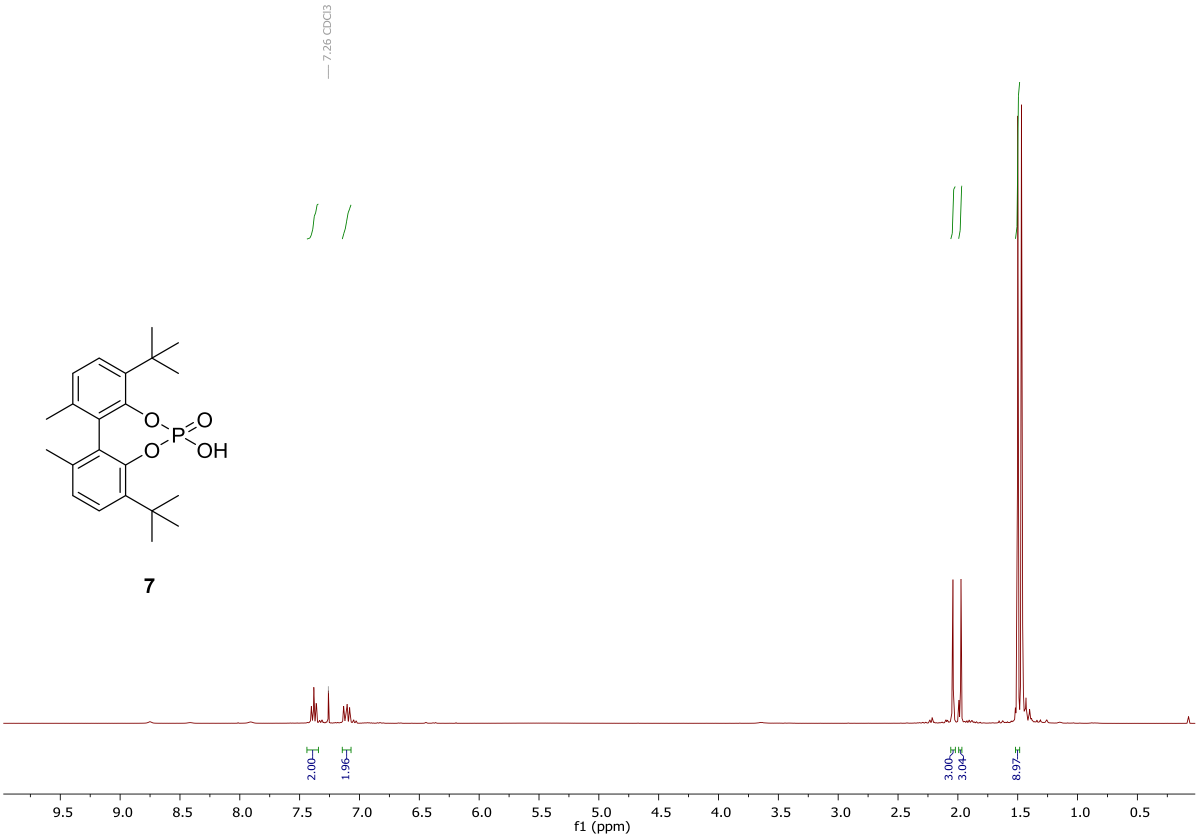

7.26 CDCl3
O
P
O
O
OH
7
2.00
1.96
3.00
3.04
8.97
9.5 9.0 8.5 8.0 7.5 7.0 6.5 6.0 5.5 5.0 4.5 4.0 3.5 3.0 2.5 2.0 1.5 1.0 0.5
f1 (ppm)

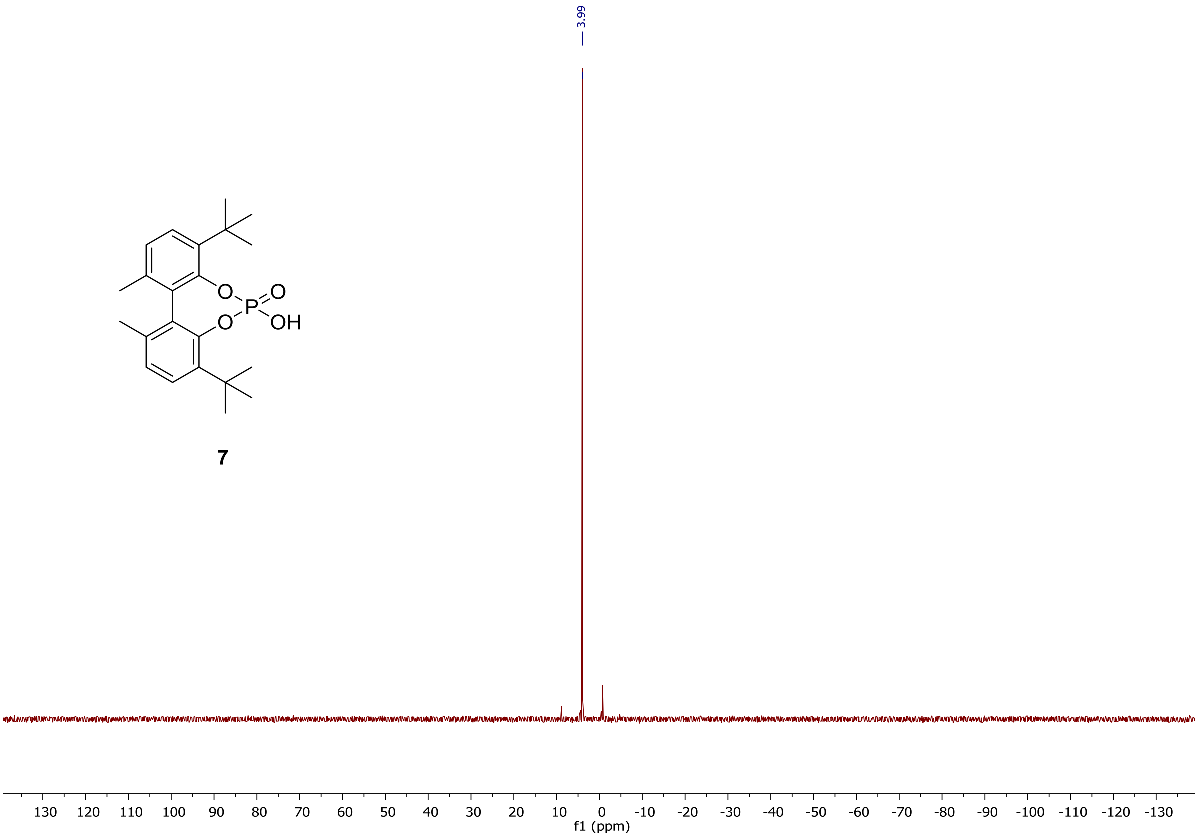

3.99
O
P
O
O
OH
7
130 120 110 100 90 80 70 60 50 40 30 20 10 0 -10 -20 -30 -40 -50 -60 -70 -80 -90 -100 -110 -120 -130
f1 (ppm)

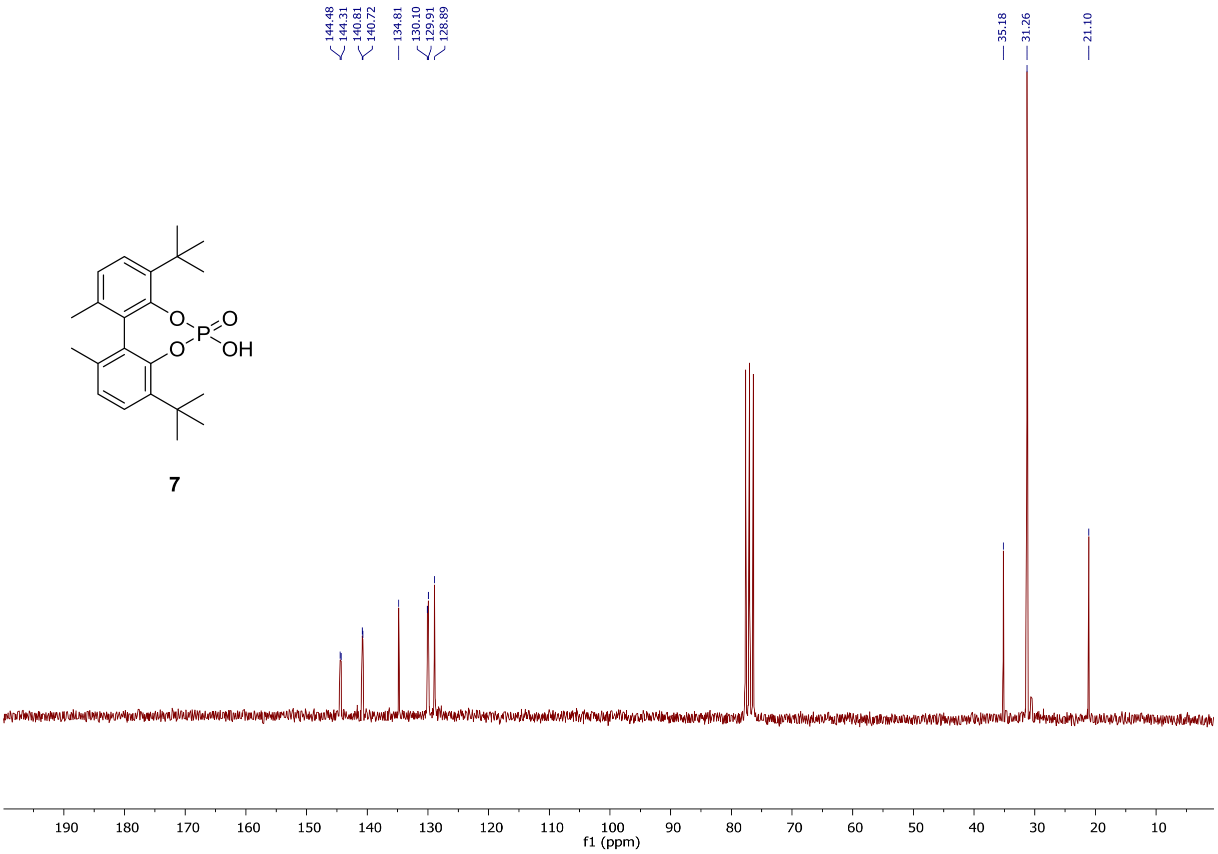
O
P
O
O
OH
7
144.48
144.31
140.81
140.72
134.81
130.10
129.91
128.89
35.18
31.26
21.10
190 180 170 160 150 140 130 120 110 100 90 80 70 60 50 40 30 20 10
f1 (ppm)

10. NMR specta of ATH substrates

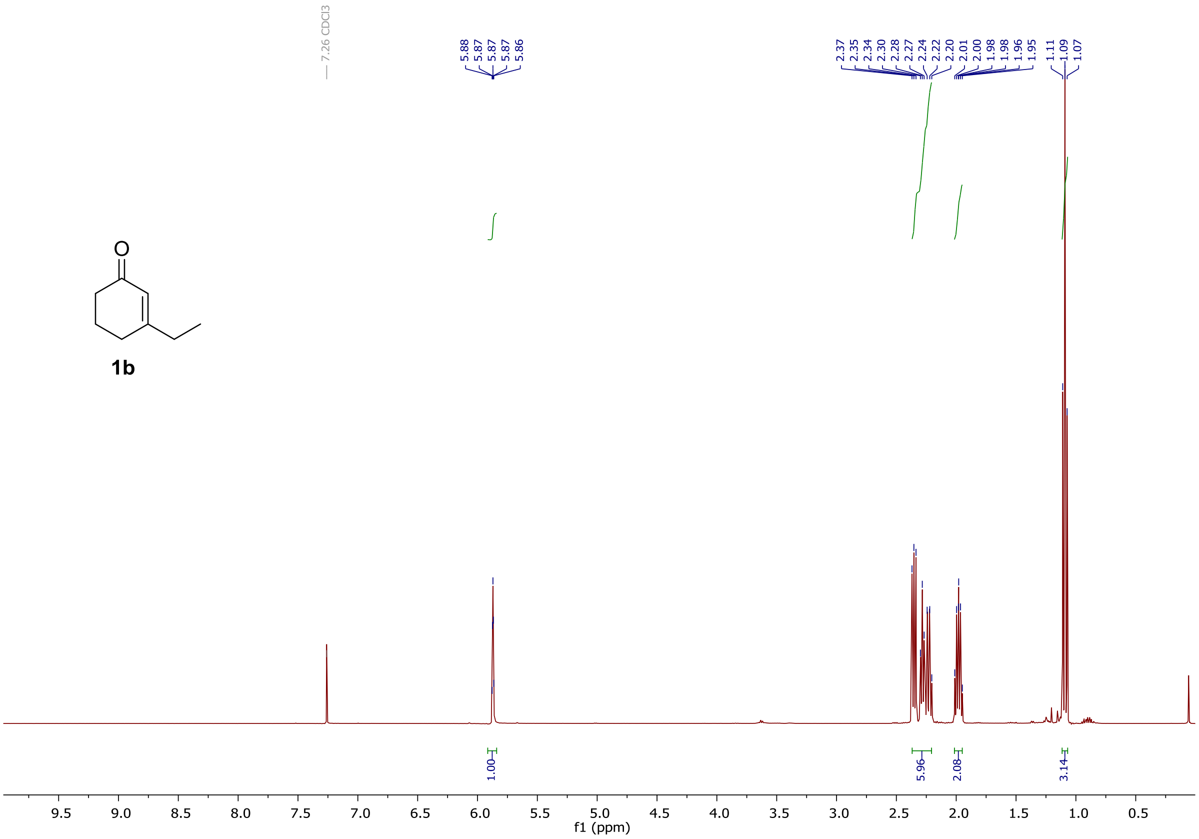

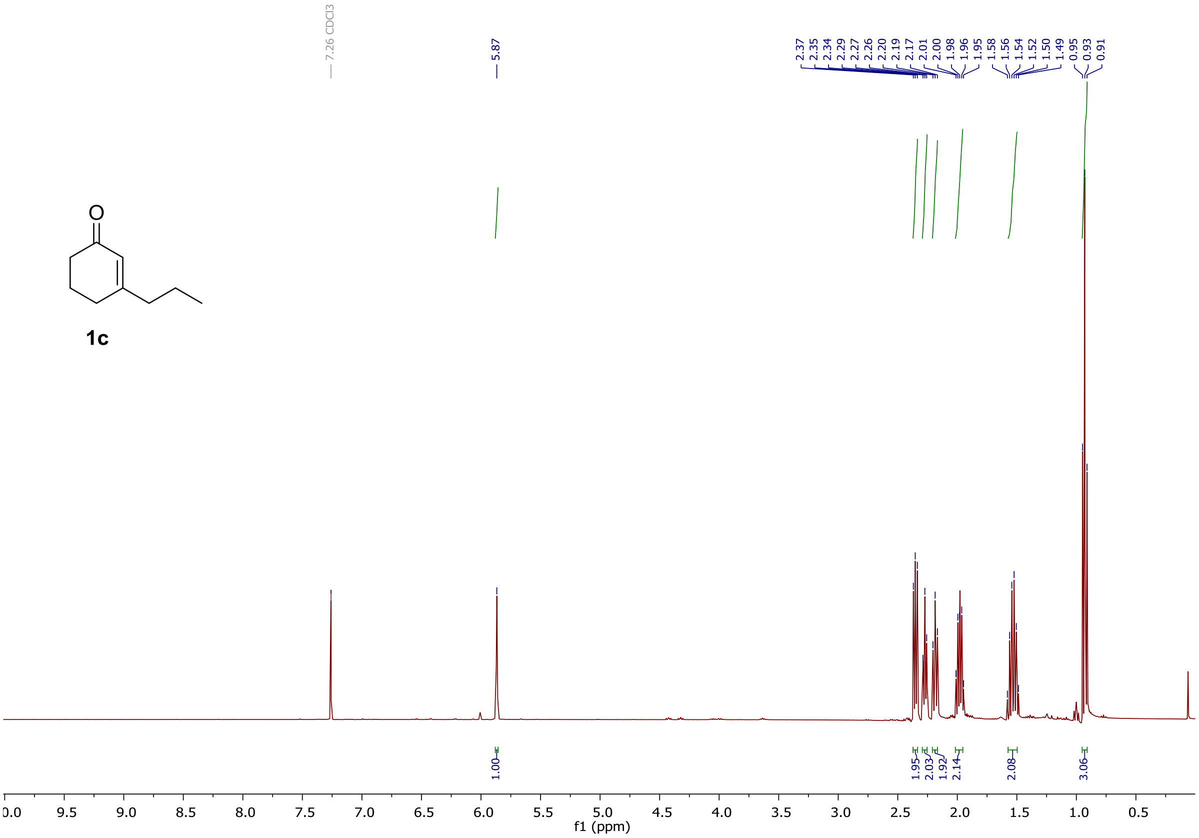

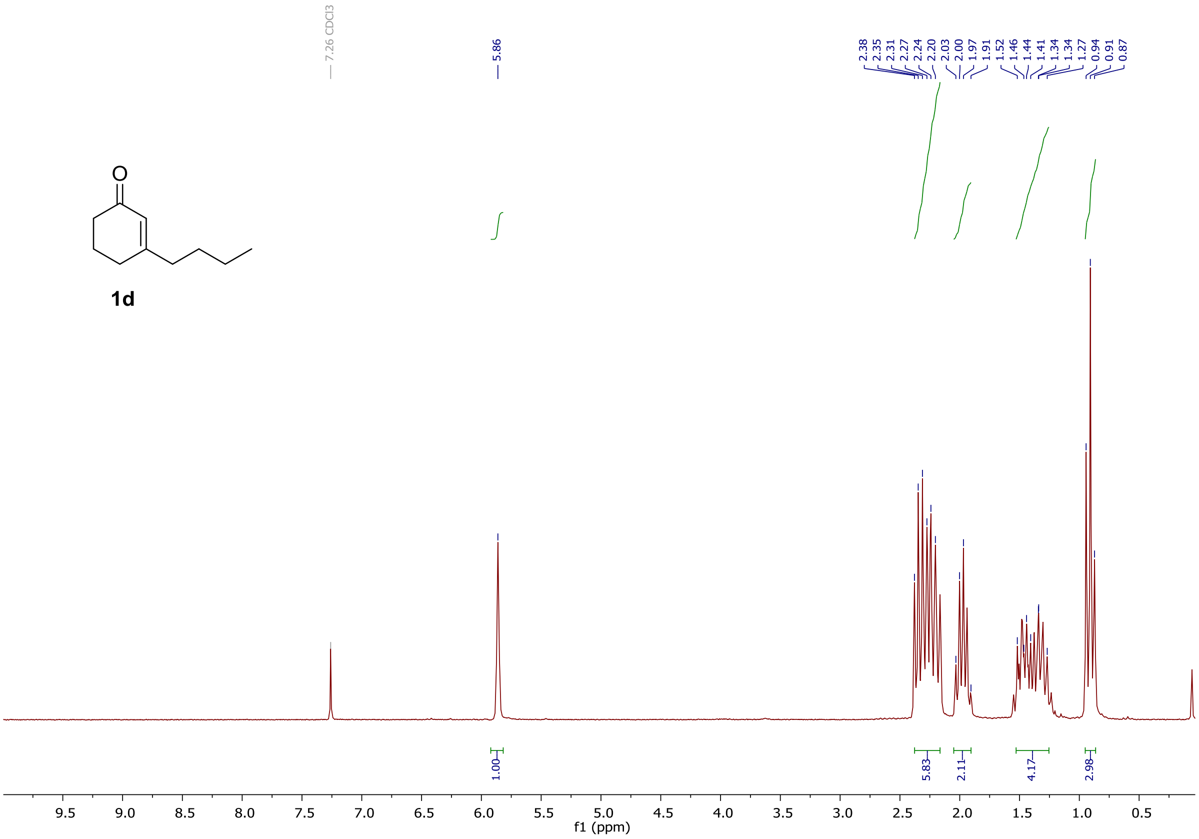

7.26 CDCl3
5.86
2.38
2.35
2.31
2.27
2.24
2.20
2.03
2.00
1.97
1.91
1.52
1.46
1.44
1.41
1.34
1.34
1.27
0.94
0.91
0.87
O
1d
1.00
5.83
2.11
4.17
2.98
9.5 9.0 8.5 8.0 7.5 7.0 6.5 6.0 5.5 5.0 4.5 4.0 3.5 3.0 2.5 2.0 1.5 1.0 0.5
f1 (ppm)

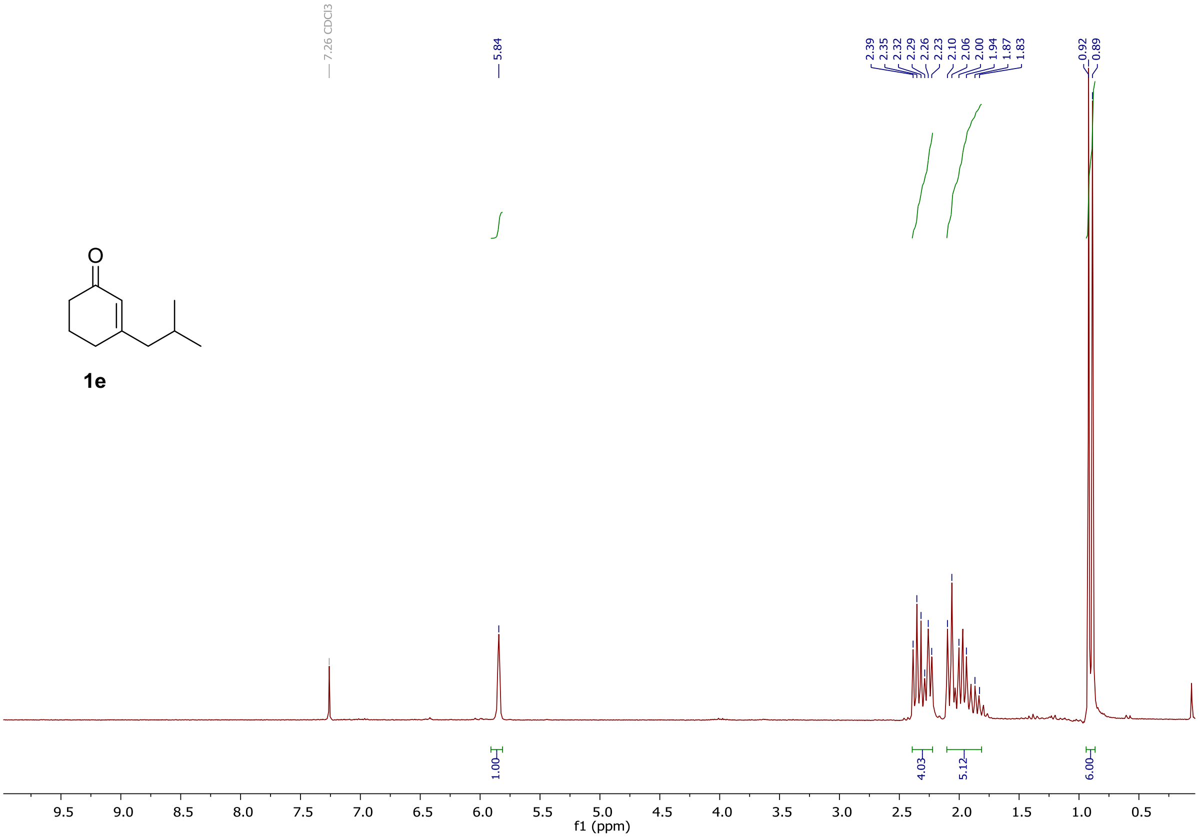

7.26 CDCl3
5.84
2.39
2.35
2.32
2.29
2.26
2.23
2.10
2.06
2.00
1.94
1.87
1.83
0.92
0.89
O
1e
1.00
4.03
5.12
6.00
9.5 9.0 8.5 8.0 7.5 7.0 6.5 6.0 5.5 5.0 4.5 4.0 3.5 3.0 2.5 2.0 1.5 1.0 0.5
f1 (ppm)

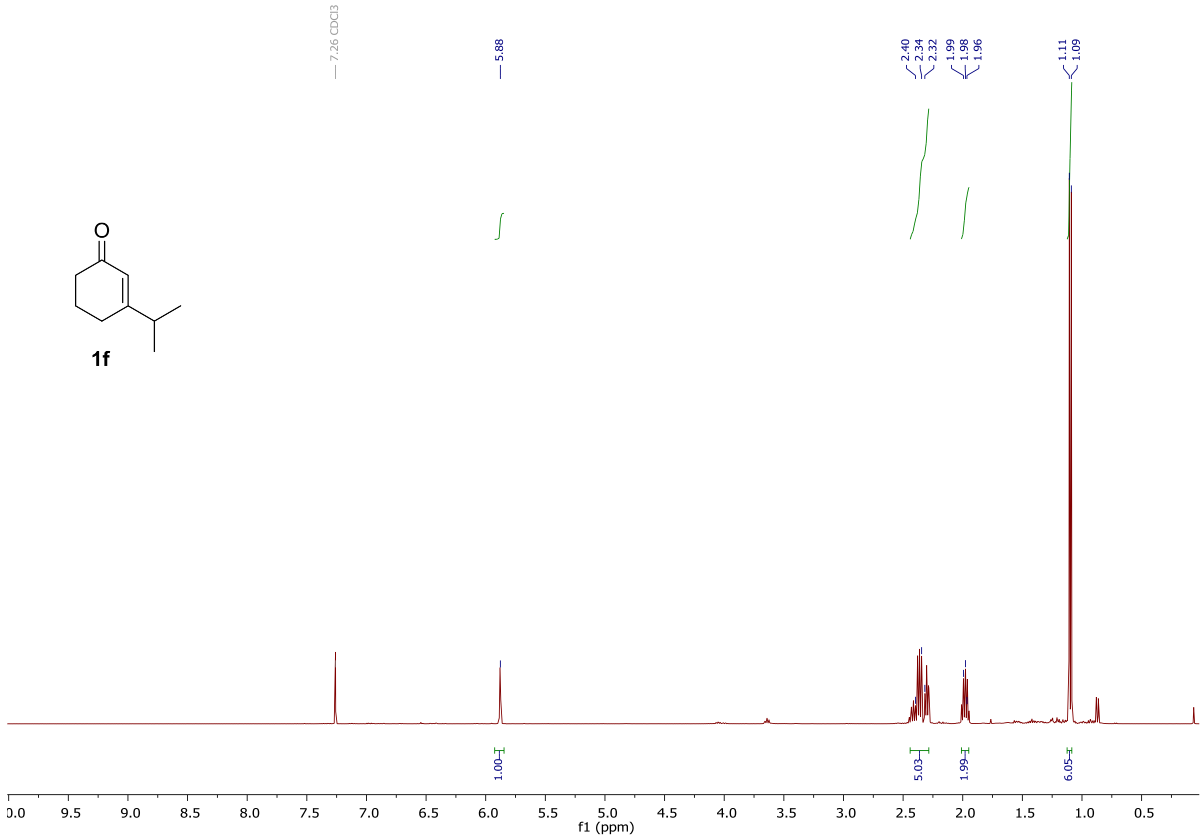
1f
7.26 CDCl3
5.88
2.40
2.34
2.32
1.99
1.98
1.96
1.11
1.09
1.00
5.03
1.99
6.05
f1 (ppm)

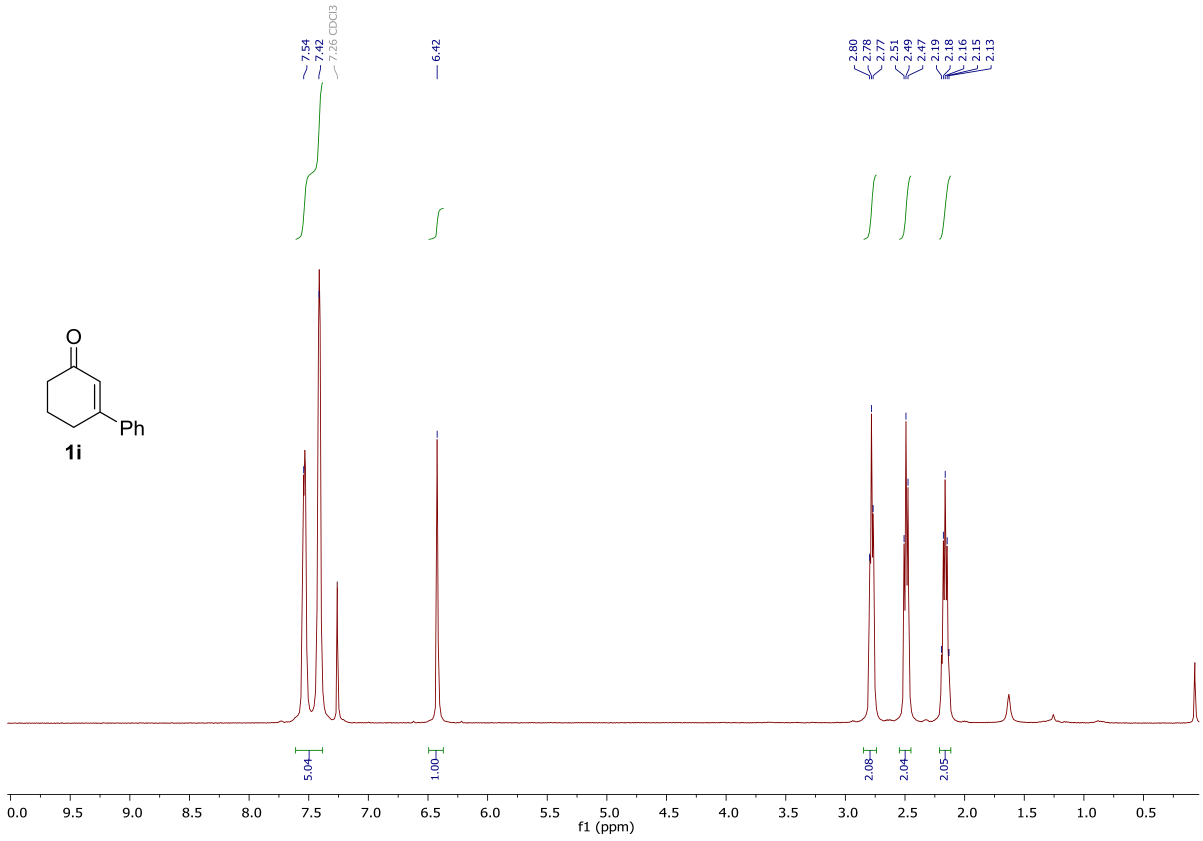
1i
Ph
7.54
7.42
7.26 CDCl3
6.42
2.80
2.78
2.77
2.51
2.49
2.47
2.19
2.18
2.16
2.15
2.13
5.04
1.00
2.08
2.04
2.05
f1 (ppm)

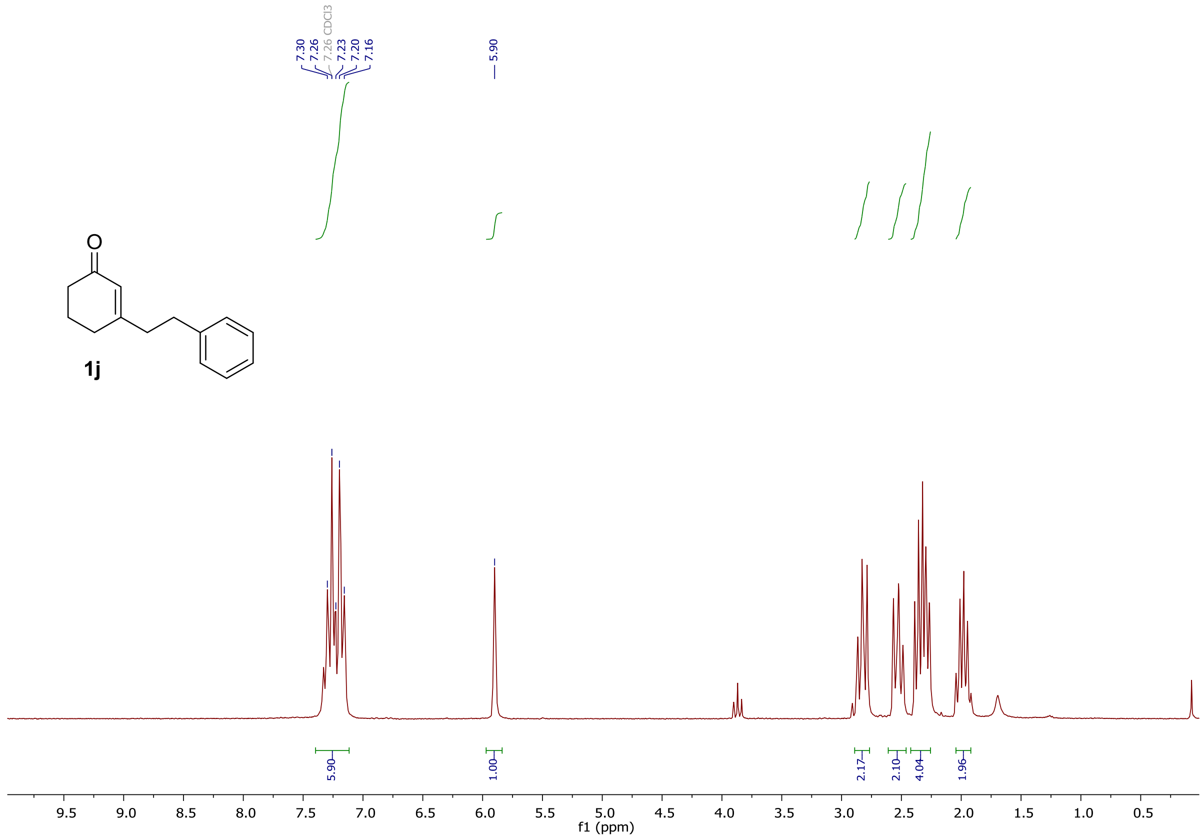
7.30
7.26
7.26 CDCl3
7.23
7.20
7.16
5.90
1j
5.90
1.00
2.17
2.10
4.04
1.96
9.5 9.0 8.5 8.0 7.5 7.0 6.5 6.0 5.5 5.0 4.5 4.0 3.5 3.0 2.5 2.0 1.5 1.0 0.5
f1 (ppm)

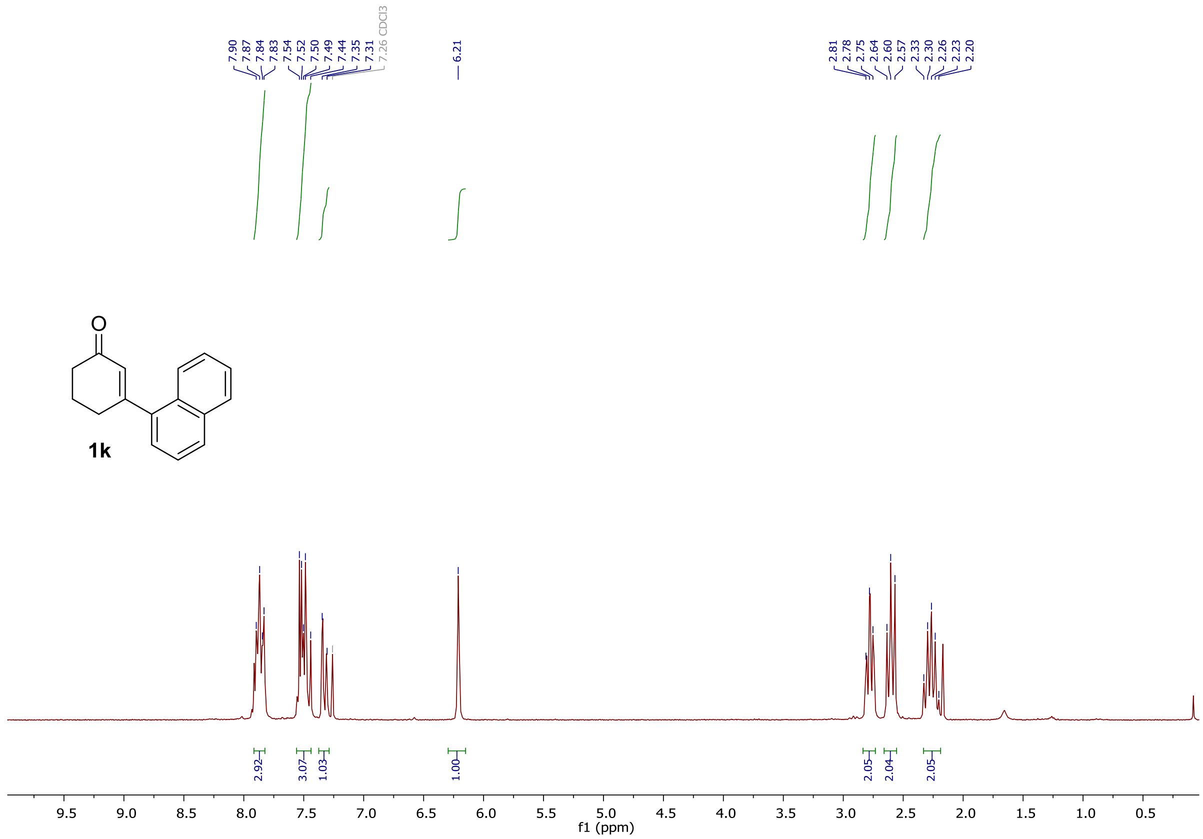
7.90
7.87
7.84
7.83
7.54
7.52
7.50
7.49
7.44
7.35
7.31
7.26 CDCl3
6.21
2.81
2.78
2.75
2.64
2.60
2.57
2.33
2.30
2.26
2.23
2.20
1k
2.92
3.07
1.03
1.00
2.05
2.04
2.05
9.5 9.0 8.5 8.0 7.5 7.0 6.5 6.0 5.5 5.0 4.5 4.0 3.5 3.0 2.5 2.0 1.5 1.0 0.5
f1 (ppm)

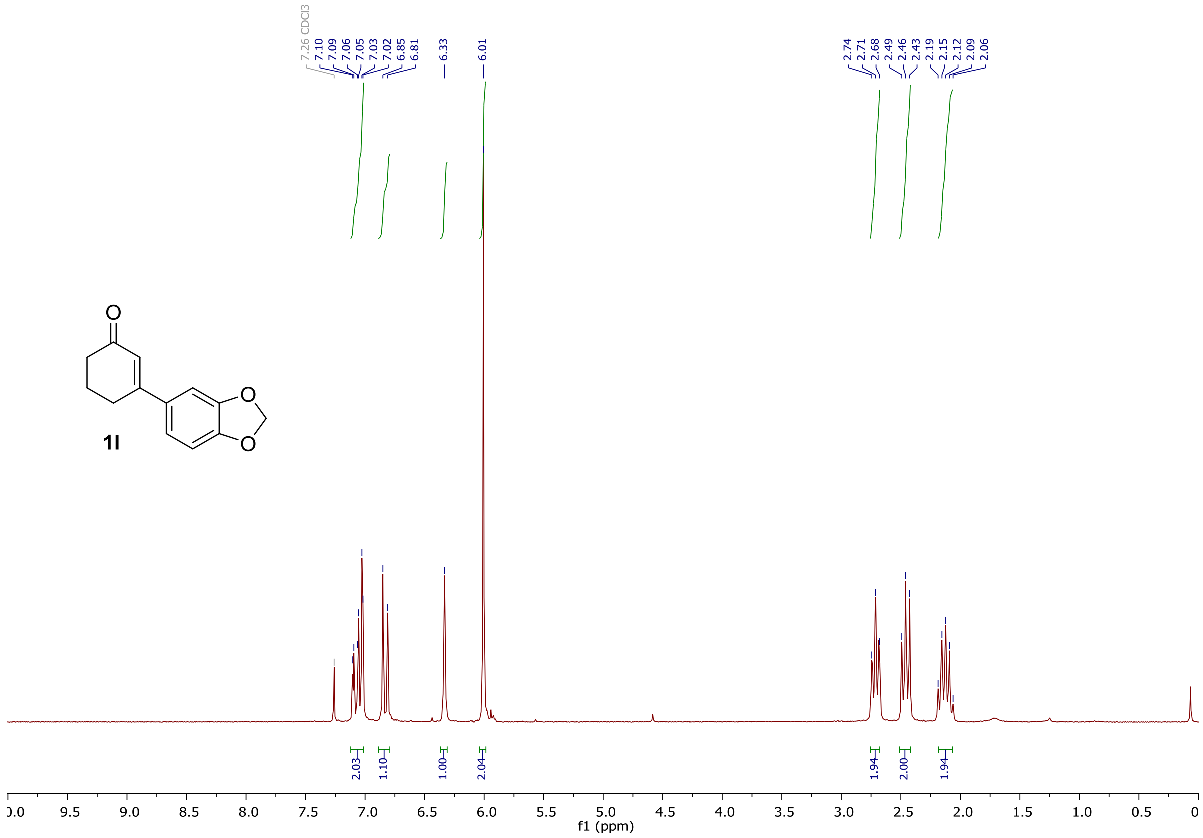
7.26 CDCl3
7.10
7.09
7.06
7.05
7.03
7.02
6.85
6.81
6.33
6.01
2.74
2.71
2.68
2.49
2.46
2.43
2.19
2.15
2.12
2.09
2.06
1l
2.03
1.10
1.00
2.04
1.94
2.00
1.94
f1 (ppm)

11. NMR specta of ATH products

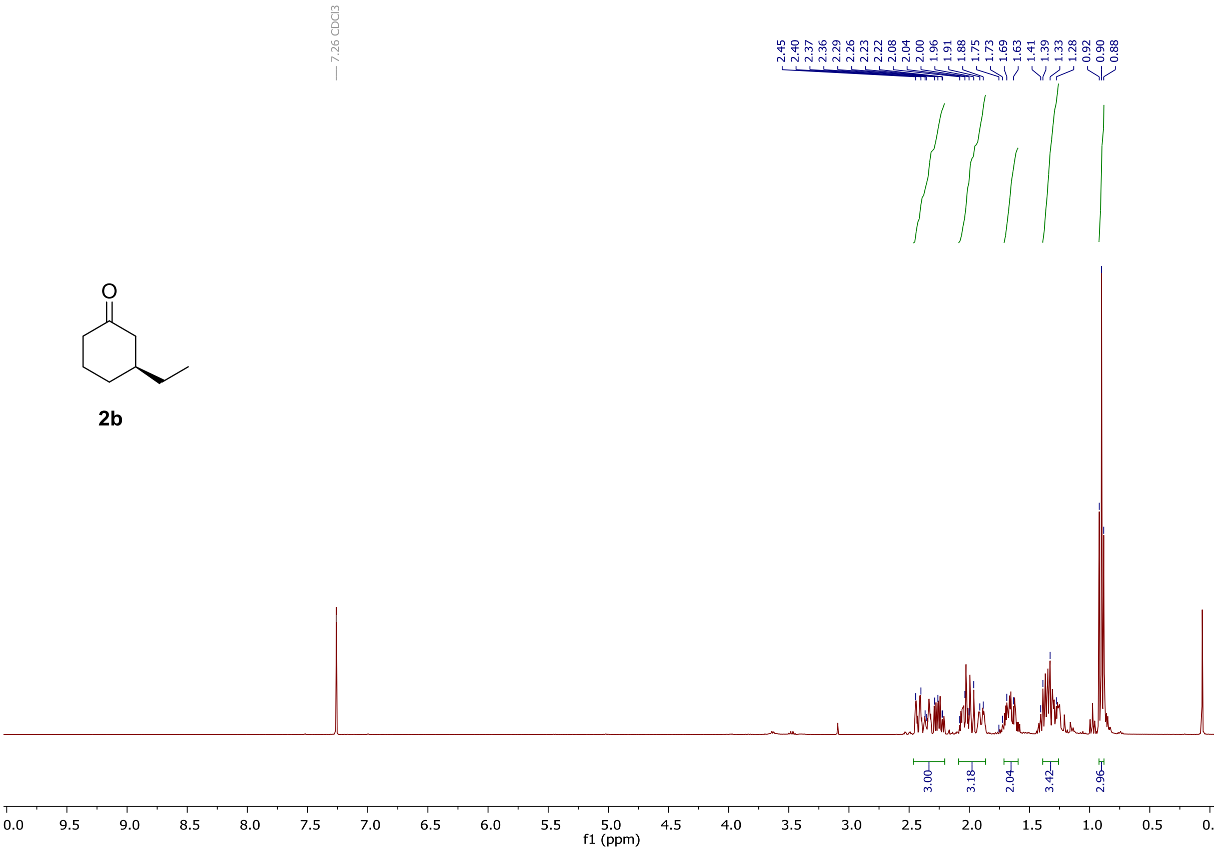

2b

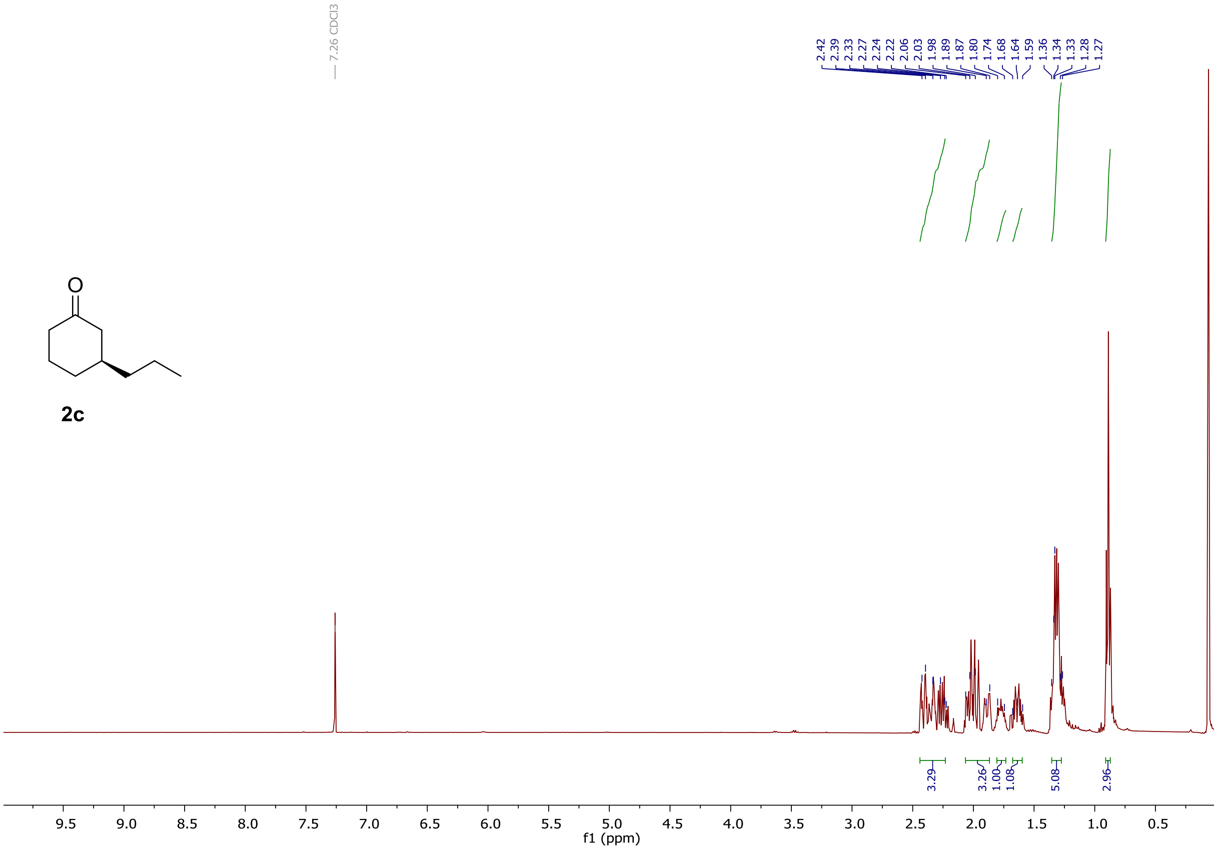

2c

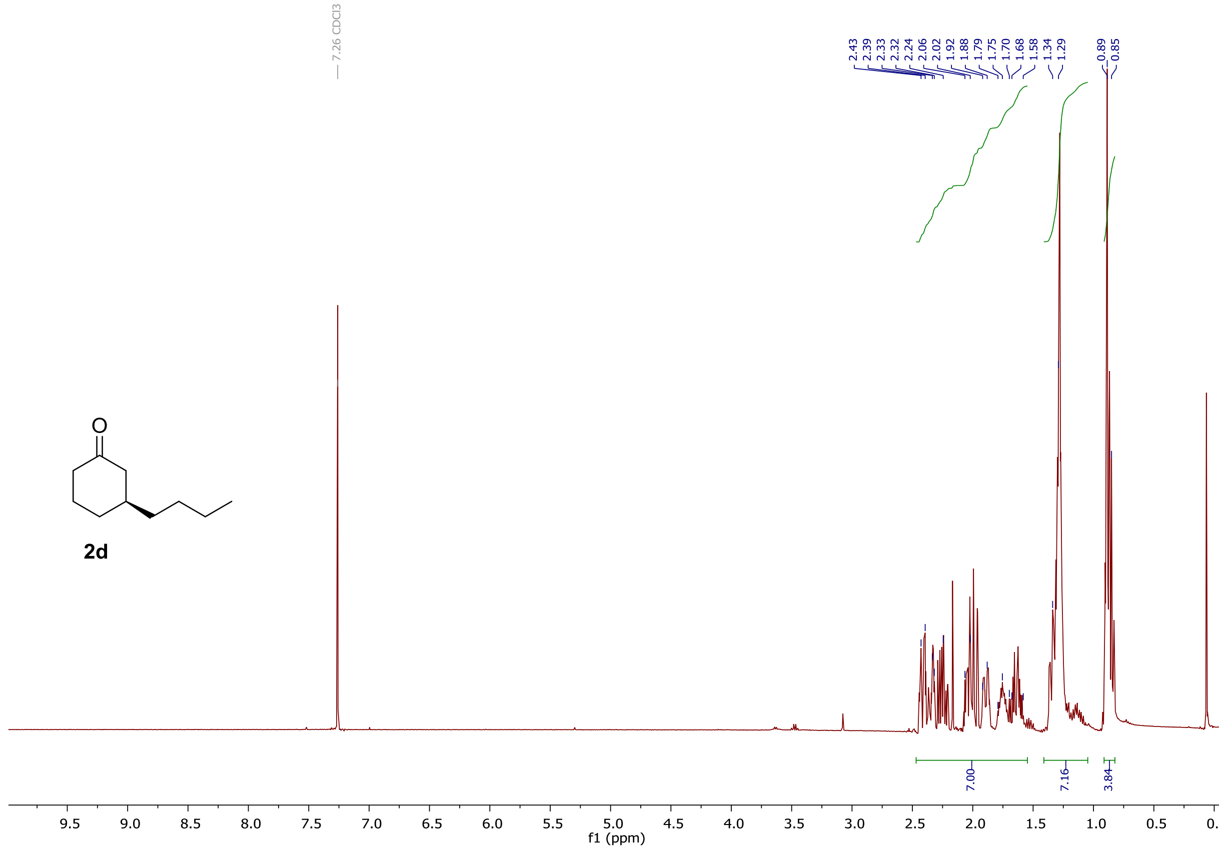
7.26 CDCl3
2.43 2.39 2.33 2.32 2.24 2.06 2.02 1.92 1.88 1.79 1.75 1.70 1.68 1.58 1.34 1.29
0.89 0.85
2d
7.00
7.16
3.84
9.5 9.0 8.5 8.0 7.5 7.0 6.5 6.0 5.5 5.0 4.5 4.0 3.5 3.0 2.5 2.0 1.5 1.0 0.5 0.
f1 (ppm)

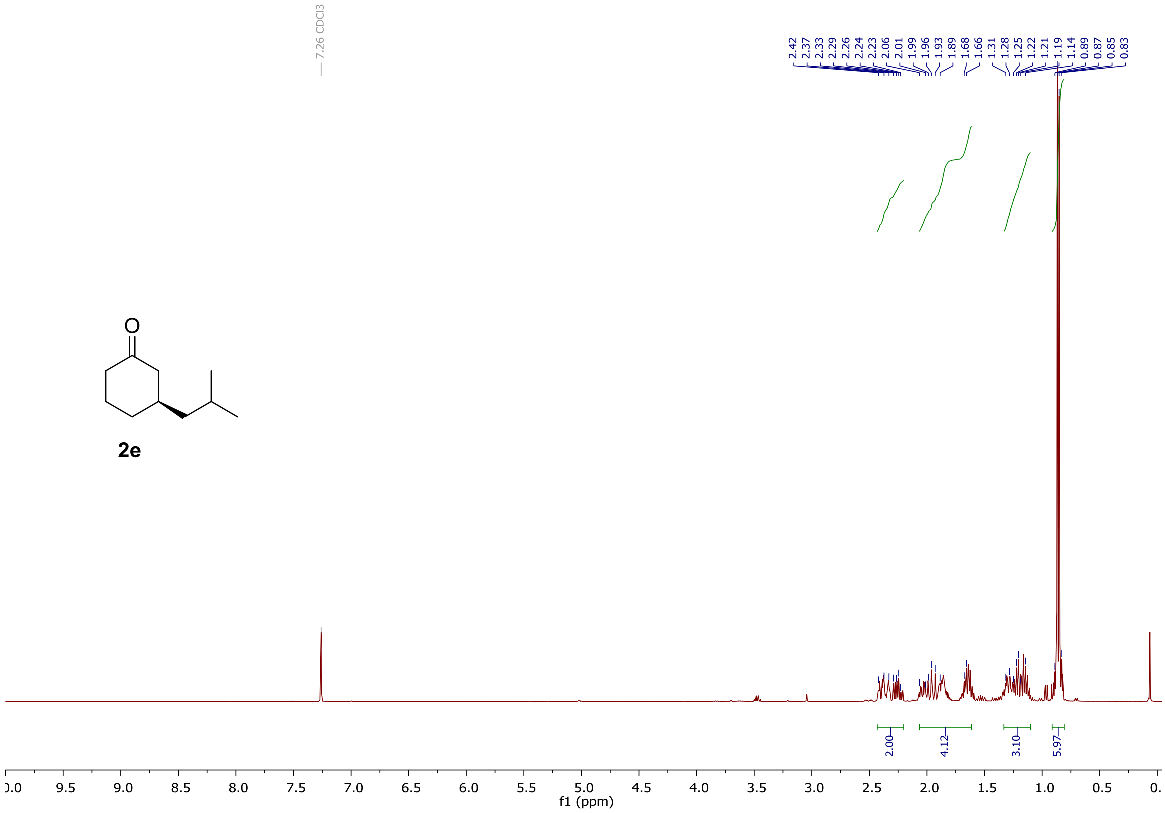
7.26 CDCl3
2.42 2.37 2.33 2.29 2.26 2.24 2.23 2.06 2.01 1.99 1.96 1.93 1.89 1.68 1.66 1.31 1.28 1.25 1.22 1.21 1.19 1.14 0.89 0.87 0.85 0.83
2e
2.00
4.12
3.10
5.97
).0 9.5 9.0 8.5 8.0 7.5 7.0 6.5 6.0 5.5 5.0 4.5 4.0 3.5 3.0 2.5 2.0 1.5 1.0 0.5 0.
f1 (ppm)

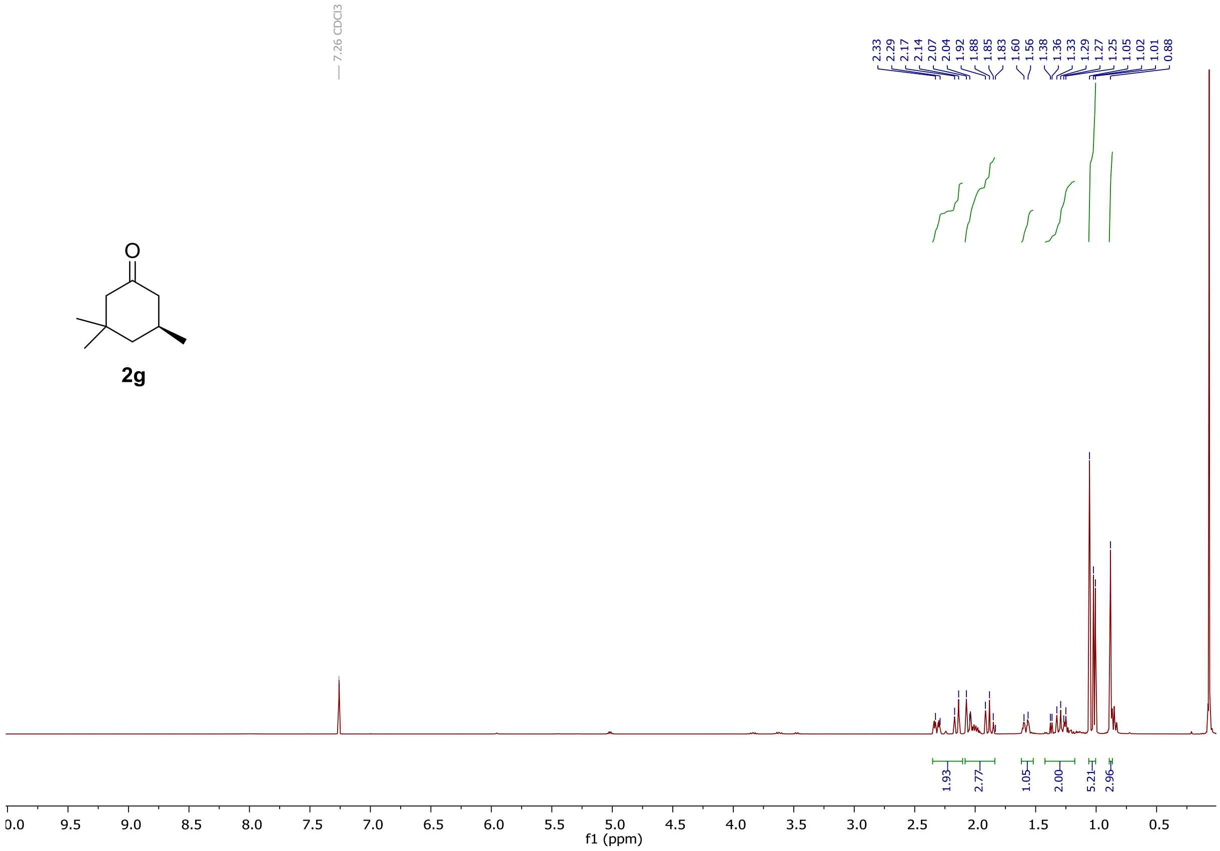
7.26 CDCl3
2.33 2.29 2.17 2.14 2.07 2.04 1.92 1.88 1.85 1.83 1.60 1.56 1.38 1.36 1.33 1.29 1.27 1.25 1.05 1.02 1.01 0.88
O
2g
1.93 2.77 1.05 2.00 5.21 2.96
10.0 9.5 9.0 8.5 8.0 7.5 7.0 6.5 6.0 5.5 5.0 4.5 4.0 3.5 3.0 2.5 2.0 1.5 1.0 0.5
f1 (ppm)

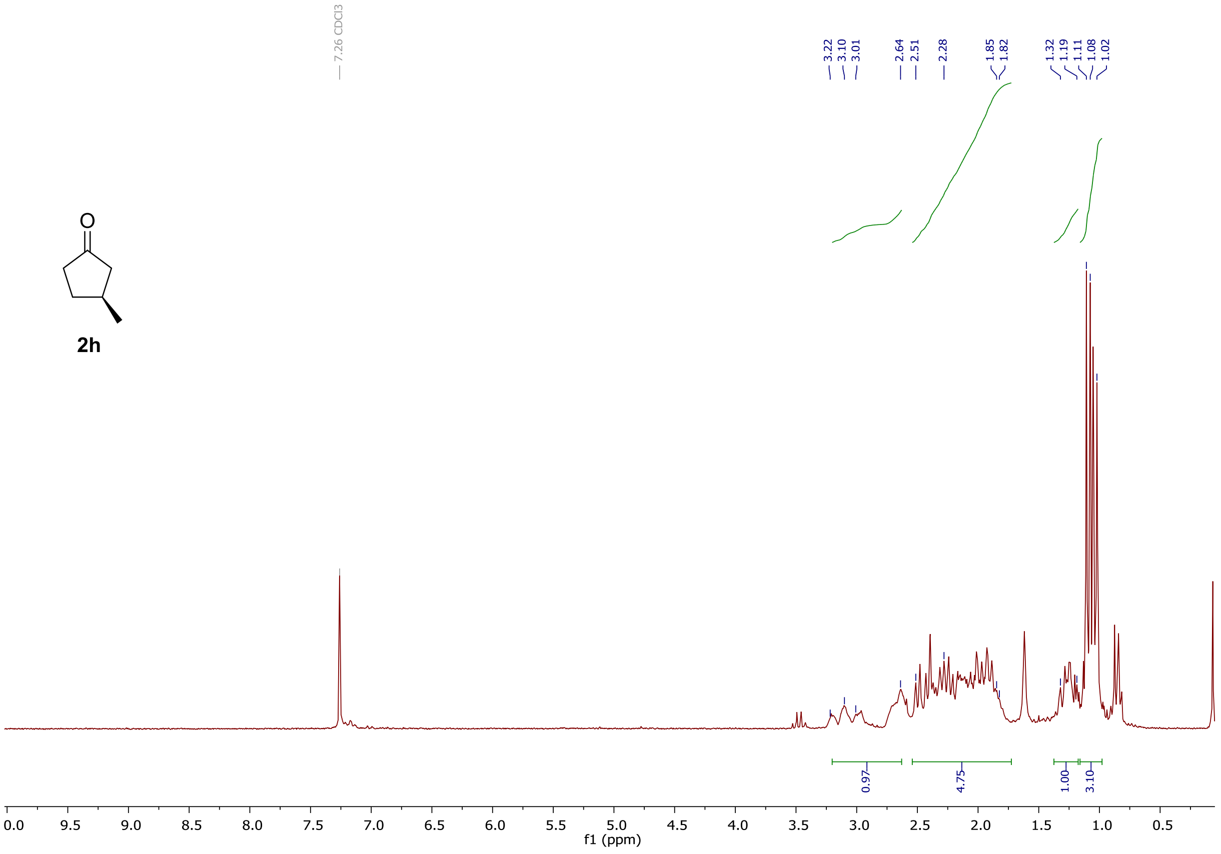
7.26 CDCl3
3.22 3.10 3.01 2.64 2.51 2.28 1.85 1.82 1.32 1.19 1.11 1.08 1.02
O
2h
0.97 4.75 1.00 3.10
10.0 9.5 9.0 8.5 8.0 7.5 7.0 6.5 6.0 5.5 5.0 4.5 4.0 3.5 3.0 2.5 2.0 1.5 1.0 0.5
f1 (ppm)

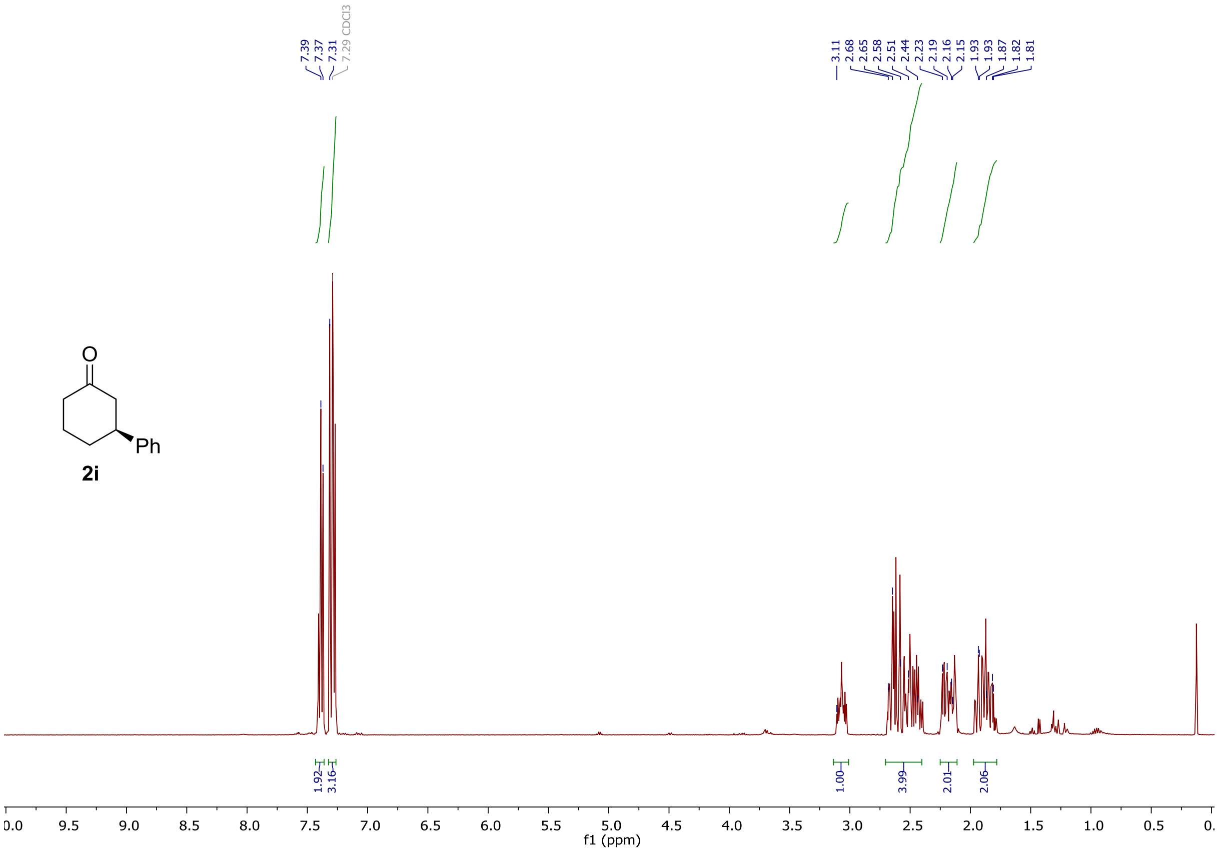
7.39
7.37
7.31
7.29 CDCl3
3.11
2.68
2.65
2.58
2.51
2.44
2.23
2.19
2.16
2.15
1.93
1.93
1.87
1.82
1.81
O
Ph
1.92
3.16
1.00
3.99
2.01
2.06
f1 (ppm)

2i

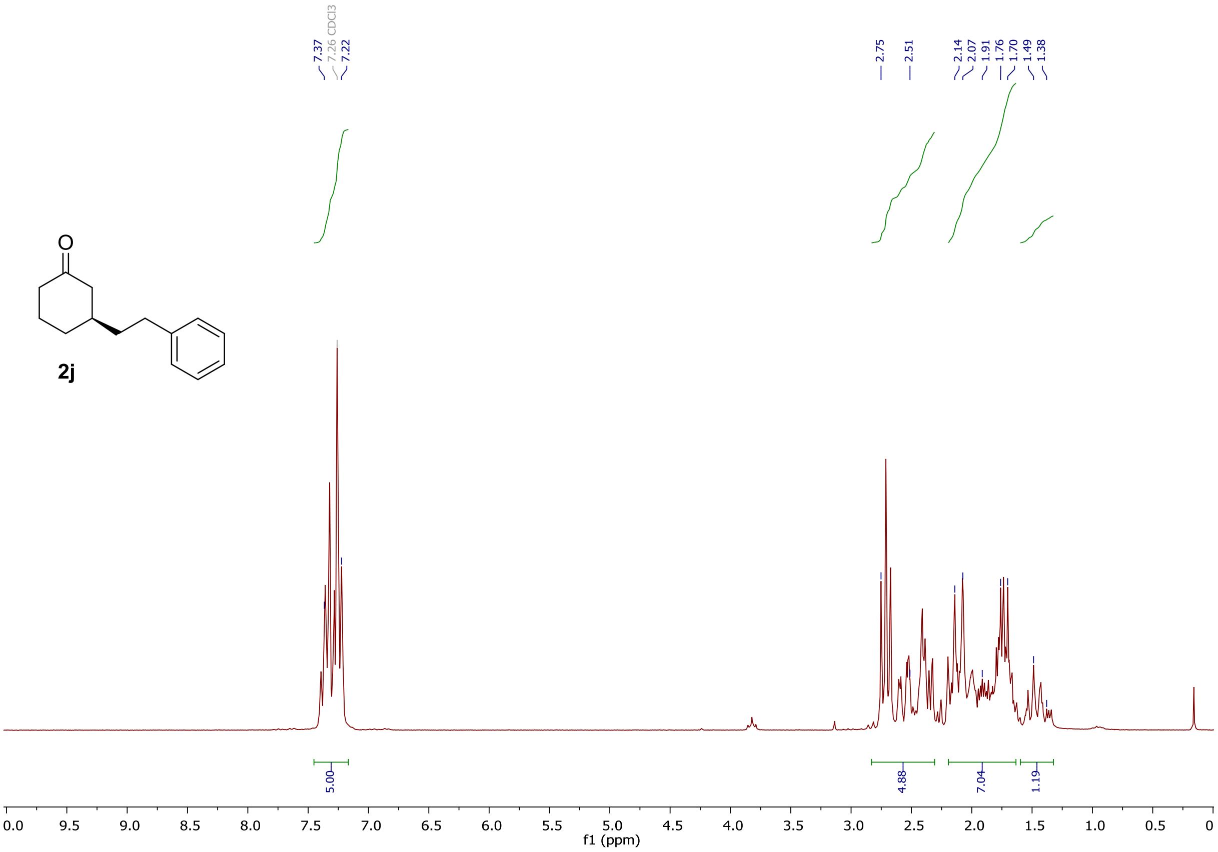
7.37
7.26 CDCl3
7.22
2.75
2.51
2.14
2.07
1.91
1.76
1.70
1.49
1.38
O
5.00
4.88
7.04
1.19
f1 (ppm)

2j

naphtyl

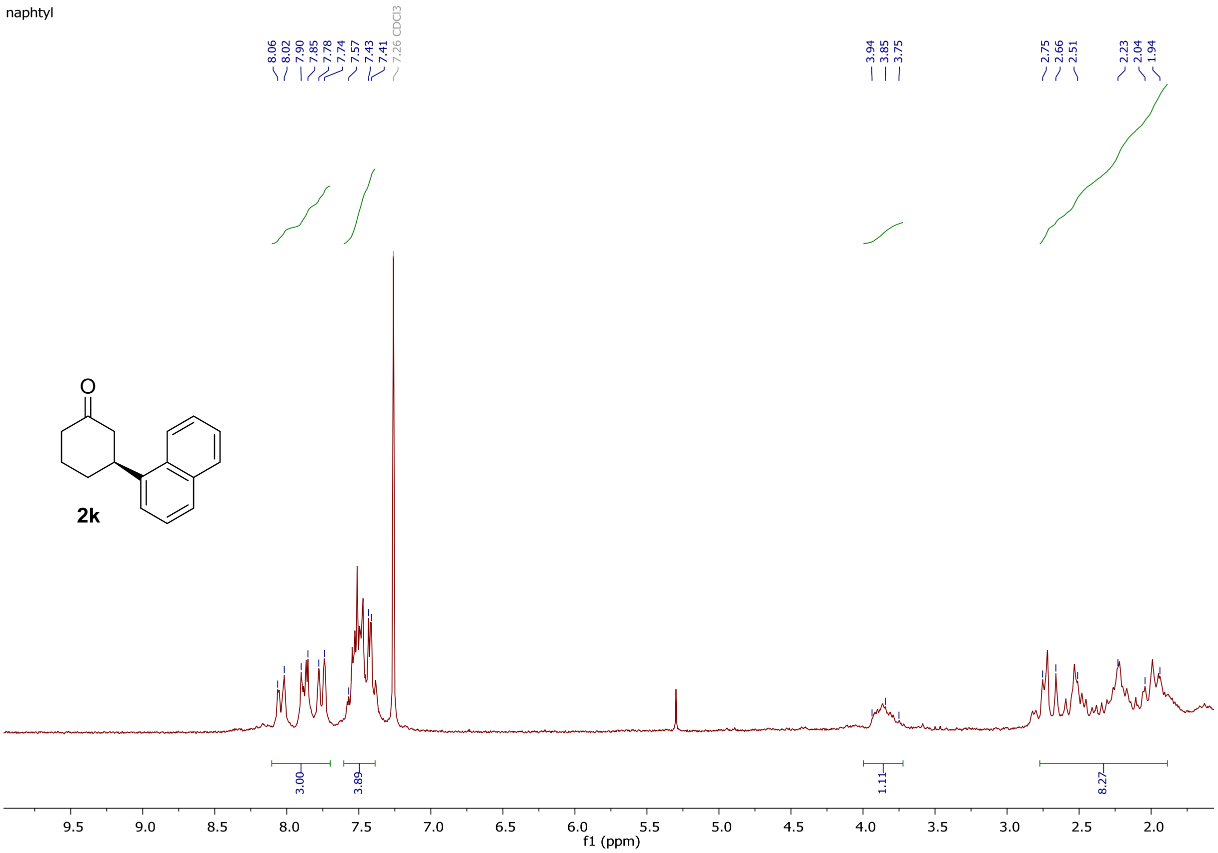
8.06
8.02
7.90
7.85
7.78
7.74
7.57
7.43
7.41
7.26 CDCl3
3.94
3.85
3.75
2.75
2.66
2.51
2.23
2.04
1.94
O
2k
3.00
3.89
1.11
8.27
9.5 9.0 8.5 8.0 7.5 7.0 6.5 6.0 5.5 5.0 4.5 4.0 3.5 3.0 2.5 2.0
f1 (ppm)

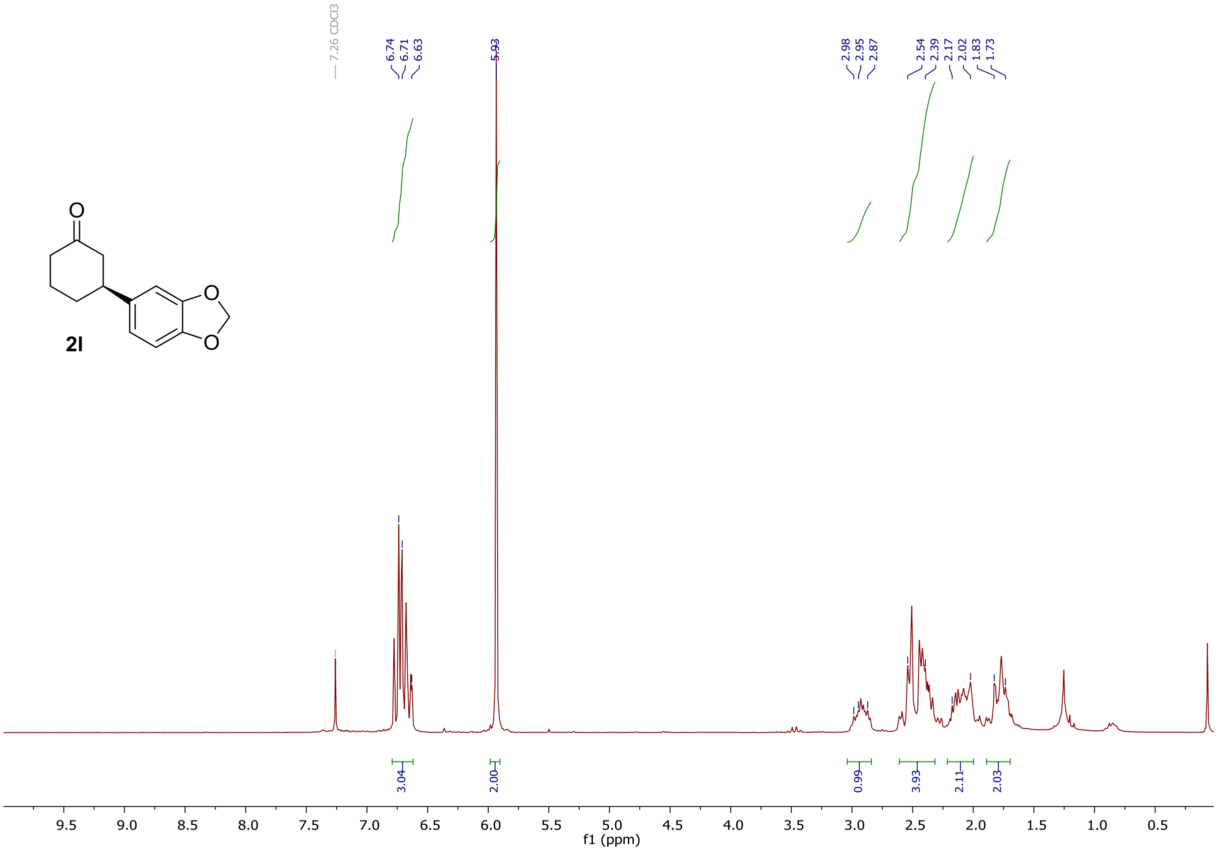
7.26 CDCl3
6.74
6.71
6.63
5.93
2.98
2.95
2.87
2.54
2.39
2.17
2.02
1.83
1.73
O
O
O
2l
3.04
2.00
0.99
3.93
2.11
2.03
9.5 9.0 8.5 8.0 7.5 7.0 6.5 6.0 5.5 5.0 4.5 4.0 3.5 3.0 2.5 2.0 1.5 1.0 0.5
f1 (ppm)

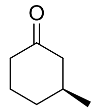

2a

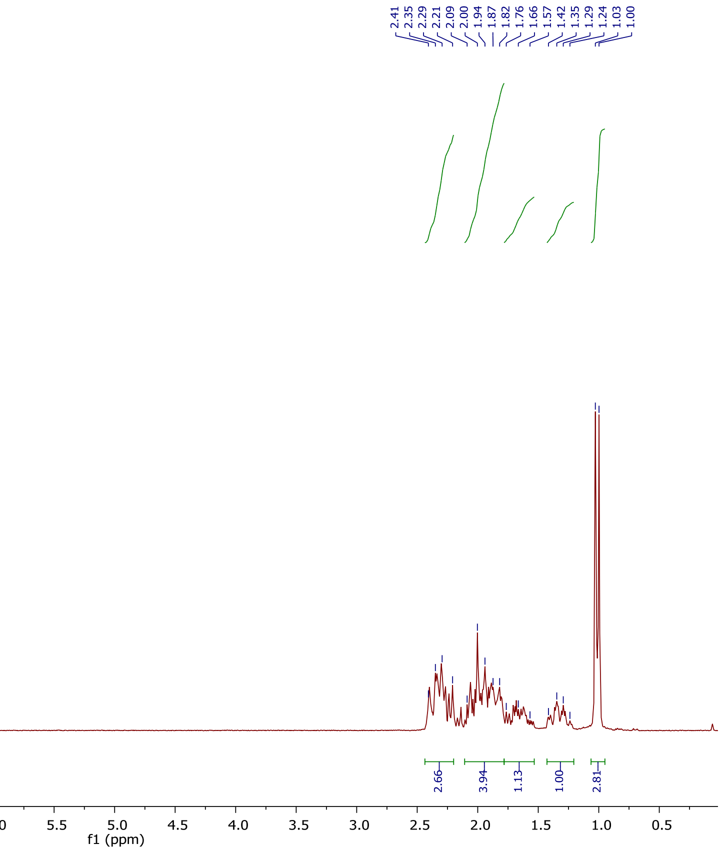
2.41
2.35
2.29
2.21
2.09
2.00
1.94
1.87
1.82
1.76
1.66
1.57
1.42
1.35
1.29
1.24
1.03
1.00
2.66
3.94
1.13
1.00
2.81
0.0
9.5
9.0
8.5
8.0
7.5
7.0
6.5
6.0
5.5
5.0
4.5
4.0
3.5
3.0
2.5
2.0
1.5
1.0
0.5
f1 (ppm)

12. Chiral GC and HPLC traces

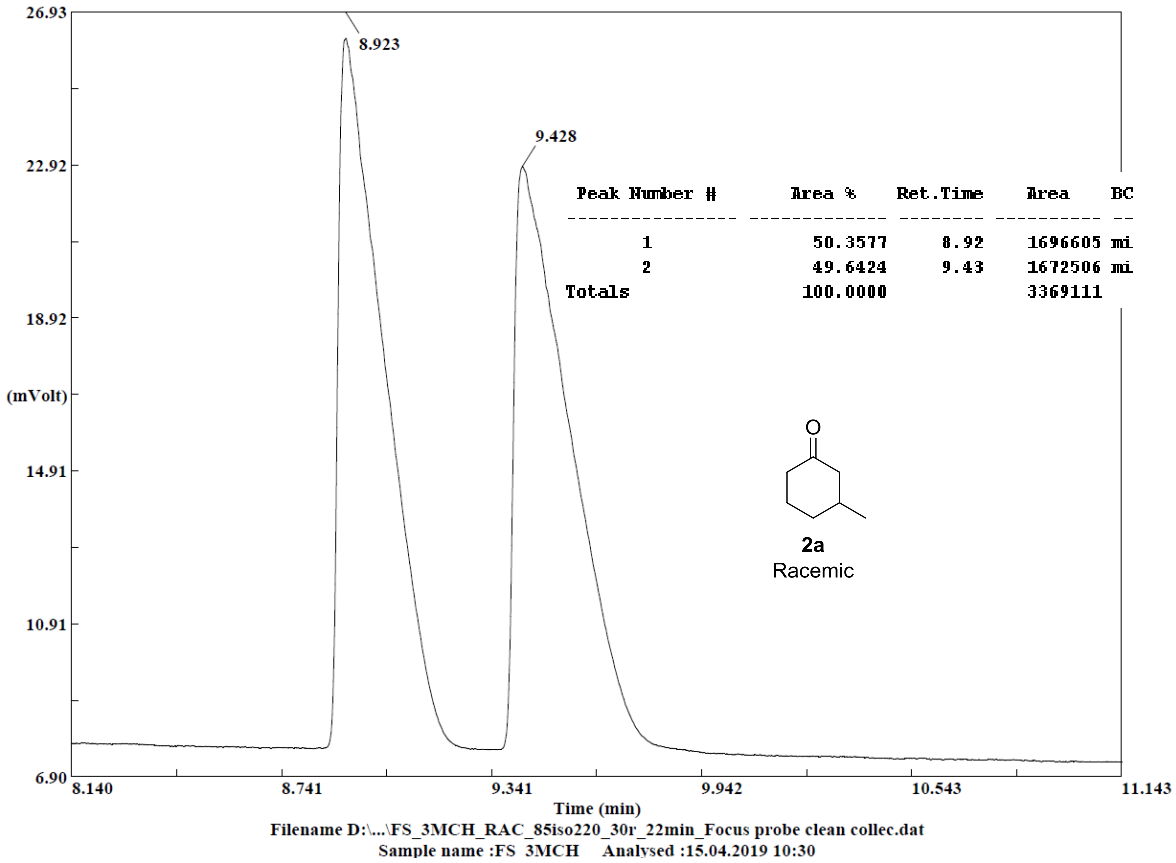

Peak Number #	Area %	Ret.Time	Area	BC
1	50.3577	8.92	1696605	mi
2	49.6424	9.43	1672506	mi
Totals	100.0000		3369111	

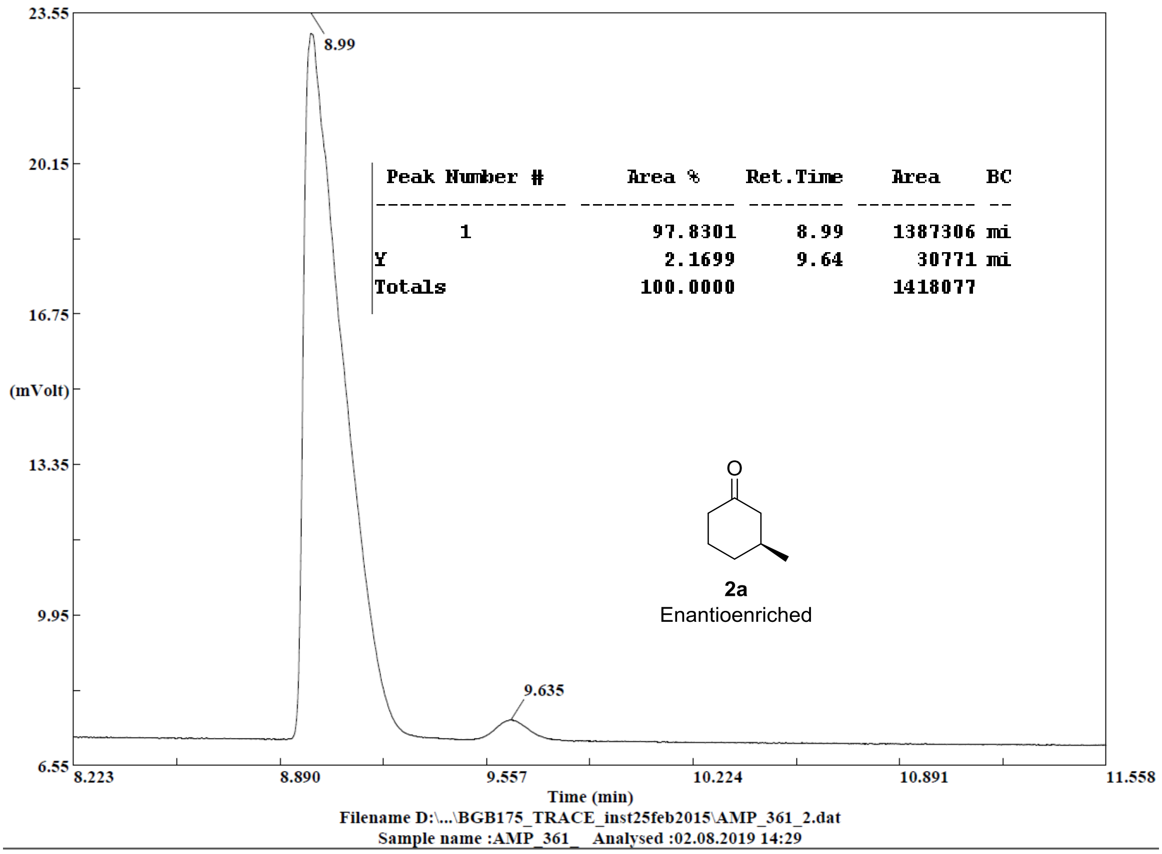

Peak Number #	Area %	Ret.Time	Area	BC
1	97.8301	8.99	1387306	mi
Y	2.1699	9.64	30771	mi
Totals	100.0000		1418077	

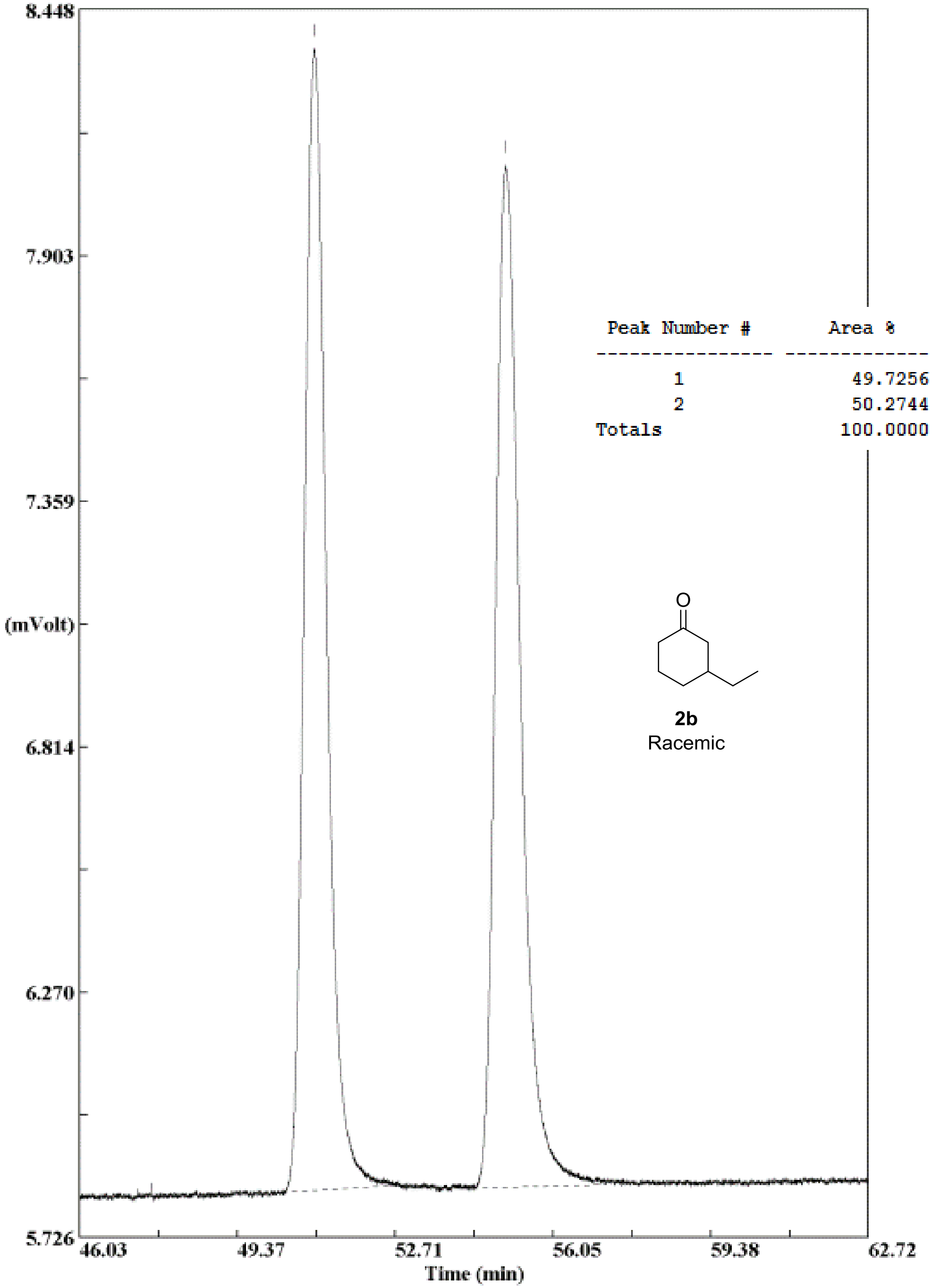

Peak Number #	Area %	Ret.Time	Area	BC
1	49.7256	50.99	828902	mi
2	50.2744	55.04	838049	mi
Totals	100.0000		1666951	

2b
Racemic

Filename C:\...\FS_177_3ECH_old_52iso_121min.dat
Sample name :FS_177_3ECH_old_52iso_121min Analysed :29.04.2019 11:08

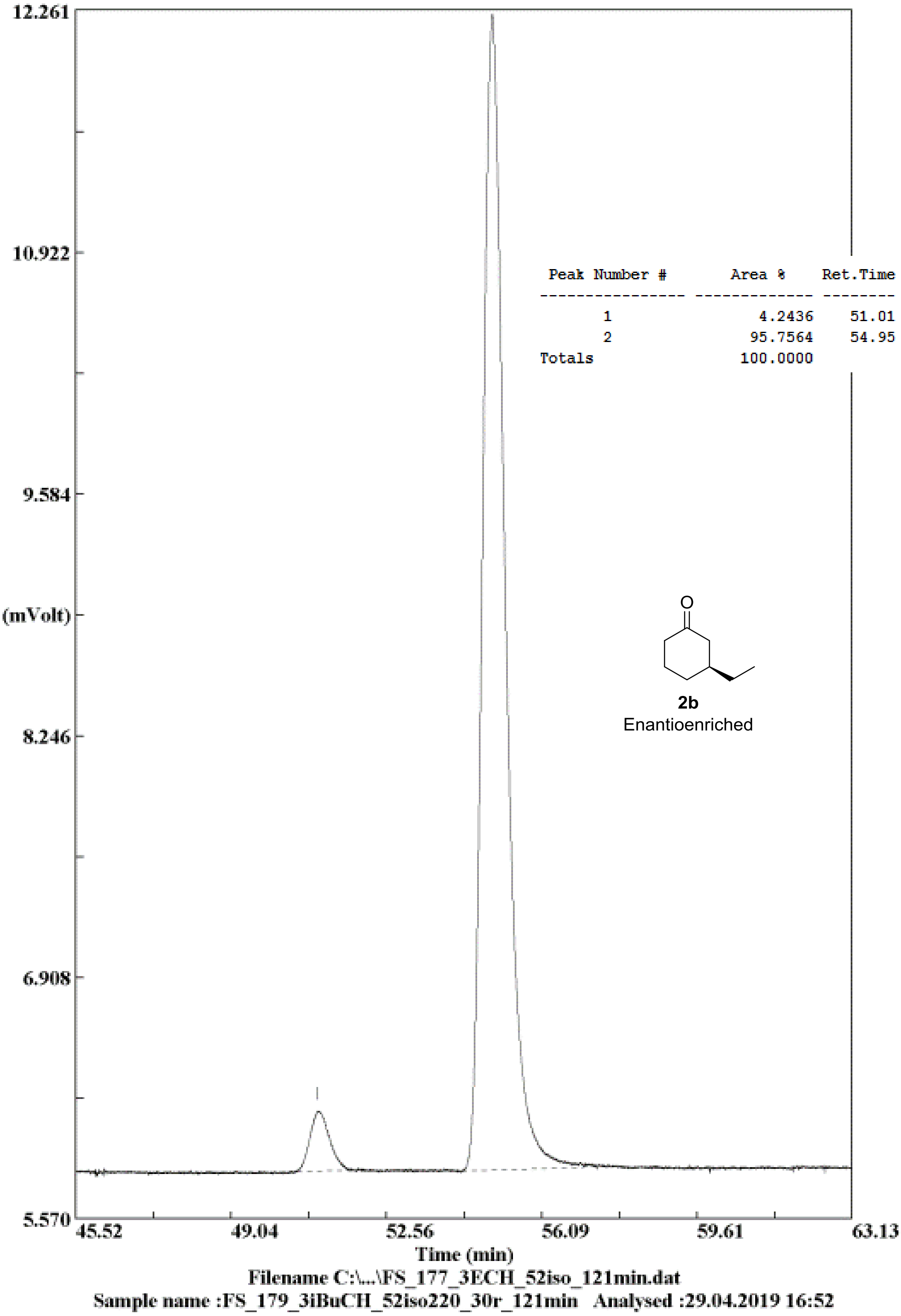

Peak Number #	Area %	Ret.Time	Area	BC
1	4.2436	51.01	101040	mi
2	95.7564	54.95	2279946	mi
Totals	100.0000		2380986	

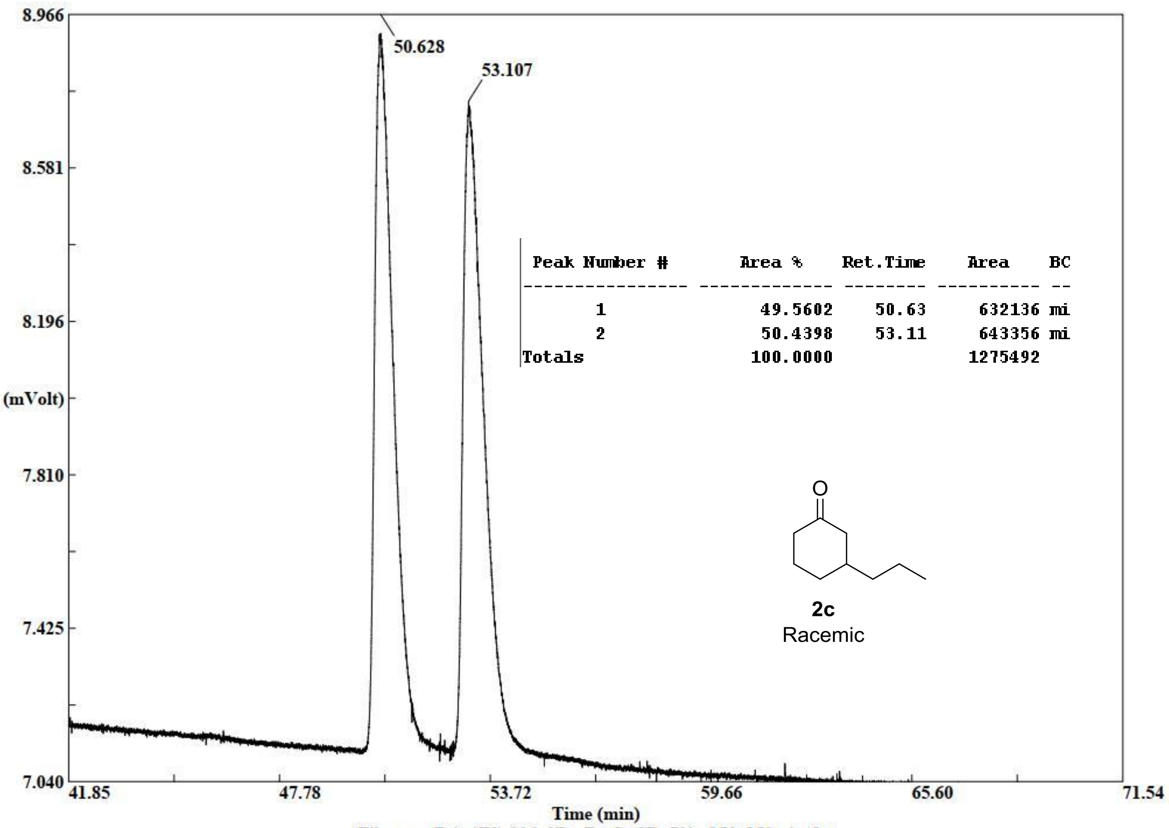

Peak Number #	Area %	Ret.Time	Area	BC
1	49.5602	50.63	632136	mi
2	50.4398	53.11	643356	mi
Totals	100.0000		1275492	

Filename D:\...\FS_206_3Pr_RAC_CR_70iso150_152min.dat
Sample name :FS_206_3 Analysed :29.05.2019 12:06

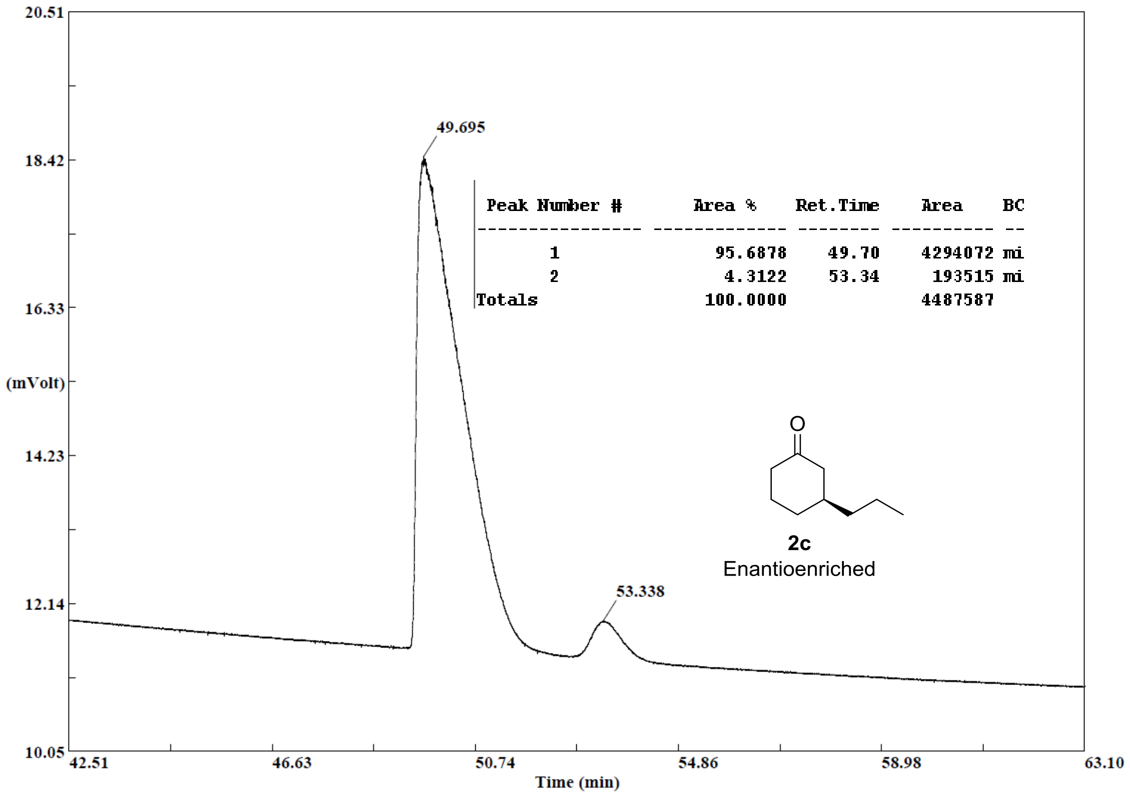

Peak Number #	Area %	Ret.Time	Area	BC
1	95.6878	49.70	4294072	mi
2	4.3122	53.34	193515	mi
Totals	100.0000		4487587	

Filename D:\...\FS_207_P_70iso150_1r_152min.dat
Sample name :FS_207_P Analysed :09.07.2019 13:02

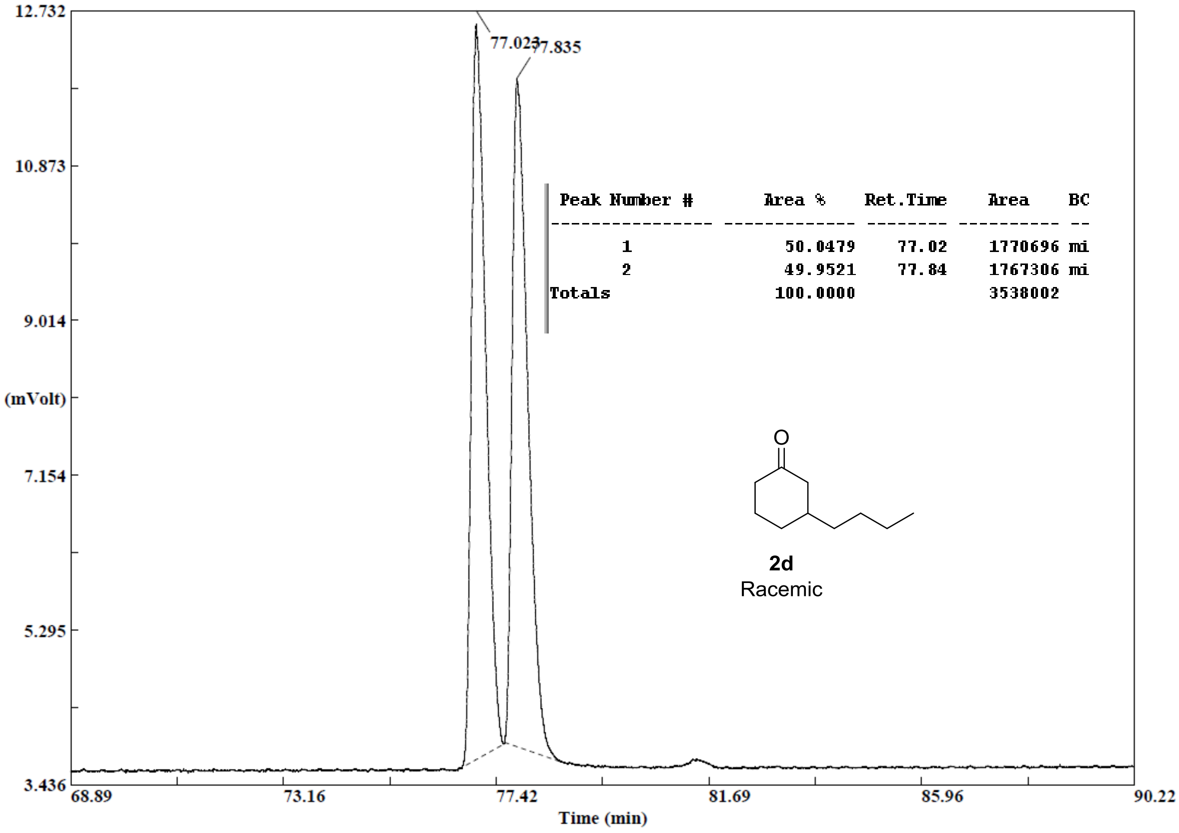

Peak Number #	Area %	Ret.Time	Area	BC
1	50.0479	77.02	1770696	mi
2	49.9521	77.84	1767306	mi
Totals	100.0000		3538002	

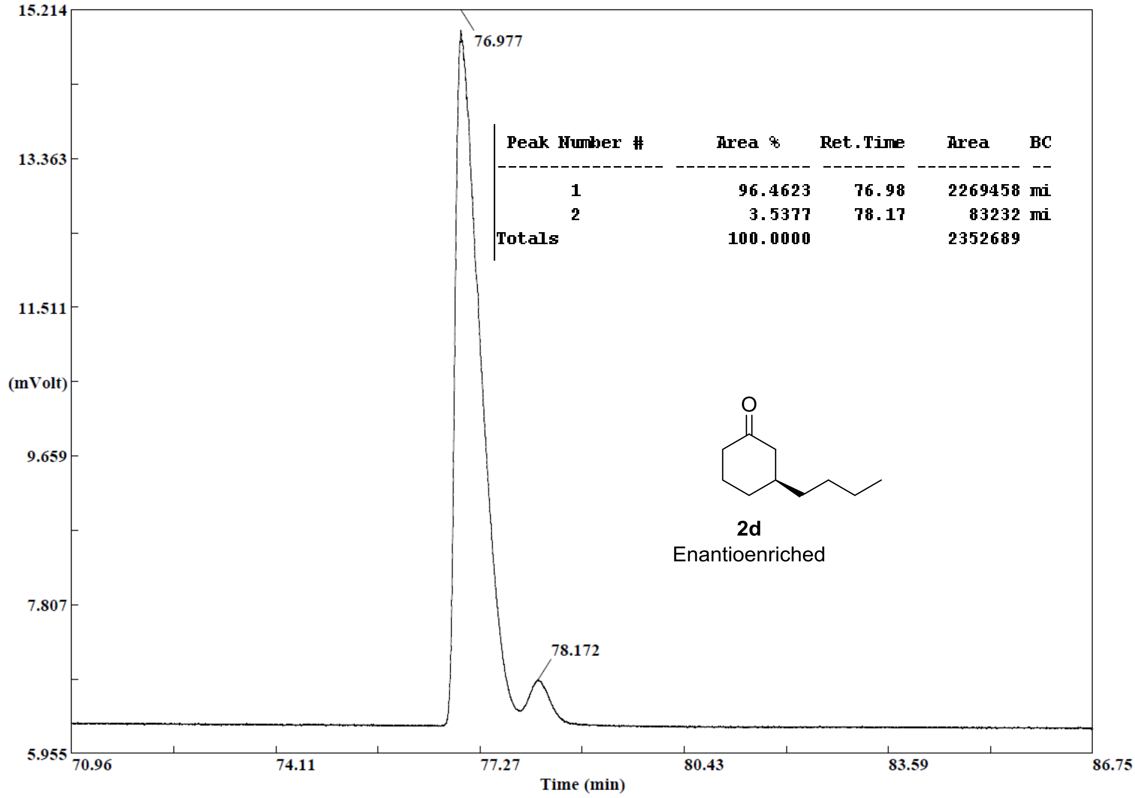

Peak Number #	Area %	Ret.Time	Area	BC
1	96.4623	76.98	2269458	mi
2	3.5377	78.17	83232	mi
Totals	100.0000		2352689	

Filename D:\...\FS_181_P_3BuCH_70iso150_1r_220_30r_152min.dat
Sample name :FS_181_P Analysed :29.04.2019 16:56

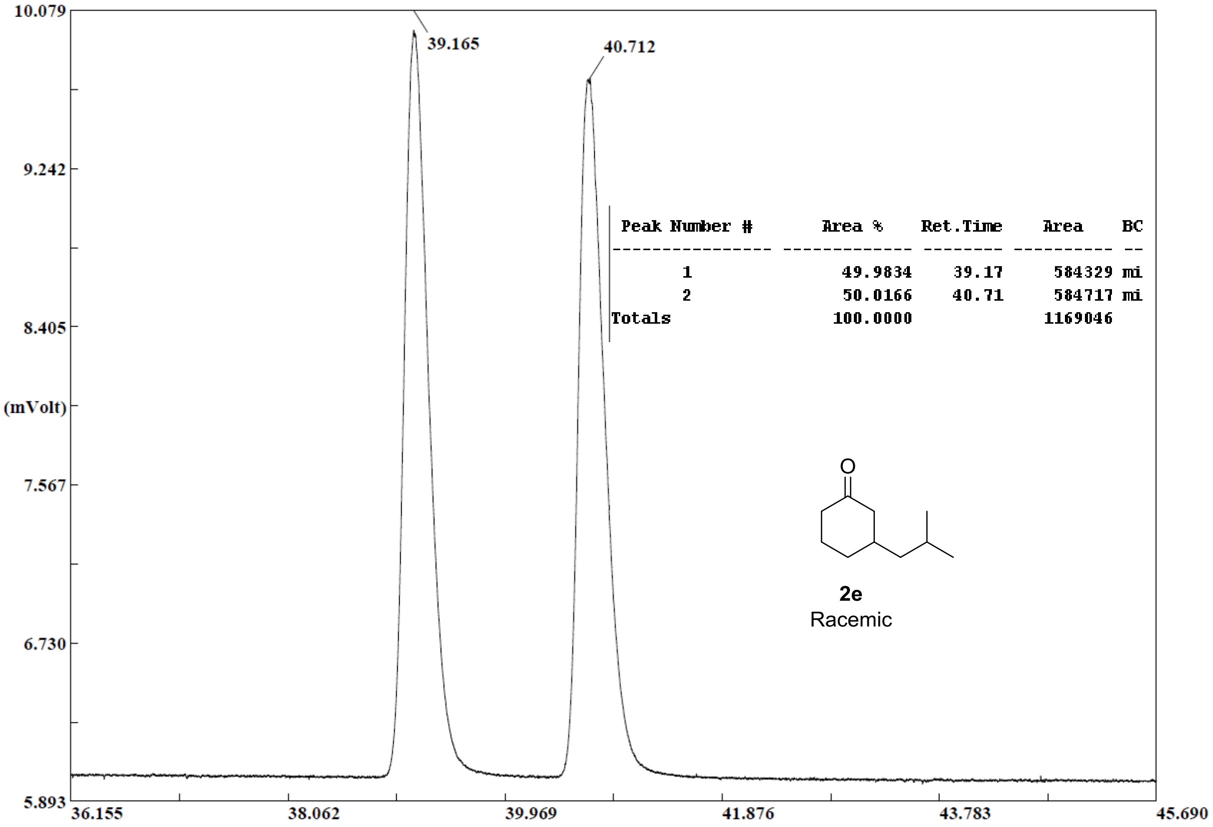

Peak Number #	Area %	Ret.Time	Area	BC
1	49.9834	39.17	584329	mi
2	50.0166	40.71	584717	mi
Totals	100.0000		1169046	

2e
Racemic

Filename D:\...\FS_186_3iBuCH_RAC_70_110_05r_99min.dat
Sample name :FS_186_3   Analysed :29.04.2019 20:02

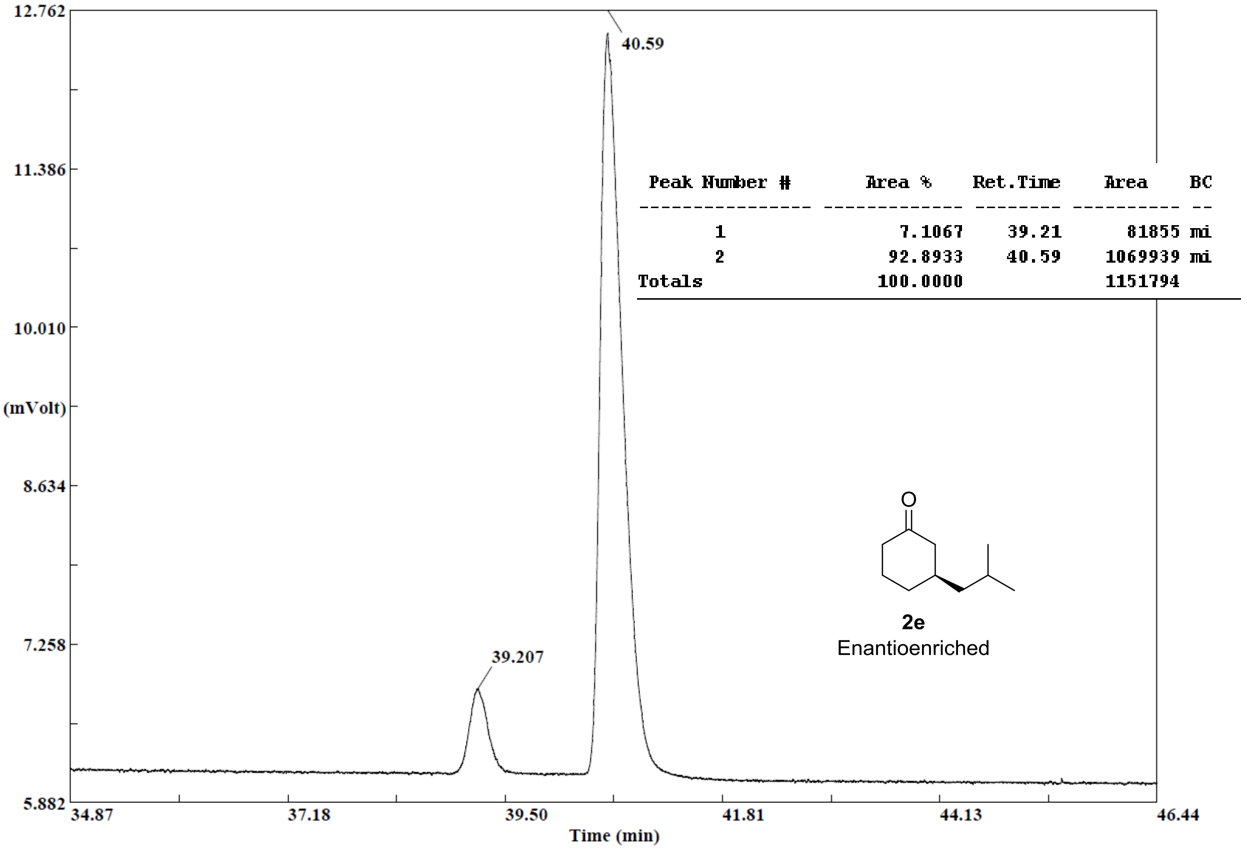

Peak Number #	Area %	Ret.Time	Area	BC
1	7.1067	39.21	81855	mi
2	92.8933	40.59	1069939	mi
Totals	100.0000		1151794	

2e
Enantioenriched

Filename D:\...\FS_180_P_3iBuCH_70_110_05r_99min.dat
Sample name :FS 180 P   Analysed :30.04.2019 10:58

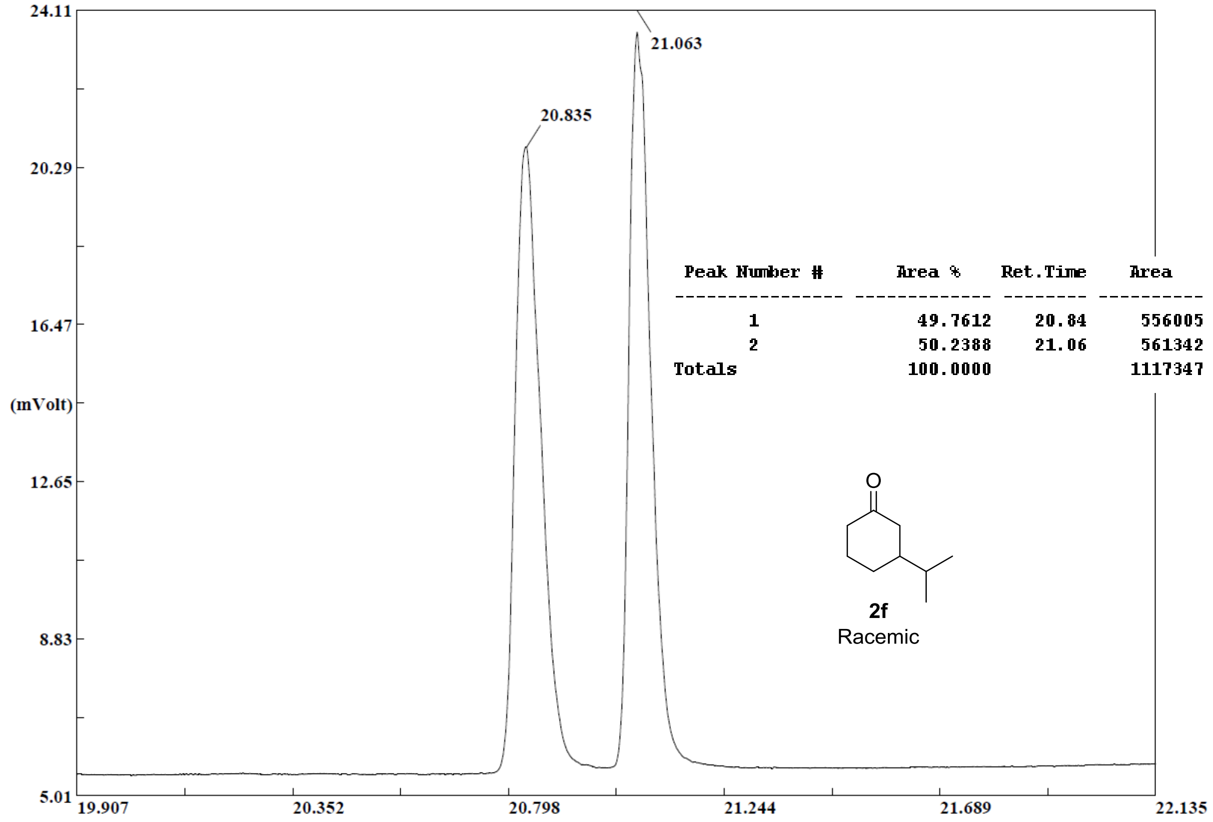

Peak Number #	Area %	Ret.Time	Area	BC
1	49.7612	20.84	556005	mi
2	50.2388	21.06	561342	mi
Totals	100.0000		1117347	

Filename D:\...\FS_116_A2_1-6_3iPrCH_85iso220_30r.dat
Sample name :FS 116 A Analysed :06.02.2019 11:11

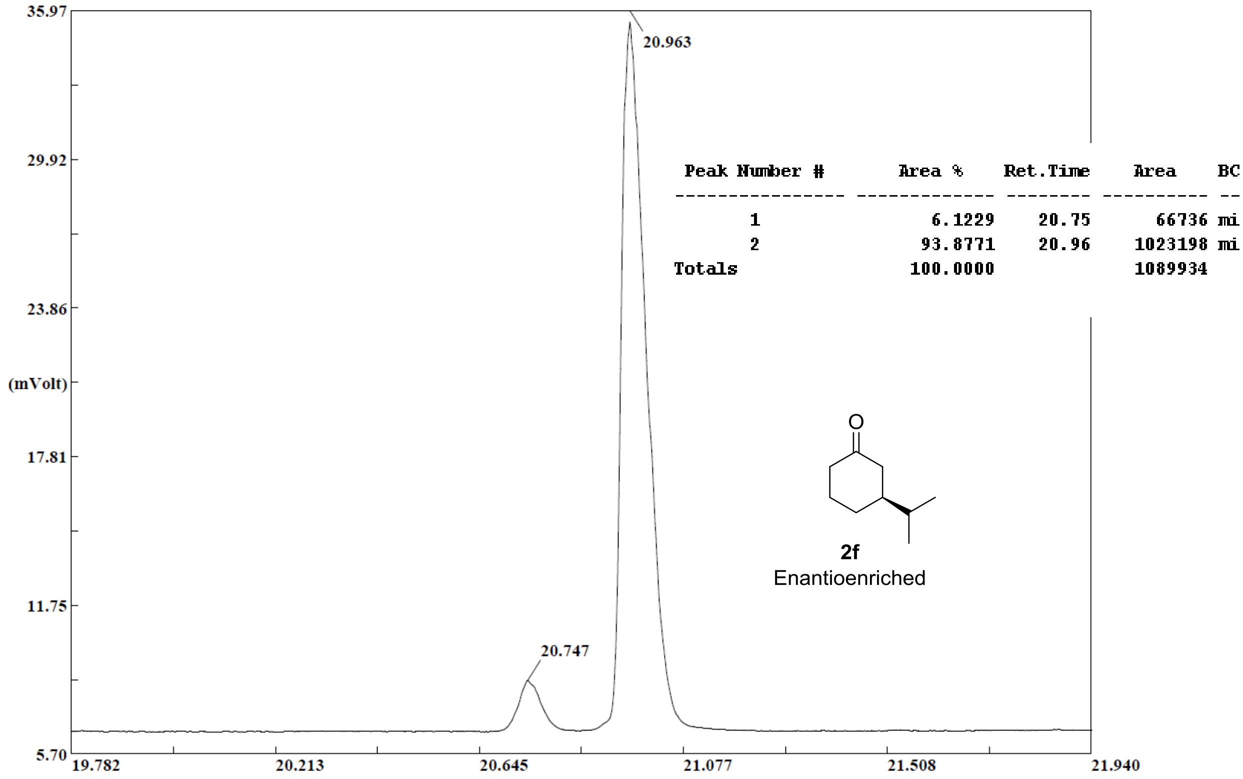

Peak Number #	Area %	Ret.Time	Area	BC
1	6.1229	20.75	66736	mi
2	93.8771	20.96	1023198	mi
Totals	100.0000		1089934	

Filename D:\...\FS_189_3iPrCH_85iso220_22min.dat
Sample name :FS 189 3 Analysed :02.05.2019 12:41

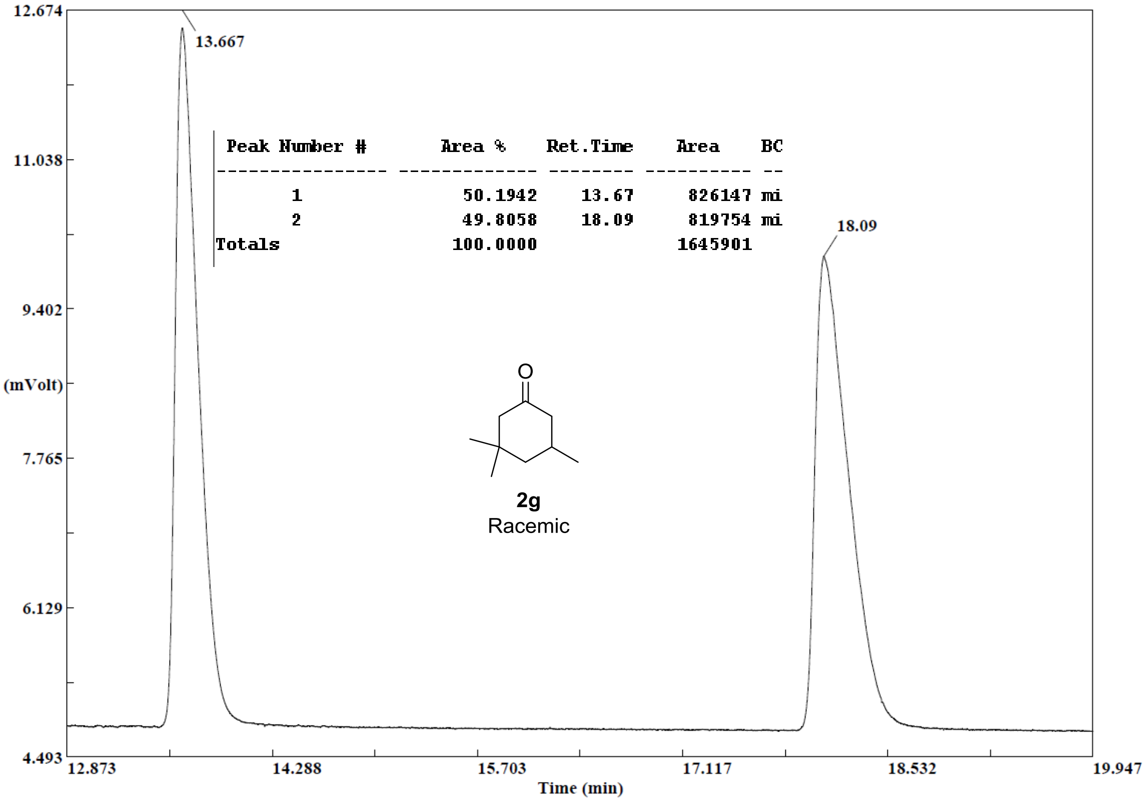

Peak Number #	Area %	Ret.Time	Area	BC
1	50.1942	13.67	826147	mi
2	49.8058	18.09	819754	mi
Totals	100.0000		1645901	

Filename D:\...\FS_116_A1_85iso220_30r.dat
Sample name :FS 116 A Analysed :06.02.2019 18:21

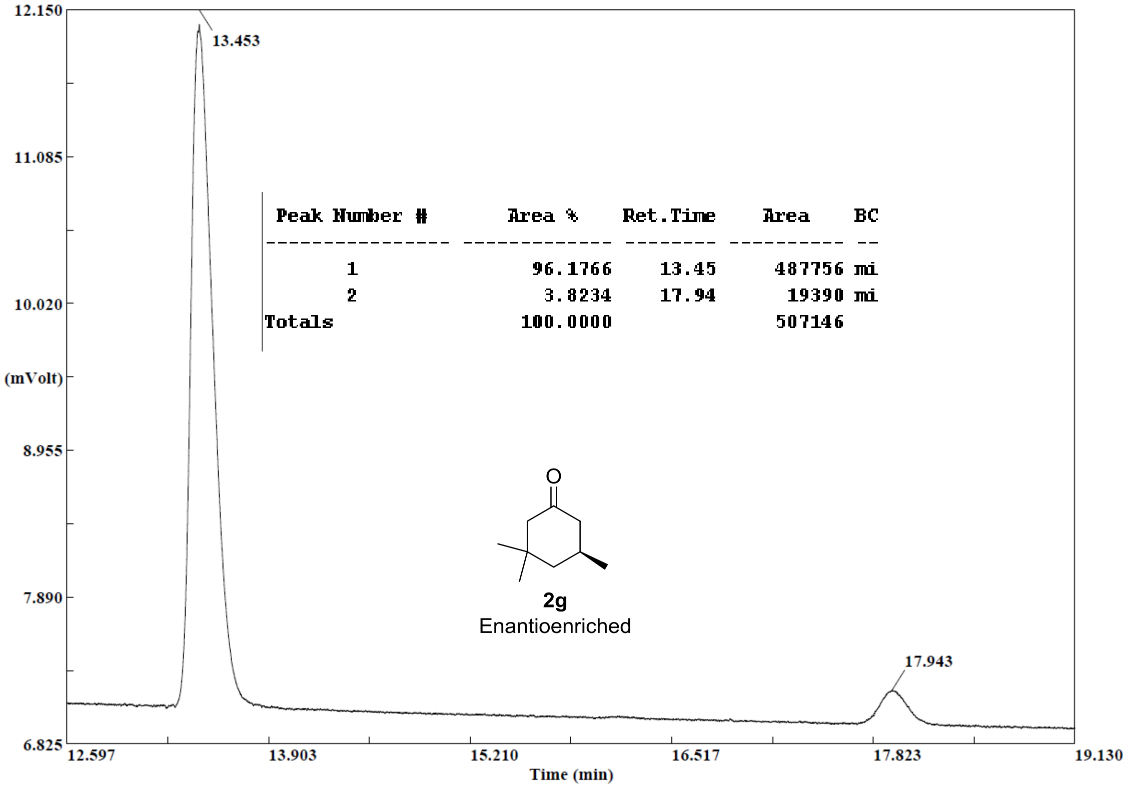

Peak Number #	Area %	Ret.Time	Area	BC
1	96.1766	13.45	487756	mi
2	3.8234	17.94	19390	mi
Totals	100.0000		507146	

Filename D:\...\FS_188_IP_85iso220_22min.dat
Sample name :FS_188_I Analysed :02.05.2019 12:11

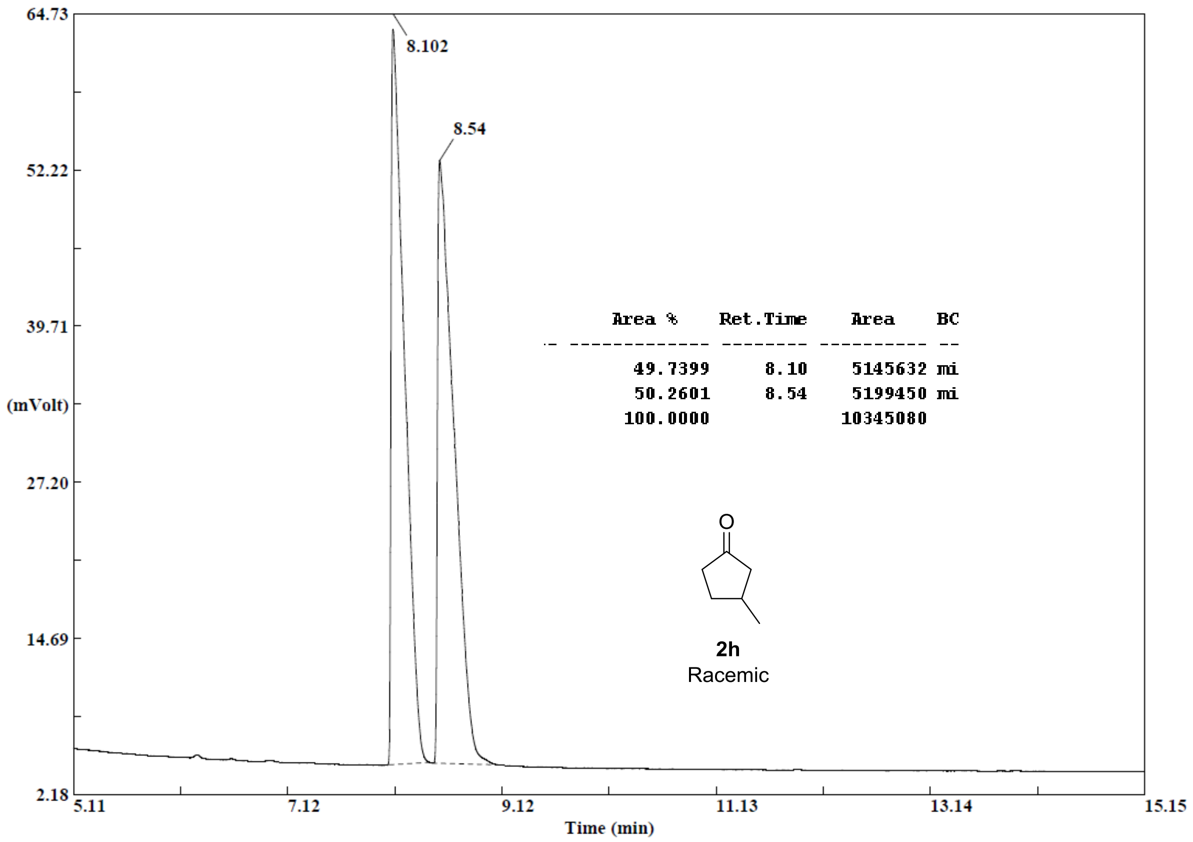
64.73
52.22
39.71
(mVolt)
27.20
14.69
2.18
5.11
7.12
9.12
11.13
13.14
15.15
Time (min)
8.102
8.54
Area % Ret.Time Area BC
49.7399 8.10 5145632 mi
50.2601 8.54 5199450 mi
100.0000 10345080
O
2h
Racemic

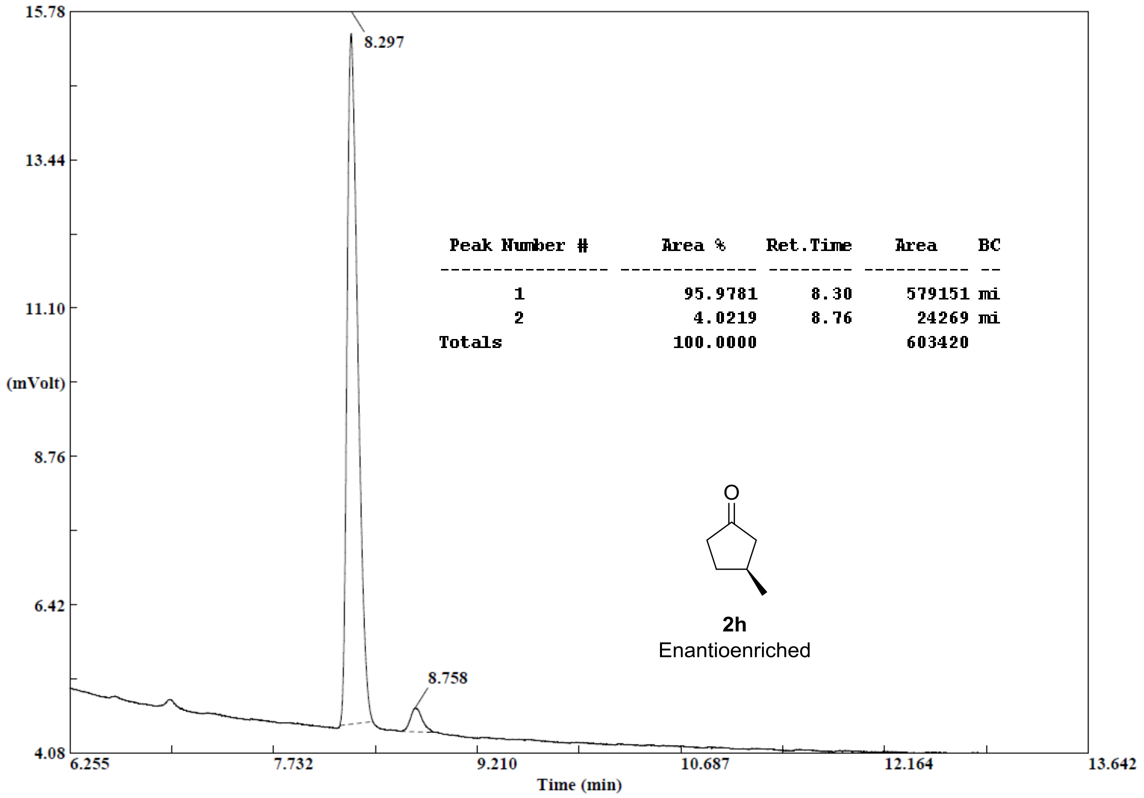
15.78
13.44
11.10
(mVolt)
8.76
6.42
4.08
6.255
7.732
9.210
10.687
12.164
13.642
Time (min)
8.297
8.758
Peak Number # Area % Ret.Time Area BC
1 95.9781 8.30 579151 mi
2 4.0219 8.76 24269 mi
Totals 100.0000 603420
O
2h
Enantioenriched

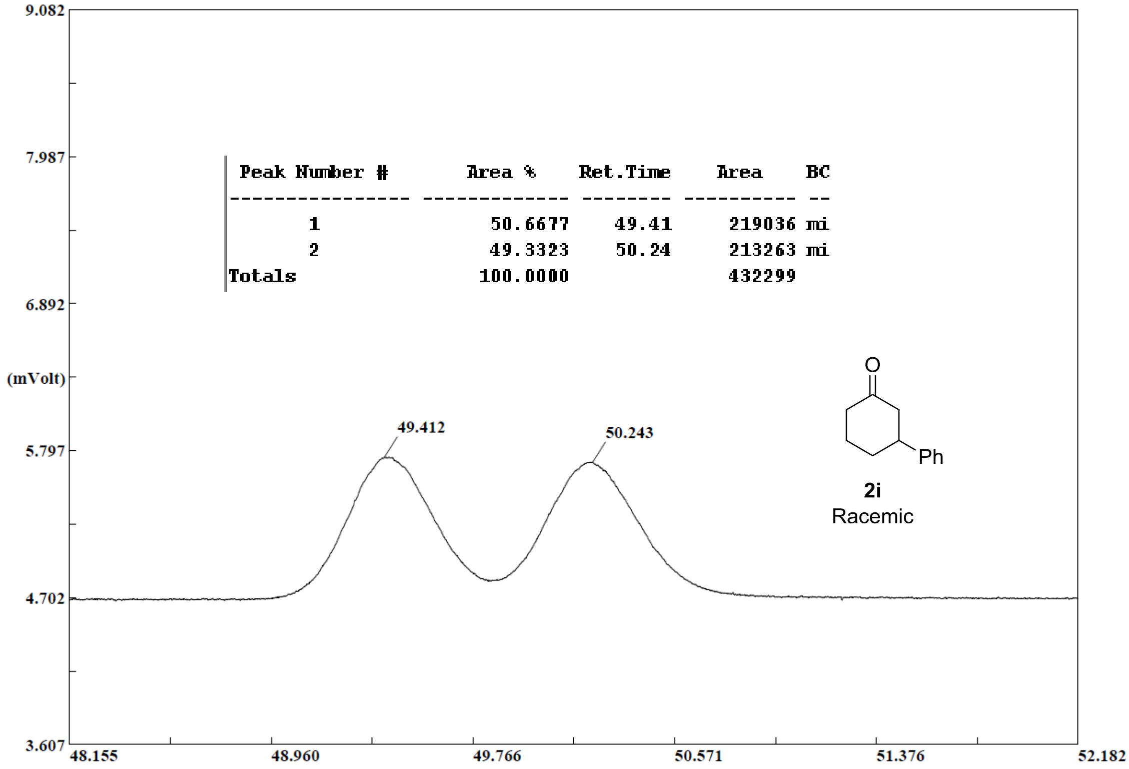

Peak Number #	Area %	Ret.Time	Area	BC
1	50.6677	49.41	219036	mi
2	49.3323	50.24	213263	mi
Totals	100.0000		432299	

Time (min)
Filename D:\...\FS_107_diluted_120iso220_30r_60min.dat
Sample name :FS_107_d Analysed :07.02.2019 10:07

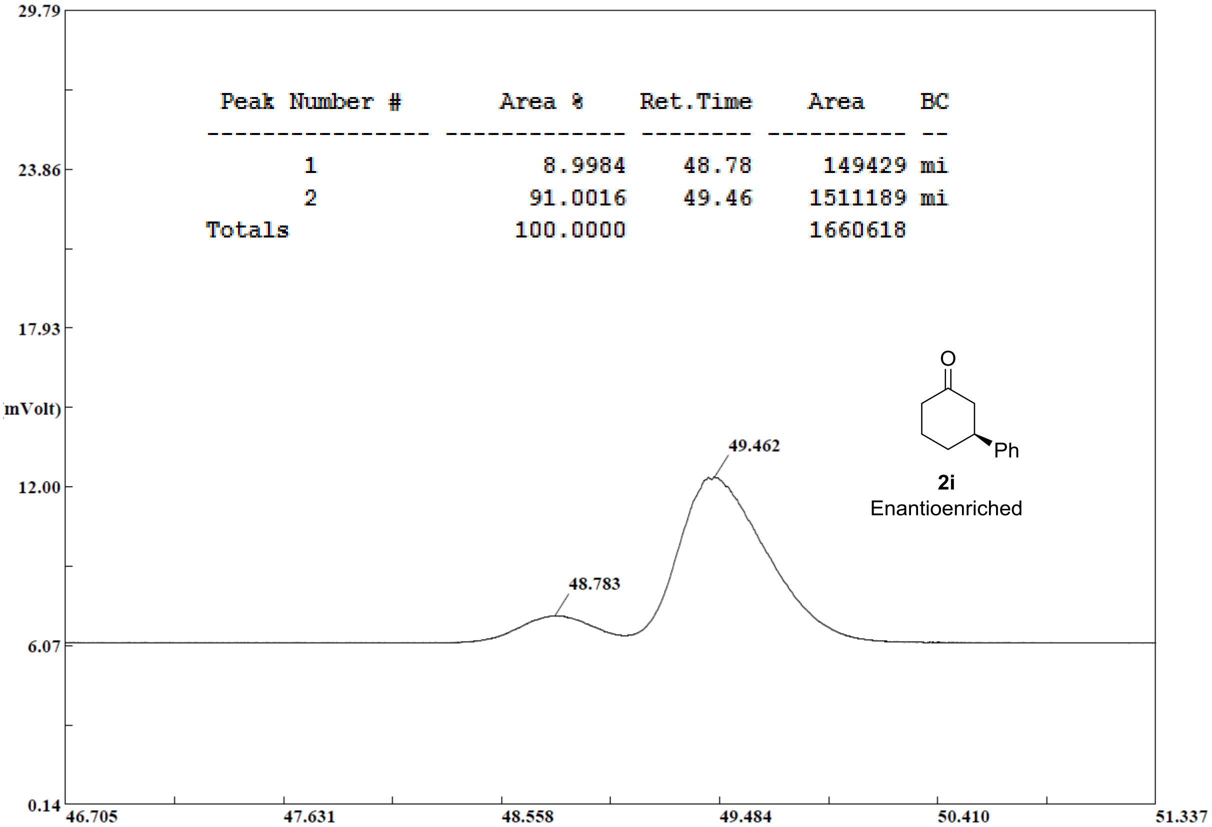

Peak Number #	Area %	Ret.Time	Area	BC
1	8.9984	48.78	149429	mi
2	91.0016	49.46	1511189	mi
Totals	100.0000		1660618	

Time (min)
Filename D:\...\FS_187_3PCH_120iso220_60min.dat
Sample name :FS 187 3 Analysed :02.05.2019 13:10

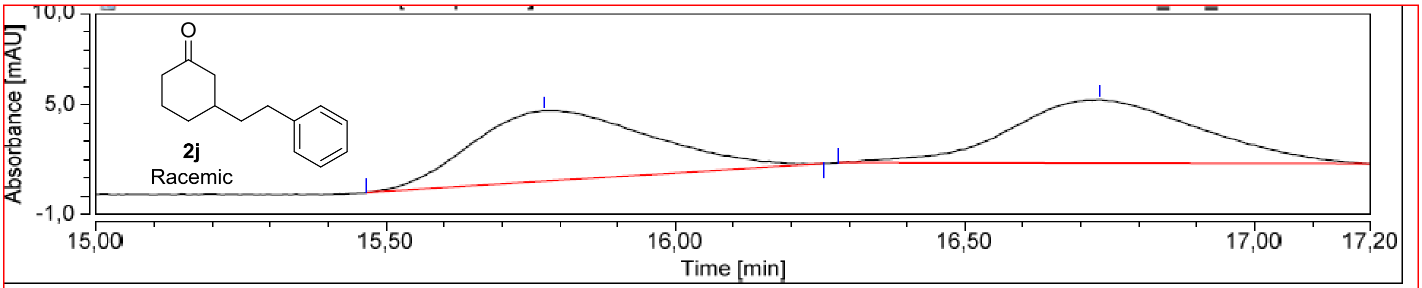

Integration Results

No.	Peak Name	Retention Time<br>min	Area<br>mAU*min	Height<br>mAU	Relative Area<br>%	Relative Height<br>%	Amount<br>n.a.
1		15,773	1,368	3,867	51,01	52,59	n.a.
2		16,732	1,314	3,486	48,99	47,41	n.a.
**Total:**			**2,682**	**7,353**	**100,00**	**100,00**	

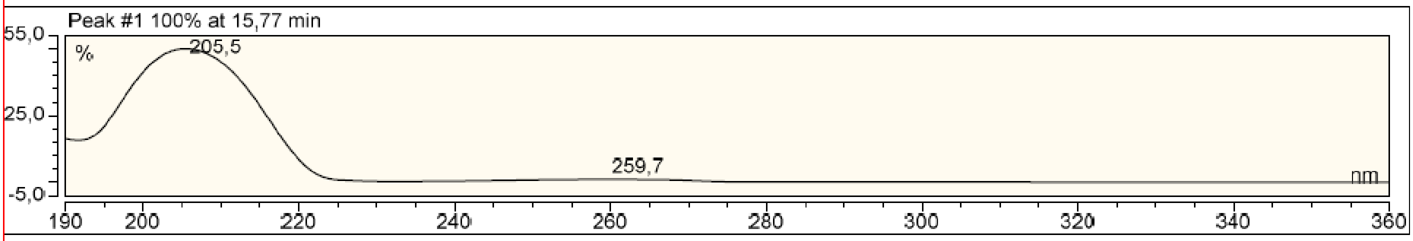

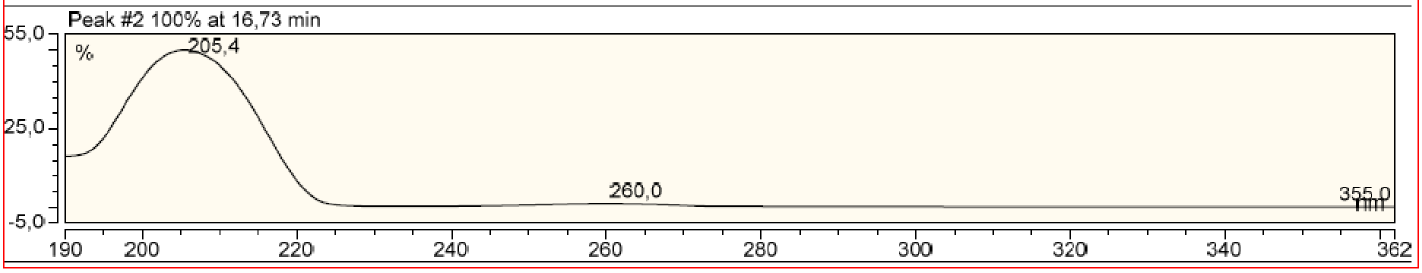

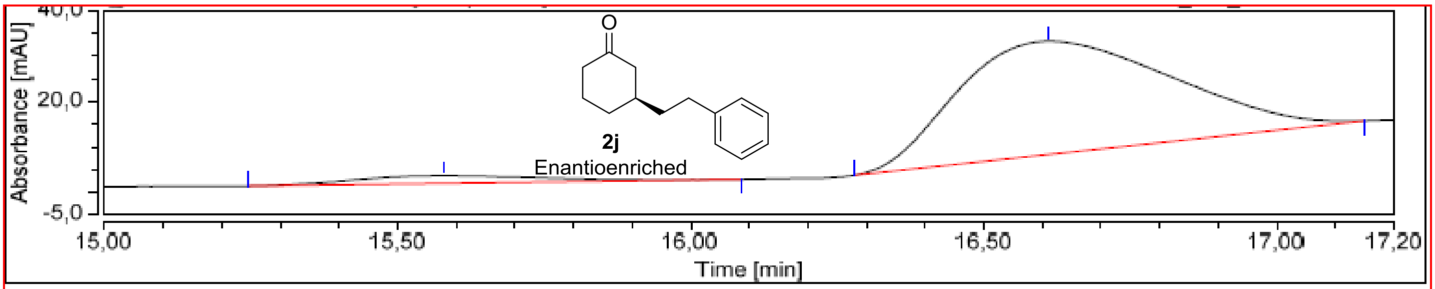

Integration Results

No.	Peak Name	Retention Time<br>min	Area<br>mAU*min	Height<br>mAU	Relative Area<br>%	Relative Height<br>%	Amount<br>n.a.
1		15,578	0,610	1,737	5,46	6,49	n.a.
2		16,610	10,569	25,025	94,54	93,51	n.a.
**Total:**			**11,179**	**26,762**	**100,00**	**100,00**	

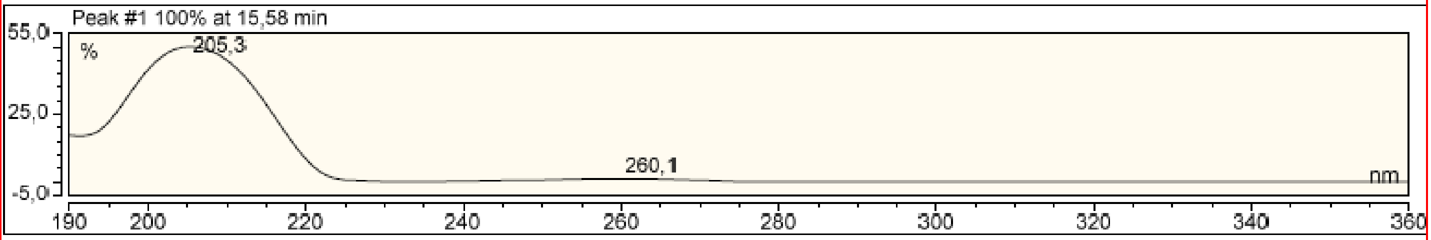

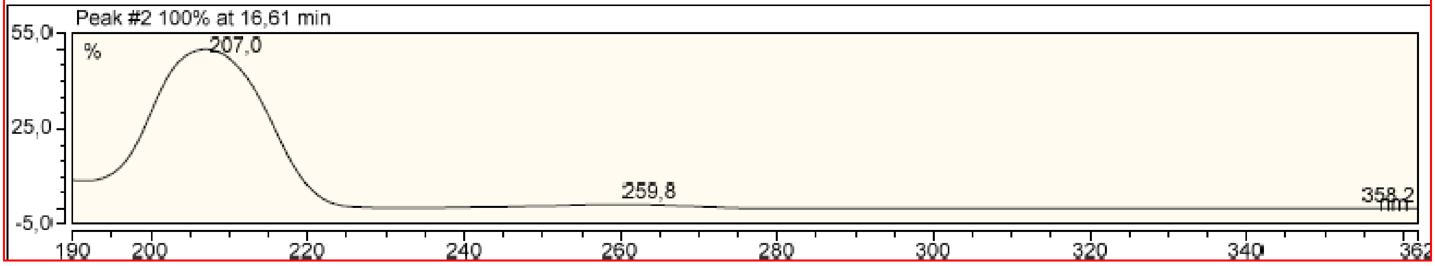

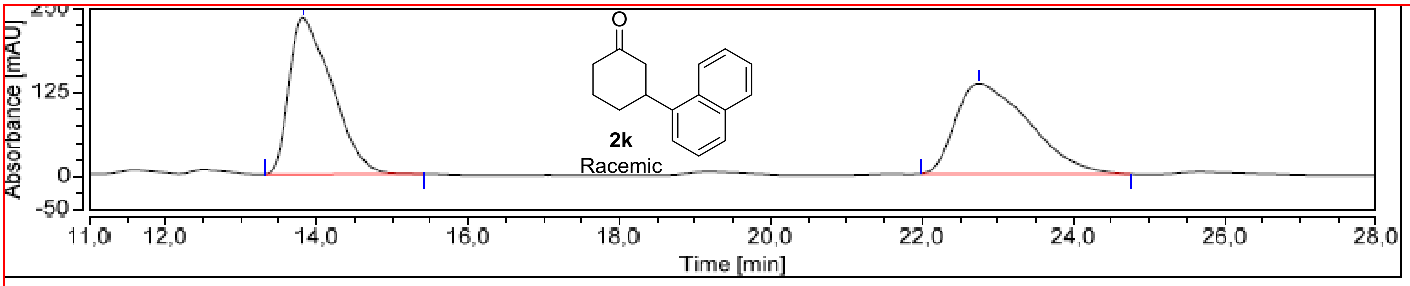

Integration Results

No.	Peak Name	Retention Time<br>min	Area<br>mAU*min	Height<br>mAU	Relative Area<br>%	Relative Height<br>%	Amount<br>n.a.
1		13,815	153,307	233,921	50,31	63,43	n.a.
2		22,743	151,414	134,857	49,69	36,57	n.a.
**Total:**			**304,720**	**368,778**	**100,00**	**100,00**	

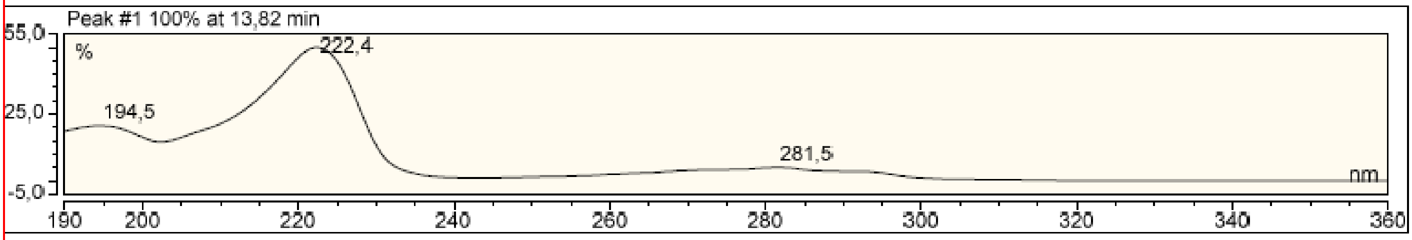

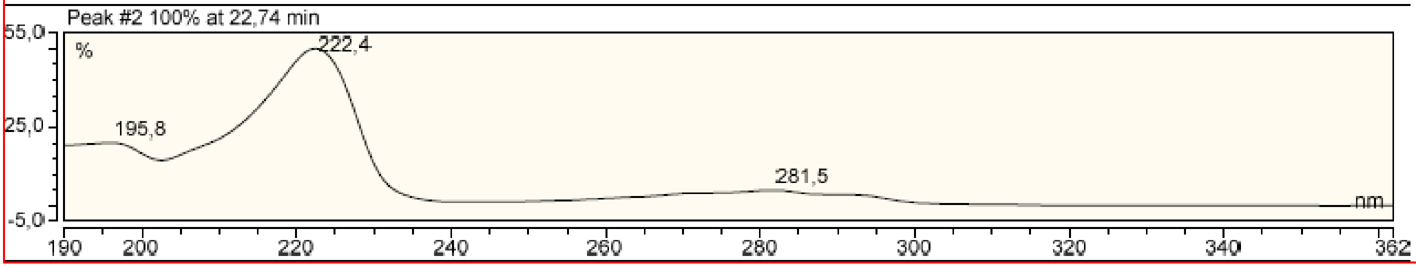

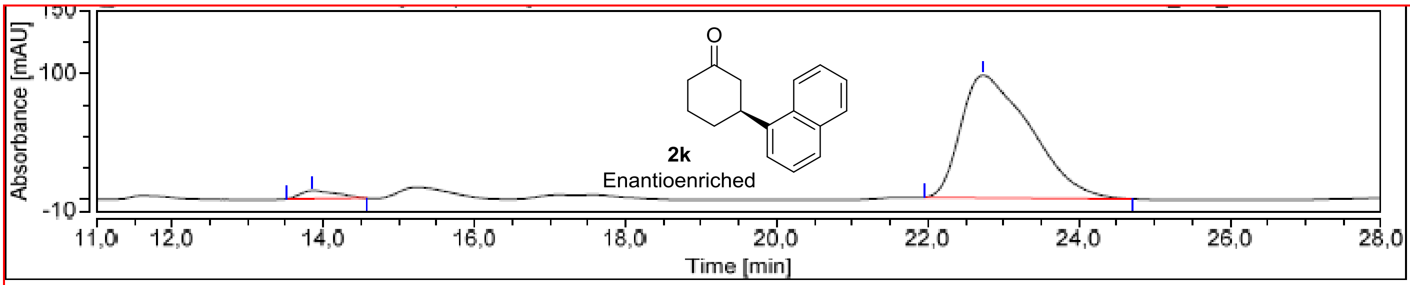

Integration Results

No.	Peak Name	Retention Time min	Area mAU*min	Height mAU	Relative Area %	Relative Height %	Amount n.a.
1		13,857	3,534	6,383	3,29	6,14	n.a.
2		22,732	103,836	97,530	96,71	93,86	n.a.
**Total:**			**107,370**	**103,913**	**100,00**	**100,00**	

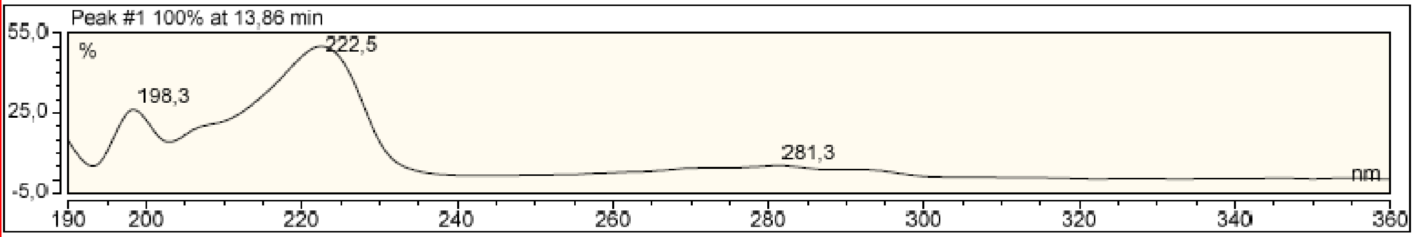

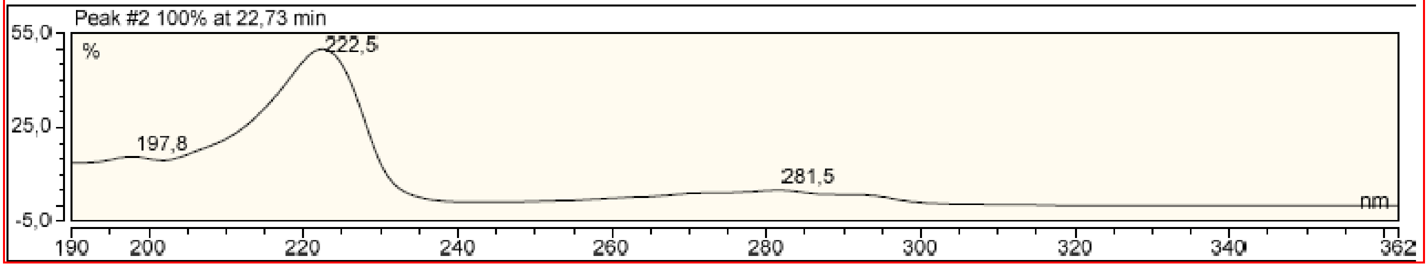

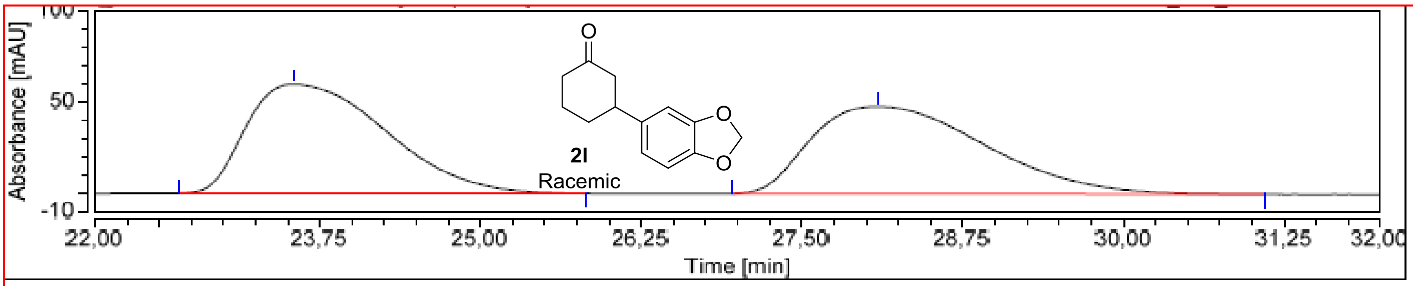

Integration Results

No.	Peak Name	Retention Time min	Area mAU*min	Height mAU	Relative Area %	Relative Height %	Amount n.a.
1		23,547	74,644	59,680	49,97	55,57	n.a.
2		28,085	74,741	47,719	50,03	44,43	n.a.
**Total:**			**149,386**	**107,399**	**100,00**	**100,00**	

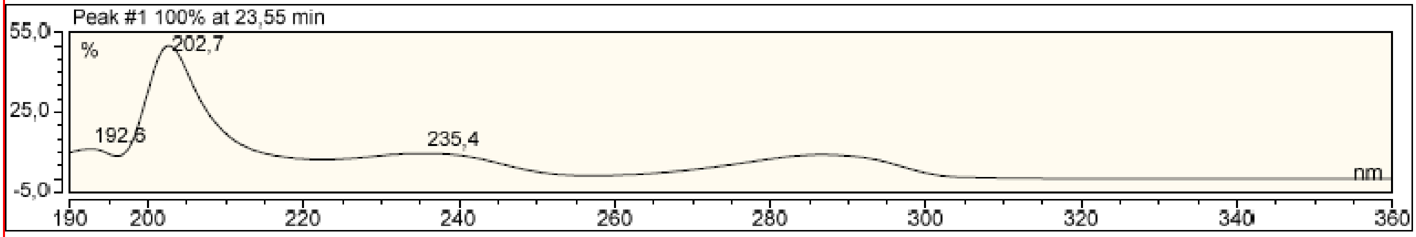

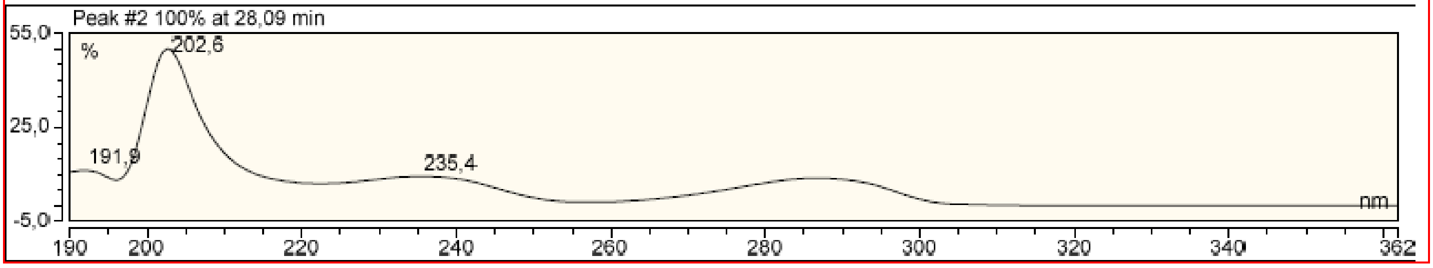

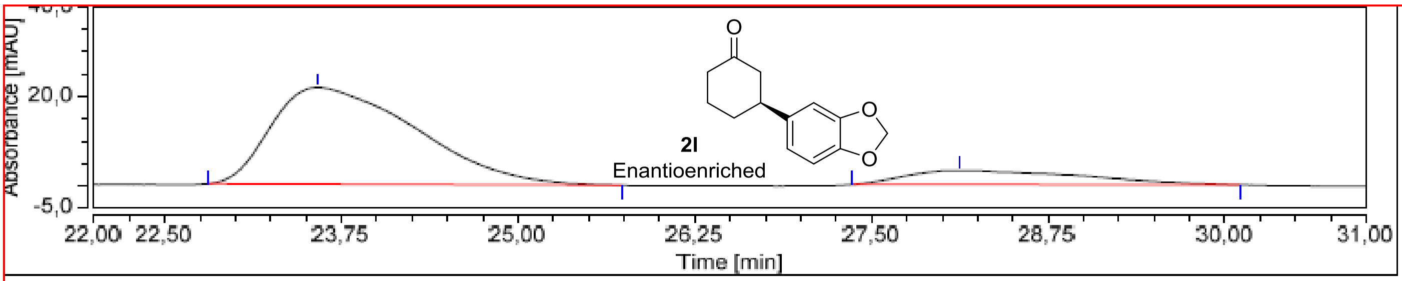

ntegration Results

No.	Peak Name	Retention Time<br>min	Area<br>mAU*min	Height<br>mAU	Relative Area<br>%	Relative Height<br>%	Amount<br>n.a.
1		23,583	25,752	21,686	84,92	87,25	n.a.
2		28,123	4,573	3,168	15,08	12,75	n.a.
**Total:**			**30,325**	**24,854**	**100,00**	**100,00**	

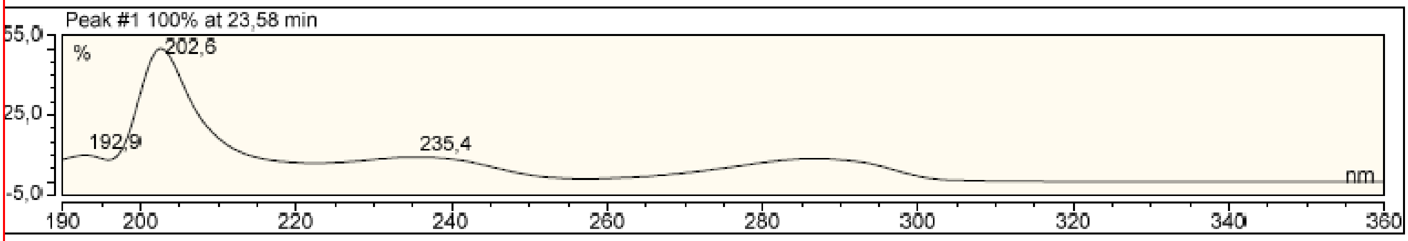

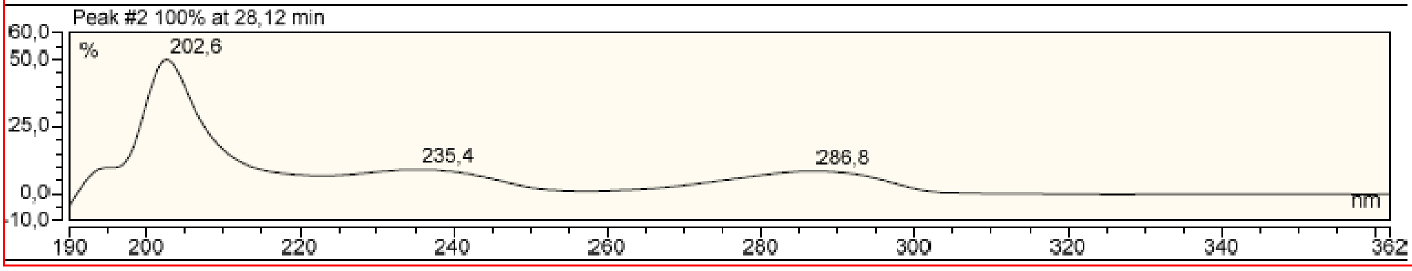

13. References

[1] H. Chen, Y. Feng, Z. Xu, T. Ye, *Tetrahedron* **2005**, *61*, 11132–11140.

[2] J. Han, J. Lian, X. Tian, S. Zhou, X. Zhen, S. Liu, *Eur. J. Org. Chem*. **2014**, *32*, 7232–7238.

[3] T. Akama et al. *Bioorg. Med. Chem. Lett*. **2018**, *28*, 6–10.

[4] S. Kandil, J. Balzarini, S. Rat, A. Brancale, A. D. Westwell, C. McGuigan, *Bioorg. Med. Chem. Lett.* **2016**, *26*, 5618–5623.

[5] S. Thétiot-Laurent et al. *ChemBioChem*. **2017**, *18*, 300-315.

[6] T. J. Trivedi, K. S. Rao, T. Singh, S. K. Mandal, N. Sutradhar, A. B. Panda, A. Kumar, *ChemSusChem.* **2011**, *4*, 604–608.

[7] J. You, B. Liu, Y. Wang, *J. Chin. Chem. Soc.-Taip.* **2009**, *56*, 1010-1017.

[8] W. Kuchen, H. F. *Phosphorus Sulfur*. **1980**, *8*, 139–145.

[9] S. Grant-Overton, J. A. Buss, E. H. Smith, E. G. Gutierrez, E. J. Moorhead, V. S. Lin, A. G. Wenzel, *Synth. Commun*. **2015**, *45*, 331–337.

[10] E. G. Gutierrez, E. J. Moorhead, E. H. Smith, V. S. Lin, L. K. G. Ackerman, C. E. Knezevic, V. Sun, S. Grant, A. G. Wenzel, *Eur. J. Org. Chem*. **2010**, *16*, 3027–3031.

[11] L. Iu, J. A. Fuentes, M. E. Janka, K. J. Fontenot, M. L. Clarke, *Angew. Chem. Int. Ed.* **2019**, *58*, 2120–2124.

[12] D. R. Armstrong, C. Cameron, D. C. Nonhebel, P. G. Perkins, *J. Chem. Soc.* **1983**, *5*, 587–589.

[13] R. Singh, C. Czekelius, R. R. Schrock, P. Müller, A. H. Hoveyda, *Organometallics* **2007**, *26*, 2528–2539.

[14] T. Wenliang, CAN149:307958, **2008**.

[15] Wenliang, T. *Production of Substituted Biphenyl Acid-Form Phosphate Ammonium Salt.* CAN149:307958, **2008**.

[16] L. M. Schneider, V. M. Schmiedel, T. Pecchioli, D. Lentz, C. Merten, M. Christmann, *Org. Lett.* **2017**, *19*, 2310–2313.

[17] N. J. A. Martin, B. List, *J. Am. Chem. Soc.* **2006**, *128*, 13368–13369.

[18] B.-D. Chong, Y.-I. Ji, S.-S. Oh, J.-D. Yang, W. Baik, S. Koo, *J. Org. Chem.* **2002**, *62*, 9323–9325..

[19] E. Brenna, M. Crotti, M. De Pieri,F. G. Gatti, G. Manenti, D. Monti, *Adv. Synth. Catal.* **2018**, *360*, 3677–3686.

[20] S. T. Cohen-Anisfeld, P. T. Lansbury, *J. Am. Chem. Soc.* **1993**, *115*, 10531–10537.

[21] M. D´Augustin, L. Palais, A. Alexakis, *Angew. Chem.* **2005**, *117*, 1400–1402.

[22] J.-G. Jun, T. H. Ha, B. P. Mundy, K. E. Bartelt, R. S. Bain, J. H. Cardellina, *J. Chem. Soc. Perkin Trans. 1* **1994**, 18, 2643.

[23] E. D. Scott, N. Amishiro, S. E. Denmark, N. Amishiro, *J. Org. Chem.* **2003**, *68* (18), 6997–7003.

[24] V. Jurkauskas, J. P. Sadighi, S. L. Buchwald, *Org. Lett*. **2003**, *5*, 2417-2420.

[25] Y. Fall, H. Doucet, M. Santelli, *Tetrahedron* **2009**, *2*, 489-495.

[26] M. K. Das, S. De, Shubhashish; A. Bisai, *Org. Biomol. Chem*. **2015**, *13*, 3585-3588.

[27] F. Cecacci, G. Mancini, P. Mencarelli, C. Villani, *Tetrahedron: Asymmetry* **2003**, *14*, 3117–3122.

[28] L. Luzzani, M. Mancinelli, A. Mazzanti, S. Lepri, R. Ruzziconi, M. Schlosser, *Org. Biomol. Chem.* **2012**, *10*, 1847–1855.

[29] R. Sahnoun, S. Koseki, Y. Fujimura, *J. Phys. Chem. A* **2006**, *110*, 2440–2447.

[30] E. Masson, *Org. Biomol. Chem.* **2013**, *11*, 2859.

[31] A. D. Becke, *J. Chem. Phys.* **1993**, *98*, 5648–5652.

[32] M. P. Johansson, J. Olsen, *J. Chem. Theory Comput.* **2008**, *4*, 1460–1471.

[33] S. Grimme, J. Antony, S. Ehrlich, H. Krieg, *J. Chem. Phys.* **2010**, *132*, 154104.

[34] S. Grimme, S. Erlich, L. Georigk, *J. Comput. Chem* **2011**, *32*, 1456–1465.

[35] S. H. Vosko, L. Wilk, M. Nusair, *Can. J. Phys.* **1980**, *58*, 1200–1211.

[36] F. Weigend, R. Ahlrichs, *Phys. Chem. Chem. Phys.* **2005**, *7*, 3297–3305.

[37] F. Neese, *Wiley Interdisciplinary Reviews: Computational Molecular Science* **2012**, *2*, 73–78.

[38] C. Riplinger, F. Neese, *J. Chem. Phys.* **2013**, *138*, 034106.

[39] J. E. Leffler, B. M. Graybill, *J. Phys. Chem.* **1959**, *63*, 1457–1460.

[40] G. Henkelman, B. P. Uberuaga, H. J. Jónsson, *Chem. Phys.* **2000**, *113*, 9901–9904.

[41] D. Sheppard, R. Terrell, G. Henkelman, *J. Chem. Phys.* **2008**, *128*, 134106.

[42] G. Baddeley, *Nature* **1946**, *157*, 694–695.

[43] C. C. K. Ling, M. M. Harris, *J. Chem. Soc.* **1964**, *0*, 1825–1835.

[44] K. Vanommeslaeghe, A. D. MacKerell, *J. Chem. Inf. Model.* **2012**, *52*, 3144–3154.

[45] K. Vanommeslaeghe, E. P. Raman, A. D. MacKerell, *J. Chem. Inf. Model* **2012,** *52*, 3155–3168.

[46] K. Vanommeslaeghe, E. Hatcher, C. Acharya, S. Kundu, S. Zhong, J. Shim, E. Darian, O. Guvench, P. Lopes, I. Vorobyov et al., *J. Comput. Chem* **2010**, *31*, 671–690.

[47] B. R. Brooks, C. L. Brooks, A. D. Mackerell, L. Nilsson, R. J. Petrella, B. Roux, Y. Won, G. Archontis, C. Bartels, S. Boresch et al., *J. Comput. Chem* **2009**, *30*, 1545–1614.

[48] L. Martínez, R. Andrade, G. Birgin, J. M. Martínez*, J. Comput. Chem*. **2009**, *30*, 2157-2164.

[49] N. Michaud-Agrawal, E. J. Denning, T. B. Woolf, O. Beckstein, *J. Comput. Chem* **2011**, *32*, 2319–2327.

[50] H. Wickham. ggplot2: Elegant Graphics for Data Analysis. Springer-Verlag New York, 2016.

[51] The Open Babel Package, version 2.4.1 https://openbabel.readthedocs.io/en/latest/3DStructureGen/multipleconformers.html
